# Prime Editor 3 Mediated Beta-Thalassemia Mutations of the *HBB* Gene in Human Erythroid Progenitor Cells

**DOI:** 10.3390/ijms23095002

**Published:** 2022-04-30

**Authors:** Haokun Zhang, Qinlinglan Zhou, Hongyan Chen, Daru Lu

**Affiliations:** 1 State Key Laboratory of Genetic Engineering, MOE Engineering Research Center of Gene Technology, School of Life Sciences, Fudan University, Shanghai 200438, China; zhanghaokun666@hotmail.com (H.Z.); 21210700056@m.fudan.edu.cn (Q.Z.); 2 NHC Key Laboratory of Birth Defects and Reproductive Health, Chongqing Key Laboratory of Birth Defects and Reproductive Health, Chongqing Population and Family Planning, Science and Technology Research Institute, Chongqing 404100, China

**Keywords:** prime editor 3, beta-thalassemia mutation, the *HBB* gene, HUDEP-2 cells, genome editing

## Abstract

Recently developed Prime Editor 3 (PE3) has been implemented to induce genome editing in various cell types but has not been proven in human hematopoietic stem and progenitor cells. Using PE3, we successfully installed the beta-thalassemia (beta-thal) mutations in the HBB gene in the erythroid progenitor cell line HUDEP-2. We inserted the *mCherry* reporter gene cassette into editing plasmids, each including the prime editing guide RNA (pegRNA) and nick sgRNA. The plasmids were electroporated into HUDEP-2 cells, and the PE3 modified cells were identified by mCherry expression and collected using fluorescence-activated cell sorting (FACS). Sanger sequencing of the positive cells confirmed that PE3 induced precise beta-thal mutations with editing ratios from 4.55 to 100%. Furthermore, an off-target analysis showed no unintentional edits occurred in the cells. The editing ratios and parameters of pegRNA and nick sgRNA were also analyzed and summarized and will contribute to enhanced PE3 design in future studies. The characterization of the HUDEP-2 beta-thal cells showed typical thalassemia phenotypes, involving ineffective erythropoiesis, abnormal erythroid differentiation, high apoptosis rate, defective alpha-globin colocalization, cell viability deterioration, and ROS resisting deficiency. These HUDEP-2 beta-thal cells could provide ideal models for future beta-thal gene therapy studies.

## 1. Introduction

Beta-thalassemia (beta-thal) is a common genetic disease caused by one or several nucleotide substitutions, insertions, or deletions within the *HBB* (beta-globin) gene [1,2,3,4,5]. Beta-thal is mainly disturbed in North America, Western Europe, the Mediterranean, the Indian subcontinent, and Southeast Asia [4,5,6]. Beta-thal results from numerous genotypes and over 300 alleles have been identified that are phenotypically categorized as severe (beta^0^), moderate (beta^+^), or silent (beta^++^), depending on the level of HBB reduction [3,5]. Mutations that hardly express the *HBB* gene resulting in no beta-globin production are classified as beta^0^. Other mutations allow the production of some beta- globin are classified as beta^+^ or beta^++^, depending on the degree of quantitative reduction in the output of the beta-globin [3,5]. The deficiency in beta-globin results in the excessive accumulation of free alpha-globin that is responsible for the pathophysiology of beta-thal [2,3,5,7]. Notably, over 90% of beta-thal, including the most twenty prevalent alleles (Table 1), are caused by mutations involving one or a limited number of nucleotides within the *HBB* gene or its immediate flanking regions [8,9]. Therefore, the modification of beta-thal mutations using genome editing tools has been suggested as a potential clinical treatment for the disease [10,11,12,13,14,15].

The recently developed Prime Editor 3 (PE3) can efficiently install all 12 types of point mutations (all 6 base-pair conversions), small insertions, and small deletions in a targeted and precise manner [16,17,18]. PE3 combines an engineered nCas9-transcriptase fusion protein, a pegRNA, and a nick sgRNA. The pegRNA contains the sgRNA sequence targeting the genomic site with an extended sequence at the 3′ end (primer binding sequence + RT-temple). The nick sgRNA targets the non-edited strand for nicking, usually between 40 and 90 nt away from the pegRNA site [16]. PE3 has been utilized for editing the *HBB* gene and the Sickle cell disease mutation (E6V) in HEK293T cells was installed [16]. However, there are two primary issues for PE3-mediated *HBB* mutation editing in HEK293T cells. First, editing efficiencies may vary considerably in progenitor and hematopoietic stem cells [17,19]. Second, PE3 genome editing lacks plasmid construct design optimization and editing efficiency data regarding beta-thal mutagenesis [17,19]. To resolve these problems, we aimed to install the twenty most prevalent beta-thal mutations in hematopoietic progenitor cells using PE3 (Table 1).

An immortalized human umbilical cord blood-derived erythroid progenitor cell line (HUDEP-2) was utilized in this study as these cells express the genes of *HBA* (alpha-globin) and *HBB* to form hemoglobin A (HbA, alpha- and beta-globin tetramer), and erythroid differentiation can be induced *in vitro* [20,21]. Installing beta-thal mutations in HUDEP-2 cells should reduce HbA assembly due to the beta-thal mutation-derived *HBB* deficiency. The excess abundance of free alpha-globin could then lead to ineffective erythropoiesis, defective erythroid differentiation, and abnormal apoptosis in erythroid progenitor cells [14,22]. Therefore, the edited HUDEP-2 beta-thal cell line would be a valuable model for studying the molecular mechanisms underlying erythropoiesis, erythroid differentiation, and apoptosis. Moreover, the desired cell lines may contribute to clinical and therapeutic gene research by providing a hematopoietic cellular model of beta-thal.

## 2. Results

### 2.1. PE3 Effectively Induce Beta-Thal Mutations in HUDEP-2 Cells

**Figure 1:**
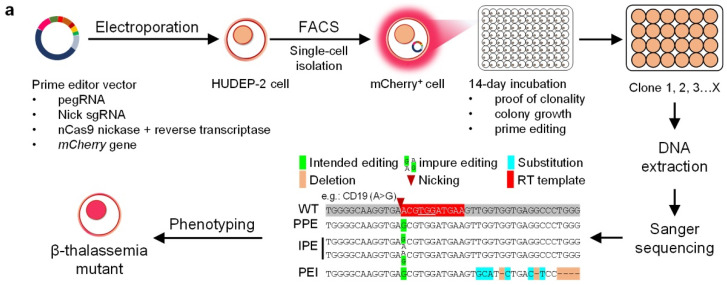

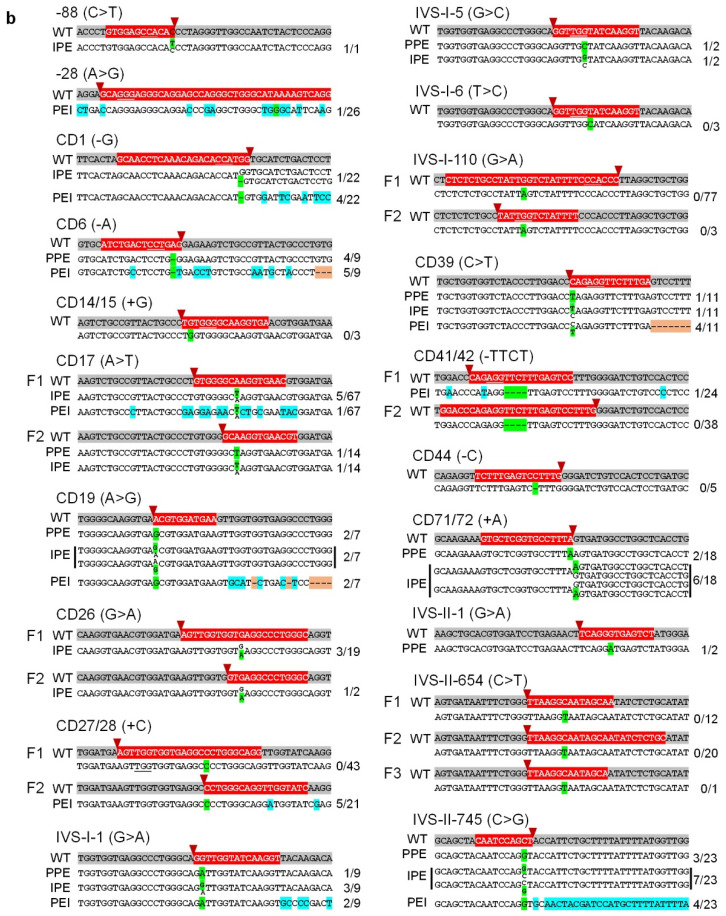
**Installation of beta-thal mutations in HUDEP-2 cells using PE3.** (**a**) Scheme of the workflow in this study. The constructed PE3 plasmids were utilized to install beta-thal mutations in HUDEP-2 cells. mCherry positive cells were sorted by FACS to rapidly detect PE3 modified HUDEP-2 cells that were selected for subsequent analyses. (**b**) The edits at each beta-thal mutation site, including editing position of pegRNA, Sanger sequencing result, sampling numbers, and the edit number.

We designed a method to install 20 beta-thal mutations in HUDEP-2 cells using a modified PE3 plasmid (Figure 1a and Figure S1a). Using the webtool pegFinder [23], for each mutation, a prime editing guide RNA (pegRNA) and nick sgRNA was designed for PE3 (Table S1). To generate each PE3 editing construct (Figure S1a), the reporter gene *mCherry*, a pegRNA, and a nick sgRNA were integrated into the pCMV-PE2 plasmids [16]. The modified PE3 plasmid was then transfected into the HUDEP-2 cell by electroporation. Approximately 48 h later, single cells expressing the *mCherry* gene were identified by fluorescence-activated cell sorting (FACS) (Figure S1b) and transferred to a 96-well plate for culture (Figure S1c). Once the cells reached a concentration of 1 × 10^5^, the genomic DNA was extracted, and PCR was performed. The PCR products of the positive clones were Sanger sequenced to confirm that the clone contained the correct on-target edits. Three types of on-target edit were classified using a criterion similar to Petri et al. [24]: the pure prime edits (PPEs)—alleles that only carry the intended edits without indels; the impure prime edits (IPEs)—alleles partly contain the intended edits without indels; the prime edits with indels (PEIs)—alleles that carry the intended edits with by-product edits and indels. The PPE and IPE clones were isolated and cultured for off-target analysis and phenotyping.

Using this method, PE3 successfully introduced the intended beta-thal mutations (including insertions, deletions, and substitutions) without indels in HUDEP-2 cells (Table 2, Figure 1b and Figure S1c). As shown in Table 2, PE3 installed all three edit types at CD6, CD17 F2, CD19, IVS-I-1, IVS-I-5, CD39, CD71/72, IVS-II-1, and IVS-II-745. PE3 induced IPEs and PEIs at -88, CD1, CD17 F1, and CD26, and created PEIs at -28, CD27/28 F2, and CD41/42 F1. In contrast, PE3 failed to induce the intended edits at CD14/15 (0/3), CD27/28 F1 (0/43), IVS-I-6 (0/3), IVS-I-110 (F1: 0/77, F2: 0/3), CD41/42 F2 (0/38), CD44 (0/5), and IVS-II-654 (F1: 0/12, F2: 0/20, F3: 0/1).

As shown in Table 2 and Figure S1c, the results illustrated that PE3 editing ratios varied depending on the specific target edits. The edits with high editing ratios were -88, CD6, CD19, IVS-I-1, IVS-I-5, CD71/72, IVS-II-1, and IVS-II-745, whereas those with low editing ratios were CD1, CD17, CD26 F1, and CD39. To optimize the editing ratio and generate desired mutations, we redesigned the PE3 constructs by changing the spacer sequence of the respective pegRNAs, adjusting the RT-template length, and modifying the nick sgRNA (Table S1). The redesigned PE3 plasmids efficiently installed on-target edits in CD17 F2 and CD26 F2, but not in IVS-I-110, CD41/42, or IVS-II-654. These editing data were further analyzed in 2.2. to optimize PE3 editing ratios.

### 2.2. Data Analysis and Optimization of pegRNAs and Nick sgRNAs

**Figure 2:**
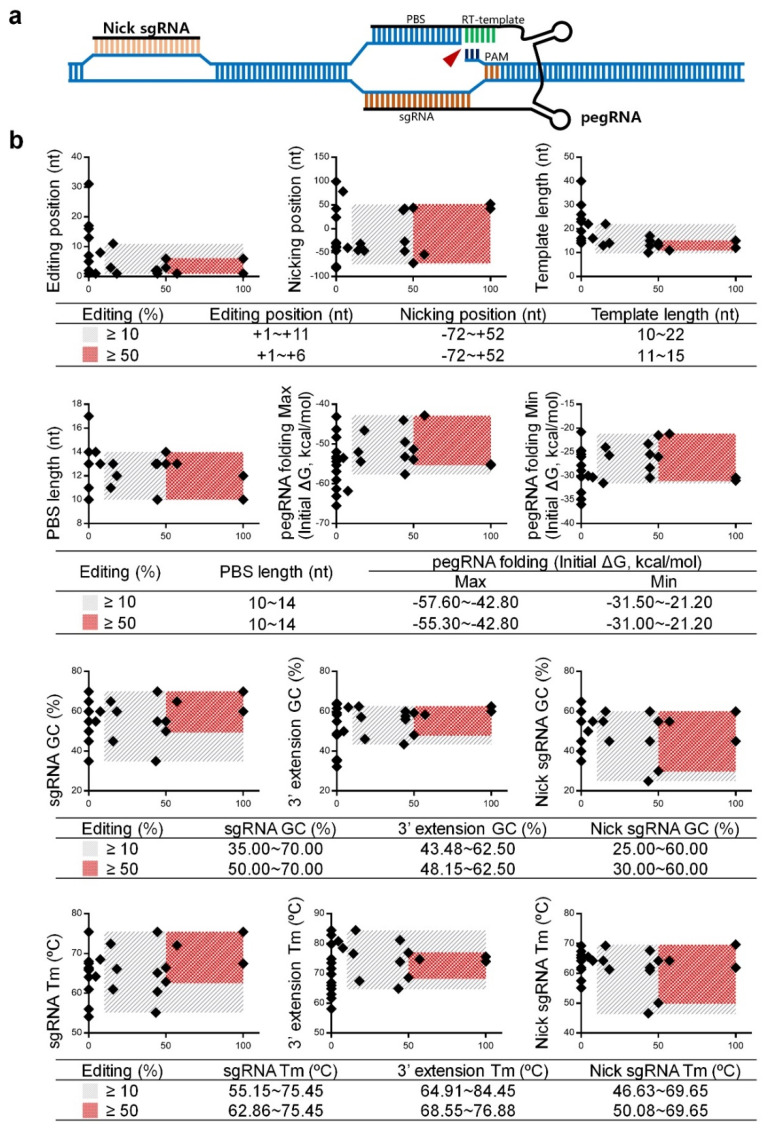
**Data analysis of pegRNAs and nick sgRNAs.** (**a**) Scheme of a PE3 construct. pegRNA combines a spacer sequence targeting the editing site and a 3′ extension sequence containing a primer binding sequence (PBS) fused to an RT-template with desired edits of interest. A nick sgRNA targets the non-edited strand for nicking, further increasing editing efficiency. (**b**) In this study, 26 different PE3 formulas were designed to induce beta-thal mutations. The *x*-axis indicates the editing ratio, whereas the *y*-axis indicates each detail of the PE3 formula, including editing position, nicking position, template length, PBS length, pegRNA folding energy, GC contents, and T_m_ values. The gray slash block indicates that the editing ratio is ≥10%, whereas the red dot matrix block indicates that the editing ratio is ≥50%.

As shown in Figure 2a, PE3 editing asks a designed pegRNA and a nick sgRNA targeting the non-edited strand [16]. As we constructed twenty-seven PE3 formulas to install beta-thal mutations in this study, these formulas showed variable editing ratios at the intended sites (Table 2 and Figure S1c). The equation, “editing% = (PPE + IPE)/total samples × 100%”, was used to compare the ratios among these formulas. Since PEI edits are difficult to determine, as the result of on-target editing by PE3 or homology-directed repair mediated by the nCas9 domain of the engineered nCas9-transcriptase fusion protein (Figure S1c), PEI is excluded from the calculation of the editing ratio. Therefore, the editing position, nicking position, template length, primer binding sequence (PBS) length, pegRNA folding energy, GC value, and T_m_ value are summarized in Table S2 for analyzing the parameters of pegRNA and nick sgRNA to achieve a higher editing ratio. We classified the editing ratio from 10–100% as “effective” and 50–100% as “efficient” because the corresponding PE3 formulas typically induce the desired PPEs and IPEs.

We plotted the rectangular coordinates with the editing ratio on the *x*-axis and each detail of PE3 formula on the *y*-axis (Figure 2b). These data showed that an “efficient” formula required more stringent pegRNA and nick sgRNA design including:

(a) pegRNA position is restricted to +1~+6 nt away from the nCas9 nicking site;
(b) template length is restricted to 11~15 nt;
(c) pegRNA folding requires less energy, with the upper and lower limits from −55.30~−42.80 and −31.00~−21.20 kcal/mol, respectively;
(d) GC content and T_m_ value of the sgRNA is restricted to 50.00~70.00% and 62.86~75.45 °C, respectively;
(e) GC content and T_m_ value of the 3′ extension is restricted to 48.15~62.50% and 68.55~76.88 °C, respectively;
(f) GC content and T_m_ value of the nicking sgRNA is restricted to 30.00~60.00% and 50.08~69.65 °C, respectively.

Notably, the data of IVS-II-654 F1~F3 (editing ratio = 0%) suggests that the GC content and T_m_ value of the 3′ extension sequence should not be lower than 40% and 63 °C (Figure 2b, Table S2). A low GC content and T_m_ may reduce the binding efficiency of the PBS to the antisense strand and subsequently interfere in the editing ratio.

### 2.3. Off-Target Analysis

**Figure 3:**
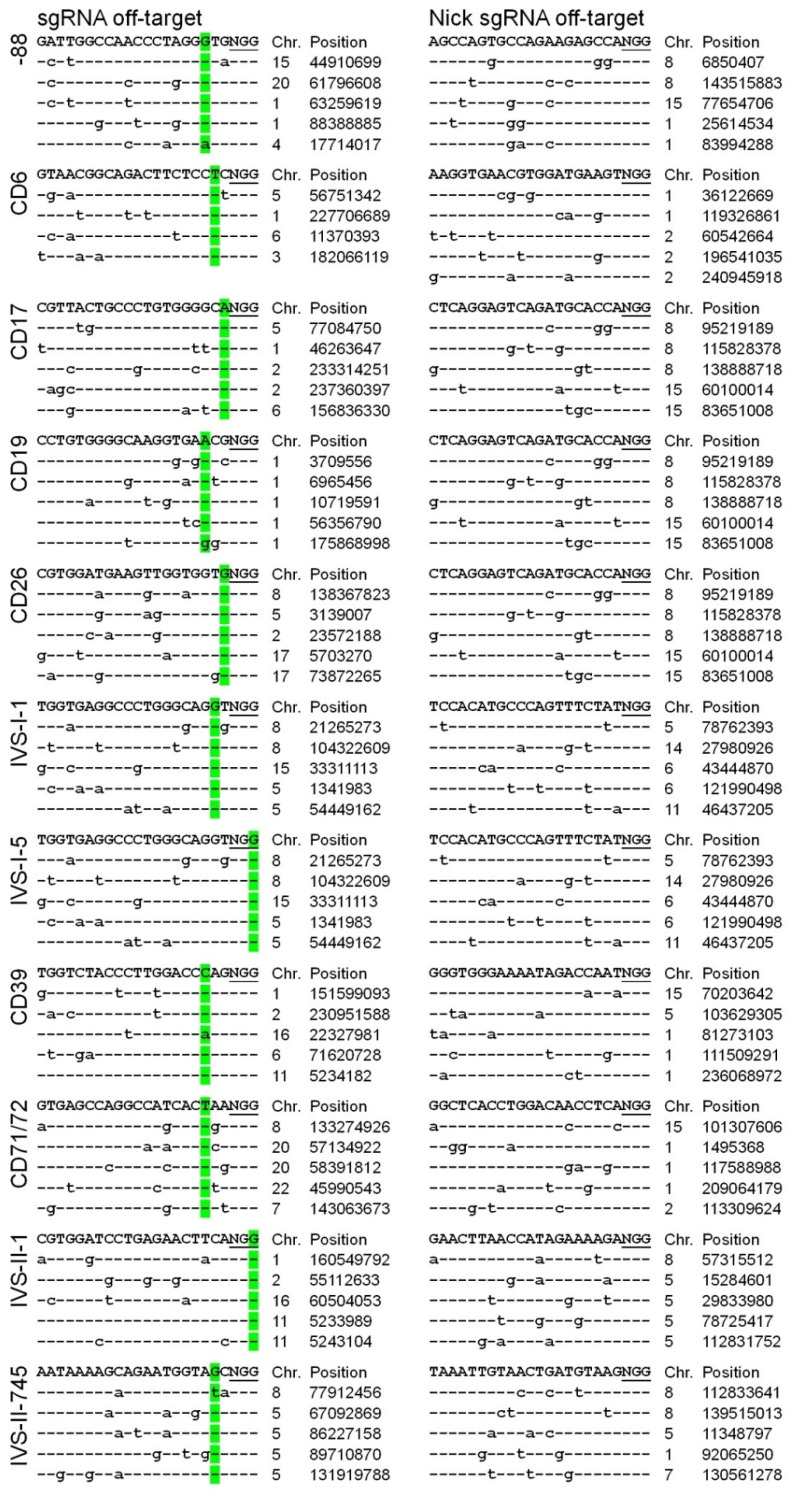
**PE3 off-target analysis.** Using the webtool Cas-OFFinder [25], off-targets were identified with at least three mismatches compared with the on-target sequences. This study selected the top five off-targets in the genome for each formula. The green fluorescence marks indicate the editing position of pegRNA, whereas the lowercase letters indicate mismatches.

As the single cell-derived HUDEP-2 clones were confirmed to contain the desired PPEs (CD6, CD17, CD19, IVS-I-1, IVS-I-5, CD39, CD71/72, IVS-II-1, and IVS-II-745) and IPEs (-88 and CD26), we performed an off-target analysis to identify whether the DNA modifications occurred in off-target sites of the genome. A recent study showed that PE3 could induce by-product edits at the nick sgRNA positions [24], so each beta-thal clone was analyzed simultaneously for off-target genome edits. Using the Cas-OFFinder tool [25], off-targets were identified with at least three mismatches compared with the on-target sequences (Table S3). We analyzed the top five sites ranked by all off-targets (Figure 3). Following genomic DNA extraction from the clones, the putative off-target sites were PCR-amplified and characterized by Sanger sequencing. No off-target DNA modifications were detected in the clones (Figure 3 and Figure S2). In future studies, whole-genome sequencing could be utilized to efficiently determine the frequency of off-target DNA modification following PE3 genome editing.

### 2.4. Characterization of the HUDEP-2 Beta-Thal Cells

We characterized the thalassemia phenotypes of the HUDEP-2 beta-thal clones with PPEs (CD6, CD17, CD19, IVS-I-1, IVS-I-5, CD39, CD71/72, IVS-II-1, and IVS-II-745) and IPEs (-88 and CD26).

**Figure 4:**
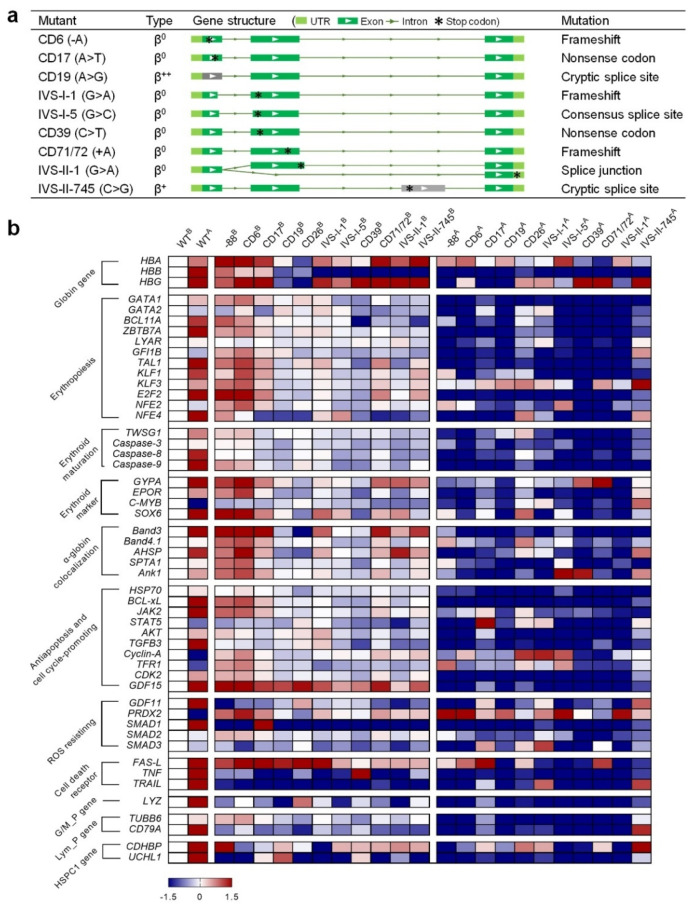

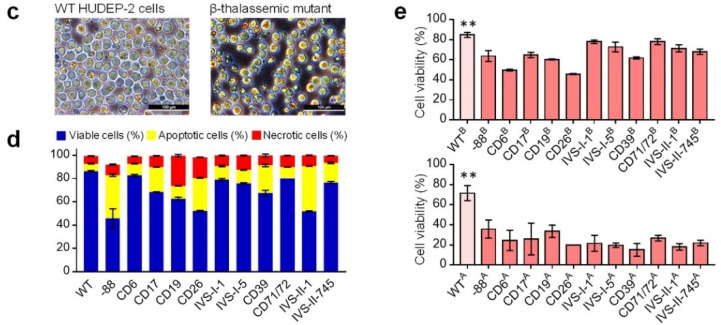
**Phenotyping of the HUDEP-2 cells with beta-thal mutations.** (**a**) Transcript structures of the *HBB* gene in beta-thal cells. The light green box indicates the UTR regions, green and gray boxes indicate the exon, the line indicates the intron, and the asterisk indicates the premature stop codon. Two isoforms with different lengths of *HBB* transcript were detected from IVS-II-1 cells. (**b**) Heatmap qPCR gene expression analysis. Superscript B and A mean before and after erythroid differentiation, respectively. All gene expression values were normalized to the *GAPDH* gene. The navy and crimson blocks indicate the genes are significantly downregulated and upregulated, respectively. (**c**) Microscopic examination of wildtype (WT) HDUEP-2 and beta-thal cells. More necrotic cells and debris are observed in beta-thal cells cultured in vitro. Scale bar: 100 μm. (**d**) Apoptosis analysis of WT HUDEP-2 and beta-thal cells cultured in vitro. *x*-axis shows different beta-thal cells, and *y*-axis shows the percentage of viable, apoptotic, and necrotic cells (mean ± SEM value). (**e**) Cell viability analysis of WT HUDEP-2 and beta-thal cells. ^B^ and ^A^ mean before and after erythroid differentiation, respectively. *x*-axis shows different beta-thal cells, and *y*-axis shows the percentage of cell viability (mean ± SEM value). ** means the *p*-value ≤ 0.01 (*t*-test).

A RT-PCR analysis of the *HBB* gene was performed in each mutant strain (Figure S3a,b). The results showed substitutions, insertions, and deletions were detected in the *HBB* genes for each PPE (Figure S3b) resulting in the generation of appropriately modified transcripts (Figure 4a). For the IPEs, the -88 (C>T) mutation was located in the transcription factor binding region. Therefore, the RT-PCR could not amplify the C>T substitution in the transcript (data not shown). The RT-PCR result of the modified CD26 (G>A) cells was almost identical to that of the wildtype (WT) HUDEP-2 cells. The sequencing traces showed a weak but detectable signal of adenine at the CD26 site (Figure S3b), indicating that a G>A substitution was introduced into the genome and generated the corresponding *HBB* transcript.

Next, we extracted the mRNA from WT HUDEP-2 and the beta-thal cells before and after erythroid differentiation (ED) (Figure S3c). Quantitative Real-Time PCR (qPCR) was performed to quantify the relative expression of the 51 genes associated with erythropoiesis and erythroid maturation [21,26,27,28,29,30,31,32,33,34,35,36,37,38,39]. The expression values were normalized with the housekeeping gene *GAPDH*, and the changes in gene expression were calculated (Table S6).

- WT *vs*. beta-thal before ED: value = ln (beta-thal^B^/WT^B^);
- WT *vs*. WT after ED: value = ln (WT^A^/WT^B^);
- WT *vs*. beta-thal after ED: value = ln (beta-thal^A^/WT^A^).

The results were plotted as a heatmap using GraphPad Prism (Figure 4b). The logarithm values of gene expression ranged from −1.5 (navy) to 1.5 (crimson), indicating that the corresponding genes were significantly down- or upregulated, respectively. After ED, most of the genes were upregulated in the WT cells, while several were downregulated, such as *GATA-2*, *c-MYB*, *cyclin-A*, and *PRDX2* (Figure 4b). Compared to the WT, the expression levels of most genes in beta-thal cells were moderately changed before ED. However, all the beta-thal clones showed downregulated expressions of *HBB* and the tested genes after ED. In addition, the alpha/beta-globin (*HBA*/*HBB*) mRNA ratios relative to *GAPDH* analyzed by qPCR showed significant imbalance between the alpha- and beta-chains in the beta-thal clones (Figure S4). These findings are consistent with the gene expression patterns in hematopoietic stem and progenitor cells from beta-thal patients [21,26,27,28,29,30,31,32,33,34,35,36,37,38,39,40]. The downregulation of these genes during erythropoiesis may be responsible for the subsequent adverse effects relating to erythroid maturation, free alpha-globin aggregation, antiapoptosis and cell cycling-promotion, ROS resistance, and programmed cell death.

The qPCR results showed that the expression of genes associated with cell death receptors fluctuate significantly in beta-thal cells before ED (Figure 4b), suggesting that programmed cell death is abnormal in those cells. We performed a microscopic examination and apoptosis analysis of abnormal apoptosis and cell death processes. The WT cells were chubby and spherical, whereas the beta-thal cells showed disparate sizes, and more necrotic cells were visible (Figure 4c). The apoptosis analysis showed that the beta-thal cells had a higher ratio of apoptotic and necrotic cells (Figure 4d and Figure S3d,e) and was consistent with the observations from patient-derived beta-thal hematopoietic stem and progenitor cells [31,32,33,40,41].

In addition, the qPCR results showed that the beta-thal cells had abnormal gene expressions profiles for antiapoptosis, cell cycling-promoting, and cell death receptors after ED (Figure 4b), suggesting ineffective erythropoiesis in beta-thal cells may exacerbate apoptosis and cell death. We performed a microscopic examination of beta-thal cells during erythropoiesis, showing that the beta-thal cell cultures bare more debris and necrotic cells than WT cell cultures (Figure S3f). We then examined the cell viability of WT and beta-thal cells before and after ED. The results showed that the viability of beta-thal cells decreased to varying degrees compared to WT but were maintained at a level greater than 45% compared to the cells before ED and decreased considerably (20–40%) after ED (Figure 4e and Figure S3g). These results verified our prediction that erythropoiesis exacerbates the death of HUDEP-2 beta-thal cells, which is consistent with the phenotype of massive apoptosis and cell death in thalassemia erythrocytes [31,32,40,41,42].

Our findings demonstrate that the installation of beta-thal mutations in HUDEP-2 cells results in typical thalassemia phenotypes.

## 3. Discussion

PE3 has proven to be an effective genome editing tool for mediating single point mutations and can install specific and targeted edits in various experimental models [16,17,24,43,44,45,46,47,48,49,50]. PE3 is, thus, thought to be more efficient than CRISPR-Cas9 induced homologous recombination edits and can accomplish more diverse genome editing tasks than the Base Editors [17]. However, PE3 mediated genome editing has limitations, including low editing efficiency and a challenging construct design [17,19,51]. This study adopted *mCherry* reporter gene screening to rapidly obtain edited HUDEP-2 cells. Construct editing ratios were also analyzed regarding the designed pegRNAs and nick sgRNAs to optimize the parameters required to consistently achieve a high editing ratio by PE3. Our data suggest that to avoid a low editing ratio, the GC content and T_m_ value of the 3′ extension sequences should not be lower than 40% and 63 °C. Further, our data analysis showed that the PE3 formulas with an editing ratio of <10% are often close to or exceed the reported parameters. For example, the editing position and template length installing the -28 mutation were +31 and 40 nt, respectively. The nick sgRNA positions and PBS length inducing the IVS-II-654 mutation were -88, +99, and 17 nt, respectively. The excessive PE3 parameters in -28 and IVS-II-654 formulas were mainly due to lack of accessibility for the gRNA, according to the prediction of webtool CRISPOR [52], to facilitate Cas9-induced editing at the intended target sites. The defective editing at the IVS-I-110 site was also due to a lack of accessibility for the sgRNA at the target site. These findings indicate that sgRNA accessibility is essential for prime editing. Together, the data of editing ratios and parameters of pegRNA and nick sgRNA will contribute to future PE3 design optimization. In the present study, we calculated the efficiency ratios based on the Sanger sequencing results of single cell-derived HUDEP-2 clones. However, an accurate PE3-mediated editing efficiency in HUDEP-2 cells or hematopoietic stem and progenitor cells should be addressed by using the next-generation sequencing-based method since some PEI samples (e.g., -28 PEI and CD6 PEIs in the Figure 1b and Figure S1c) cannot be determined as the results of on-target editing by PE3 or homology-directed repair induced by the nCas9 of PE fusion protein.

On the other hand, our data showed that low editing ratios at CD14/15, IVS-I-6, IVS-I-110, CD44, and IVS-II-654 may be also limited by deficient clones. Clone deficiency is likely due to a specific characteristic of the HUDEP-2 cells, which are prone to deteriorate at extra-low cell densities [20,21]. We also lost the clones of CD1 (IPE) and CD41/42 (PEI) due to poor cell viability. Maintaining the viability of HUDEP-2 cells after sorting by FACS will help optimize the output of the generated clones.

A recent study has shown that PE3 ribonucleoprotein (RNP) electroporation can efficiently introduce prime edits in germlines and primary cells [24]. In this study, PE3 efficiently installed intended edits on the *HBB* gene, mostly PPEs. Moreover, an off-target analysis showed no off-target editing of the genome. Our results suggest that PE3 has excellent potential for the gene therapy of beta-thal, and these data may contribute to the modification of *HBB* disease-causing beta-thal mutations using the PE3. Efficient gene therapies for beta-thal could be achieved with reduced safety concerns by the electroporation of PE3 RNP into patient-derived hematopoietic stem cells.

The phenotyping data of the HUDEP-2 beta-thal cells presented in this study involved ineffective erythropoiesis, abnormal erythroid differentiation, high apoptosis rate, defective alpha-globin colocalization, cell viability deterioration, and ROS resisting deficiency. Multi-omics studies of WT HUDEP-2 and beta-thal cells before and after erythroid differentiation will also help determine the molecular mechanisms underlying the pathological phenotypes of beta-thal and aid in identifying new targets for future genetic therapies [53]. The beta-thal cells generated here include the most prevalent beta-thal mutations globally and will provide ideal models for the development of genome editing tools to correct these debilitating mutations.

## 4. Materials and Methods

### 4.1. Cell Culture

HUDEP-2 cells were cultured using the proliferation medium described previously [21]. Briefly, StemSpan SFEM (Stemcell Technologies, Vancouver, British Columbia, Canada) was supplemented with dexamethasone (1 μM) (Novoprotein, Shanghai, China), doxycycline (1 μg/mL) (Novoprotein, Shanghai, China), erythropoietin (3 units/mL) (Novoprotein, Shanghai, China), human SCF (100 ng/mL) (Novoprotein, Shanghai, China), and 1% penicillin/streptomycin (Thermo Fisher Scientific, Waltham, MA, USA) in a humidified atmosphere of 5% CO_2_ at 37 °C. For the erythroid differentiation culture system, HUDEP2 cells were induced as described previously [21]. Briefly, HUDEP-2 cells were cultured in IMDM (Sigma, Kawasaki, Japan) containing 10% α-tocopherol (20 ng/mL) (Sigma, Kawasaki, Japan), linoleic acid (4 ng/mL) (Sigma, Kawasaki, Japan), cholesterol (200 ng/mL) (Sigma, Kawasaki, Japan), sodium selenite (2 ng/mL) (Sigma, Kawasaki, Japan), holo-transferrin (200 mg/mL) (Sigma, Kawasaki, Japan), human insulin (10 mg/mL) (Sigma, Kawasaki, Japan), ethanolamine (10 mM) (Sigma, Kawasaki, Japan), 2-ME (0.1 mM) (Sigma, Kawasaki, Japan), D-mannitol (14.57 mg/mL) (Sigma, Kawasaki, Japan), mifepristone (an antagonist of the glucocorticoid receptor, 1 mM) (Sigma, Kawasaki, Japan), and EPO (5 IU/mL) (Novoprotein, Shanghai, China).

### 4.2. PE3 Plasmid Construction and Purification

The PE3 systems, pegRNAs and nick sgRNAs, for beta-thal mutations (Table 1 and Table S1) were designed on the website of pegFinder [23]. The corresponding spacer sequences, 3′ extension sequences, and nick sgRNAs are shown in Table S1. The pegRNA plasmids were constructed as described previously [16]. Then, the pegRNA segments were PCR-amplified using the primer pair Mlu I-pegRNA listed in Table S7. The PCR reaction was performed using the KOD-neo-plus (TOYOBO, Shanghai, China). The pCMV-PE2 plasmid was linearized using the restriction endonuclease *Mlu* I (NEB, Ipswich, MA, USA). The reporter gene mCherry was amplified by PCR using the primer pair Mlu I-mCherry listed in Table S7. Then, the mCherry-pegRNA segments were amplified by PCR using the primers Mlu I-mCherry+ and MluI-pegRNA-. The combined segments were constructed into the *Mlu* I-linearized pCMV-PE2 plasmid using the NovoRec^®^ plus One step PCR Cloning Kit (Novoprotein, Shanghai, China). The pCMV-PE2-mCherry-pegRNA plasmids were linearized using the restriction endonuclease *Mlu* I (NEB, Ipswich, MA, USA). The nick sgRNA segments were amplified by PCR using the primer pair nick-Mlu I listed in Table S7. The nick sgRNA segments were then constructed into the *Mlu* I-linearized pCMV-PE2-mCherry-pegRNA plasmids using the NovoRec^®^ plus One step PCR Cloning Kit (Novoprotein, Shanghai, China). All PCR and linearization products were purified using the DNA Gel/PCR Purification Kit (Novoprotein, Shanghai, China). For plasmid amplification, the DH5α-competent cells (Novoprotein, Shanghai, China) transformed with plasmid were grown in LB media at 37 °C for 16–20 h. The plasmid was extracted using the PureLink HiPure Kit (Thermo Fisher Scientific, Waltham, MA, USA). The purified plasmid was concentrated in the elution buffer.

### 4.3. Establishment of HUDEP-2 Beta-Thal Mutant Clones

According to the manufacturer’s instructions, the electroporation was performed using the 20-μL Strips of the Celetrix basic model (Celetrix, Taizhou, China). 1x106 cell HUDEP-2 cells resuspended in 20 μL of electroporation buffer were mixed with 1 μg PE3 plasmid and transferred to a cuvette for electroporation with the program of 380 V/30 ms. The cells were transferred to the HDUEP-2 proliferation medium immediately after electroporation and maintained in a humidified atmosphere of 5% CO_2_ at 37 °C. Forty-eight hours later, the microscope examination was performed for checking reporter gene mCherry expression. The single-cell fluorescence-activated cell sorting was then performed, and the *mCherry*^+^ cells were sorted into the 96-well plates with 150 μL proliferation medium per well and cultured in a humidified atmosphere of 5% CO_2_ at 37 °C. After 14-day incubation, the microscope examination was performed for checking reporter gene mCherry expression and colony growth. The colony was selected and transferred to the 48- or 24-well plates for further proliferation.

### 4.4. Identification of Desired Edits of Interest

When the clone cells reached 1 × 10^5^, the genome DNA extraction was performed using the Hotshot method [54]. The *HBB* gene PCR for amplification of interested beta-thal mutations was performed with the primer pair listed in Table S7 using the KOD-neo-plus (TOYOBO, Shanghai, China). Before Sanger sequencing, all PCR products were purified using the DNA Gel/PCR Purification Kit (Novoprotein, Shanghai, China). For Sanger sequencing, traces were imported to the software Snapgene Viewer for edits identification. The editing ratio calculation formula is as follows: editing (%) = (PPEs + IPEs)/total samples × 100%.

### 4.5. Off-Target Analysis

The genome DNA extraction was performed using the Hotshot method [54]. The off-target sites with three or fewer genomic mismatches and no bulges (sgRNA and nick sgRNA) of each beta-thal mutant were identified using the Cas-OFFinder tool [25]. The off-target of interests are listed in Table S3. We chose the top five off-targets and performed PCR to amplify the off-target sequences, using the corresponding primer pairs listed in Table S4 designed on the website of Primer-BLAST (https://www.ncbi.nlm.nih.gov/tools/primer-blast/index.cgi?LINK_LOC=BlastHome (accessed on 21 September 2021)). Before Sanger sequencing, all PCR products were purified using the DNA Gel/PCR Purification Kit (Novoprotein, Shanghai, China).

### 4.6. RT-PCR Assays

Cells were homogenized using TRIzol (Thermo Fisher Scientific, Waltham, MA, USA), and RNA was extracted using the RNeasy Mini Kit (Qiagen, Venlo, The Netherlands). Reverse transcription was performed using HiScript III RT SuperMix for qPCR (+gDNA wiper) (Vazyme, Nanjing, China). RT-qPCR reactions were performed using SYBR^®^ Green Realtime PCR Master Mix (TOYOBO, Shanghai, China). The Ct values for genes of interest were normalized to *GAPDH*, and expressions of genes are represented as 2^−ΔΔCt^ for fold change under control conditions. All the primers used for qPCR are listed in Table S5. The primer pairs are designed and evaluated using the webtool GETPrime [55]. The RT-PCR for sequencing the mutations in the *HBB* mRNA was performed using the KOD-neo-plus (TOYOBO, Shanghai, China). The primer pairs for RT-PCR sequencing are listed in Table S7. All PCR products were purified using the DNA Gel/PCR Purification Kit (Novoprotein, Shanghai, China).

### 4.7. Cell Apoptosis and Viability Assays

Because the cell viability of HUDEP-2 is deteriorated by extra-low or extra-high cell density [21], the HUDEP-2 was proliferated at a range from 3~8 × 10^5^ cells/mL. The cell density is maintained at approximately 3~8 × 10^5^ cells/mL for cell apoptosis analysis. Cell apoptosis was measured using the Dead Cell Apoptosis Kit with Annexin V Alexa Fluor™ 488 and Propidium Iodide (PI) (Thermo Fisher Scientific, Waltham, MA, USA) following the manufacturer’s instructions. The erythroid differentiation starts with 1 × 10^6^ cells/mL [21]. The cell viability analysis starts with 1 × 10^6^ cells/mL, both before and after erythroid differentiation, respectively. Cell viability was measured by trypan blue staining following the manufacturer’s instructions (Nexcelom, cellometer Mini, Lawrence, MA, USA).

## Figures and Tables

**Table 1:** Twenty most prevalent beta-thal alleles [9]. beta^++^, beta^+^, and beta^0^ refer to silent, moderate, and severe degrees of beta-thal, respectively.

Mutant	Distribution	Type
-88 (C>T)	African-American, Asian Indian	beta^++^
-28 (A>G)	African, Southeast Asian	beta^+^
CD1 (-G)	Mediterranean	beta^0^
CD6 (-A)	North African	beta^0^
CD14/15 (+G)	Chinese	beta^0^
CD17 (A>T)	Chinese, Japanese	beta^0^
CD19 (A>G)	Southeast Asian	beta^++^
CD26 (G>A)	Southeast Asian, European	beta^+^
CD27/28 (+C)	Chinese, Thai	beta^0^
IVS-I-1 (G>A)	North African, Italian, Greek, Balkan, West European	beta^0^
IVS-I-5 (G>C)	Asian Indian, Southeast Asian, Melanesian, Middle East	beta^0^
IVS-I-6 (T>C)	Italian, Greek, Balkan, West European (Portugal)	beta^+^
IVS-I-110 (G>A)	Italian, Greek, Cypriot, Balkan, Israeli, Lebanese, North African, West European	beta^+^
CD39 (C>T)	Italian, Greek, Balkan, North African, Israeli, West European, Middle East	beta^0^
CD41/42 (-TTCT)	Chinese, Southeast Asian, Indian	beta^++^
CD44 (-C)	Israeli, Middle East	beta^++^
CD71/72 (+A)	Chinese	beta^0^
IVS-II-1 (G>A)	Israeli, Middle East, Japanese, Turkish	beta^0^
IVS-II-654 (C>T)	Chinese, Southeast Asian, Japanese	beta^0^/beta^+^
IVS-II-745 (C>G)	Italian, Greek, Turkish	beta^+^

**Table 2:** The edits at each beta-thal mutation site, including sampling numbers and the edit number of PPE, IPE, and PEI.

Mutation	Formula	Total Samples	Type of Edits		
			PPE	IPE	PEI
-88		1		1	
-28		26			1
CD1		22		1	4
CD6		9	4		5
CD14/15		3			
CD17	F1	67		5	1
	F2	14	1	1	
CD19		7	2	2	2
CD26	F1	19		3	
	F2	2		1	
CD27/28	F1	43			
	F2	21			5
IVS-I-1		9	1	3	2
IVS-I-5		2	1	1	
IVS-I-6		3			
IVS-I-110	F1	77			
	F2	3			
CD39		11	1	1	4
CD41/42	F1	24			1
	F2	38			
CD44		5			
CD71/72		18	2	6	
IVS-II-1		2	1		
IVS-II-654	F1	12			
	F2	20			
	F3	1			
IVS-II-745		23	3	7	4

## Supplementary Materials

The following are available online at https://www.mdpi.com/article/10.3390/ijms23095002/s1.
