# Supplementary material for: Prime Editor 3 Mediated Beta-Thalassemia Mutations of the HBB Gene in Human Erythroid Progenitor Cells
Source: Int J Mol Sci. 2022 Apr 30;23(9):5002. doi: 10.3390/ijms23095002 (PMC9099916; doi:10.3390/ijms23095002)
Supplement: Supplementary file 1 [file ijms-23-05002-s001.zip › ijms-1671219-supplementary.pdf]

Supplemental data

Prime Editor 3 mediated beta-thalassemia mutations of the *HBB* gene in human erythroid progenitor cells

Haokun Zhang [1], Qinlinglan Zhou [1], Hongyan Chen [1,*], and Daru Lu [1,2,*]

[1] State Key Laboratory of Genetic Engineering, MOE Engineering Research Center of Gene Technology, School of Life Sciences, Fudan University, Shanghai 200438, China; zhanghaokun666@hotmail.com (H.Z.); 21210700056@m.fudan.edu.cn (Q.Z.)

[2] NHC Key Laboratory of Birth Defects and Reproductive Health, Chongqing Key Laboratory of Birth Defects and Reproductive Health, Chongqing Population and Family Planning, Science and Technology Research Institute, Chongqing 404100, China

* Correspondence: chenhy@fudan.edu.cn (H.C.); drlu@fudan.edu.cn (D.L.)

Supplemental figure 1, related to Figure 1

a PE vector structure (e.g.: PE vector inducing the *HBB*_CD39 mutation)

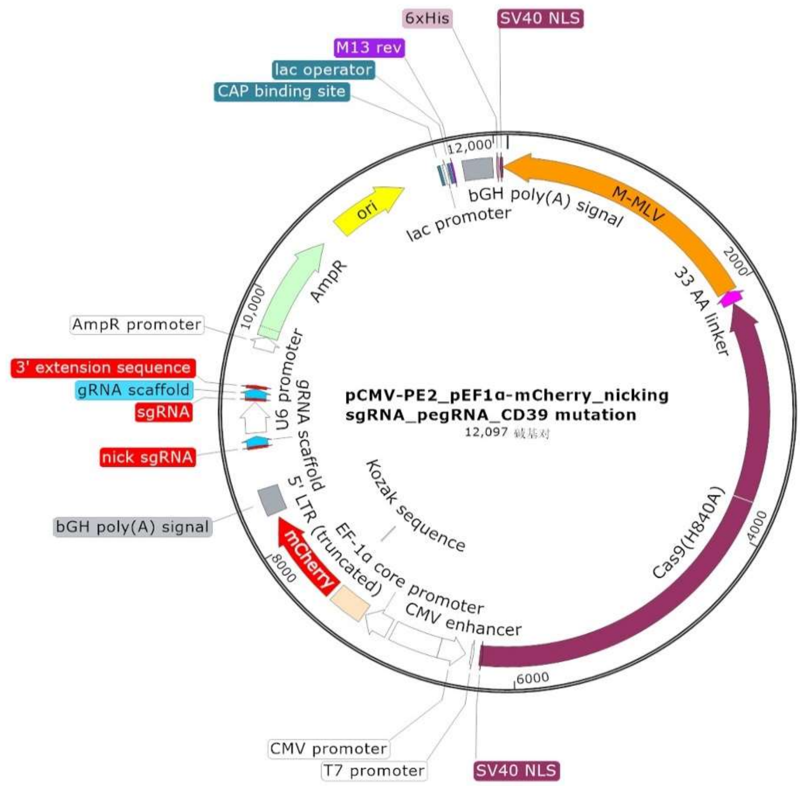

b HUDEP-2 cells with PE vector (normal *vs.* fluorescent visual fields)

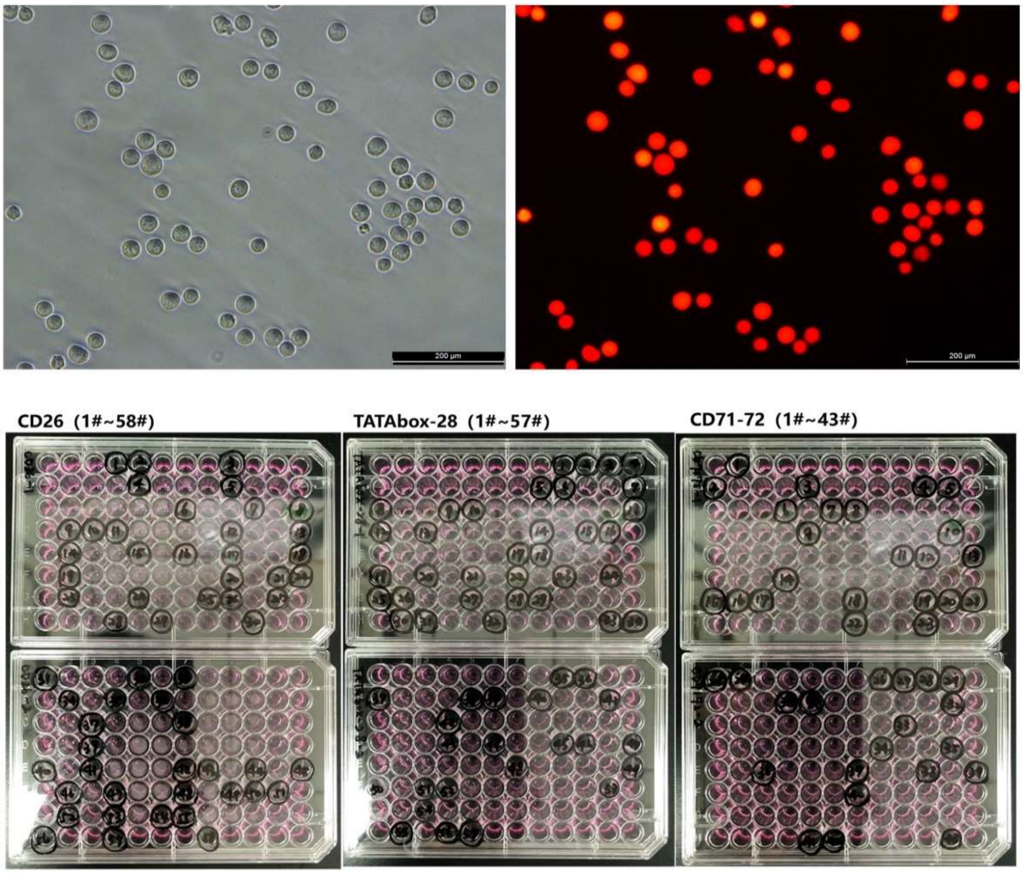

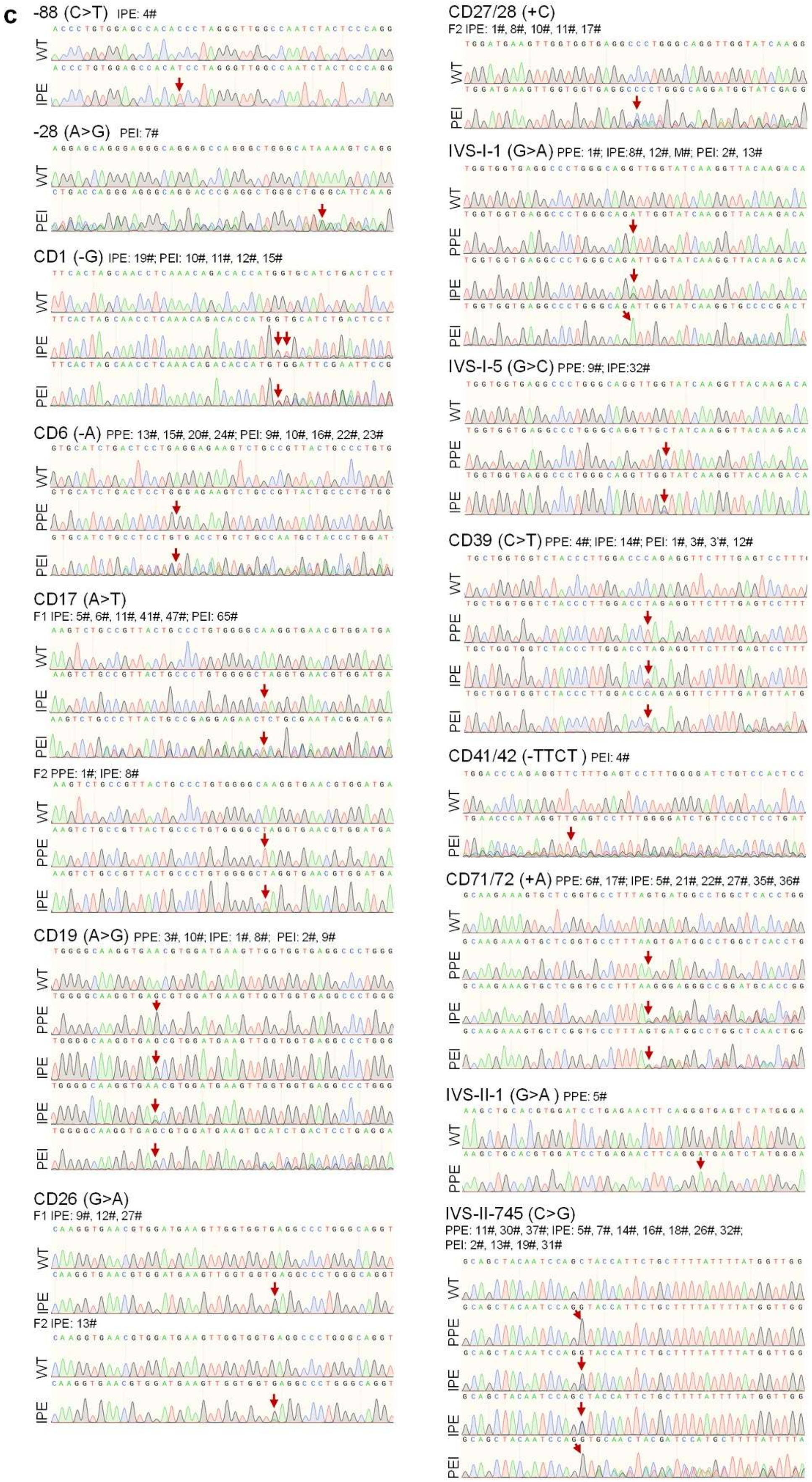
c
-88 (C>T) IPE: 4#
-28 (A>G) PEI: 7#
CD1 (-G) IPE: 19#; PEI: 10#, 11#, 12#, 15#
CD6 (-A) PPE: 13#, 15#, 20#, 24#; PEI: 9#, 10#, 16#, 22#, 23#
CD17 (A>T)
F1 IPE: 5#, 6#, 11#, 41#, 47#; PEI: 65#
F2 PPE: 1#; IPE: 8#
CD19 (A>G) PPE: 3#, 10#; IPE: 1#, 8#; PEI: 2#, 9#
CD26 (G>A)
F1 IPE: 9#, 12#, 27#
F2 IPE: 13#
CD27/28 (+C)
F2 IPE: 1#, 8#, 10#, 11#, 17#
IVS-I-1 (G>A) PPE: 1#; IPE:8#, 12#, M#; PEI: 2#, 13#
IVS-I-5 (G>C) PPE: 9#; IPE:32#
CD39 (C>T) PPE: 4#; IPE: 14#; PEI: 1#, 3#, 3'#, 12#
CD41/42 (-TTCT) PEI: 4#
CD71/72 (+A) PPE: 6#, 17#; IPE: 5#, 21#, 22#, 27#, 35#, 36#
IVS-II-1 (G>A) PPE: 5#
IVS-II-745 (C>G)
PPE: 11#, 30#, 37#; IPE: 5#, 7#, 14#, 16#, 18#, 26#, 32#;
PEI: 2#, 13#, 19#, 31#
WT
IPE
PPE
PEI

(a) The structure of the PE3 plasmid used in this study. The plasmid contains a nCas9 fused with the engineered M-MLV reverse-transcriptase, reporter gene *mCherry*, Nick sgRNA, and pegRNA. (b) Microscope observation of the HUDEP-2 cells containing the PE3 plasmid. Approximately twenty-four hours after electroporation, the cells express mCherry protein and show bright red fluorescence in the fluorescence field. Scale bar: 200 μm. (c) Sequencing traces of beta-thal clones. Red arrow indicates on-target edits.

Supplemental figure 2, related to Figure 3

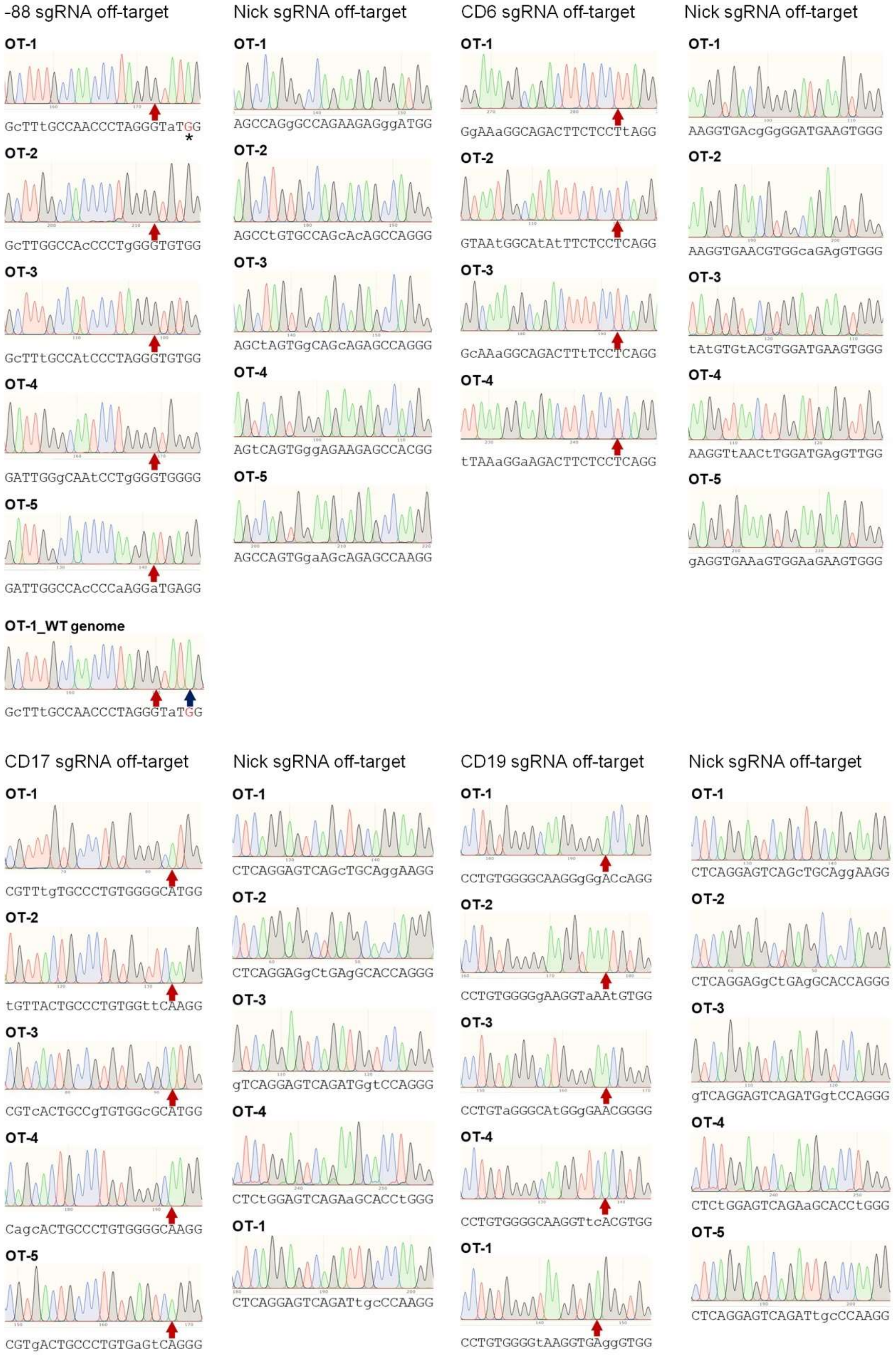

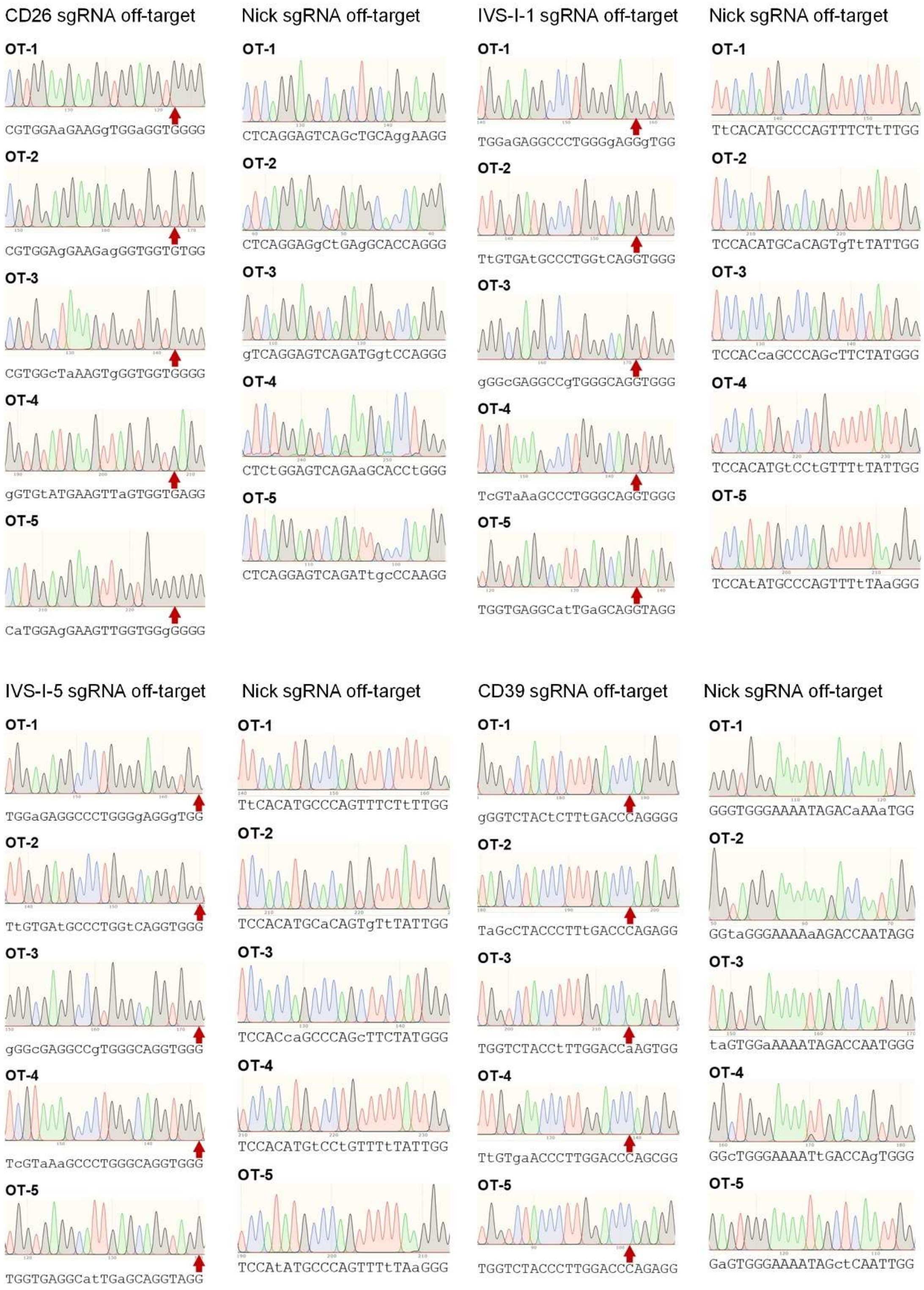
CD26 sgRNA off-target
OT-1
CGTGGAaGAAGgTGGaGGTGGGG
OT-2
CGTGGAgGAAGagGGTGGTGTGG
OT-3
CGTGGcTaAAGTgGGTGGTGGGG
OT-4
gGTGtATGAAGTTaGTGGTGAGG
OT-5
CaTGGAgGAAGTTGGTGGgGGGG
Nick sgRNA off-target
OT-1
CTCAGGAGTCAGcTGCAggAAGG
OT-2
CTCAGGAGgCtGAgGCACCAGGG
OT-3
gTCAGGAGTCAGATGgtCCAGGG
OT-4
CTCtGGAGTCAGAaGCACCtGGG
OT-5
CTCAGGAGTCAGATtgcCCAAGG
IVS-I-1 sgRNA off-target
OT-1
TGGaGAGGCCCTGGGgAGGgTGG
OT-2
TtGTGAtGCCCTGGtCAGGTGGG
OT-3
gGGcGAGGCCgTGGGCAGGTGGG
OT-4
TcGTaAaGCCCTGGGCAGGTGGG
OT-5
TGGTGAGGCatTGaGCAGGTAGG
Nick sgRNA off-target
OT-1
TtCACATGCCCAGTTTCTtTTGG
OT-2
TCCACATGCaCAGTgTtTATTGG
OT-3
TCCACcaGCCCAGcTTCTATGGG
OT-4
TCCACATGtCCtGTTTtTATTGG
OT-5
TCCAtATGCCCAGTTTtTAaGGG
IVS-I-5 sgRNA off-target
OT-1
TGGaGAGGCCCTGGGgAGGgTGG
OT-2
TtGTGAtGCCCTGGtCAGGTGGG
OT-3
gGGcGAGGCCgTGGGCAGGTGGG
OT-4
TcGTaAaGCCCTGGGCAGGTGGG
OT-5
TGGTGAGGCatTGaGCAGGTAGG
Nick sgRNA off-target
OT-1
TtCACATGCCCAGTTTCTtTTGG
OT-2
TCCACATGCaCAGTgTtTATTGG
OT-3
TCCACcaGCCCAGcTTCTATGGG
OT-4
TCCACATGtCCtGTTTtTATTGG
OT-5
TCCAtATGCCCAGTTTtTAaGGG
CD39 sgRNA off-target
OT-1
gGGTCTACtCTTtGACCCAGGGG
OT-2
TaGcCTACCCTTtGACCCAGAGG
OT-3
TGGTCTACCtTTGGACCaAGTGG
OT-4
TtGTgaACCCTTGGACCCAGCGG
OT-5
TGGTCTACCCTTGGACCCAGAGG
Nick sgRNA off-target
OT-1
GGGTGGGAAAATAGACaAAaTGG
OT-2
GGtaGGGAAAAaAGACCAATAGG
OT-3
taGTGGaAAAATAGACCAATGGG
OT-4
GGcTGGGAAAATtGACCAgTGGG
OT-5
GaGTGGGAAAATAGctCAATTGG

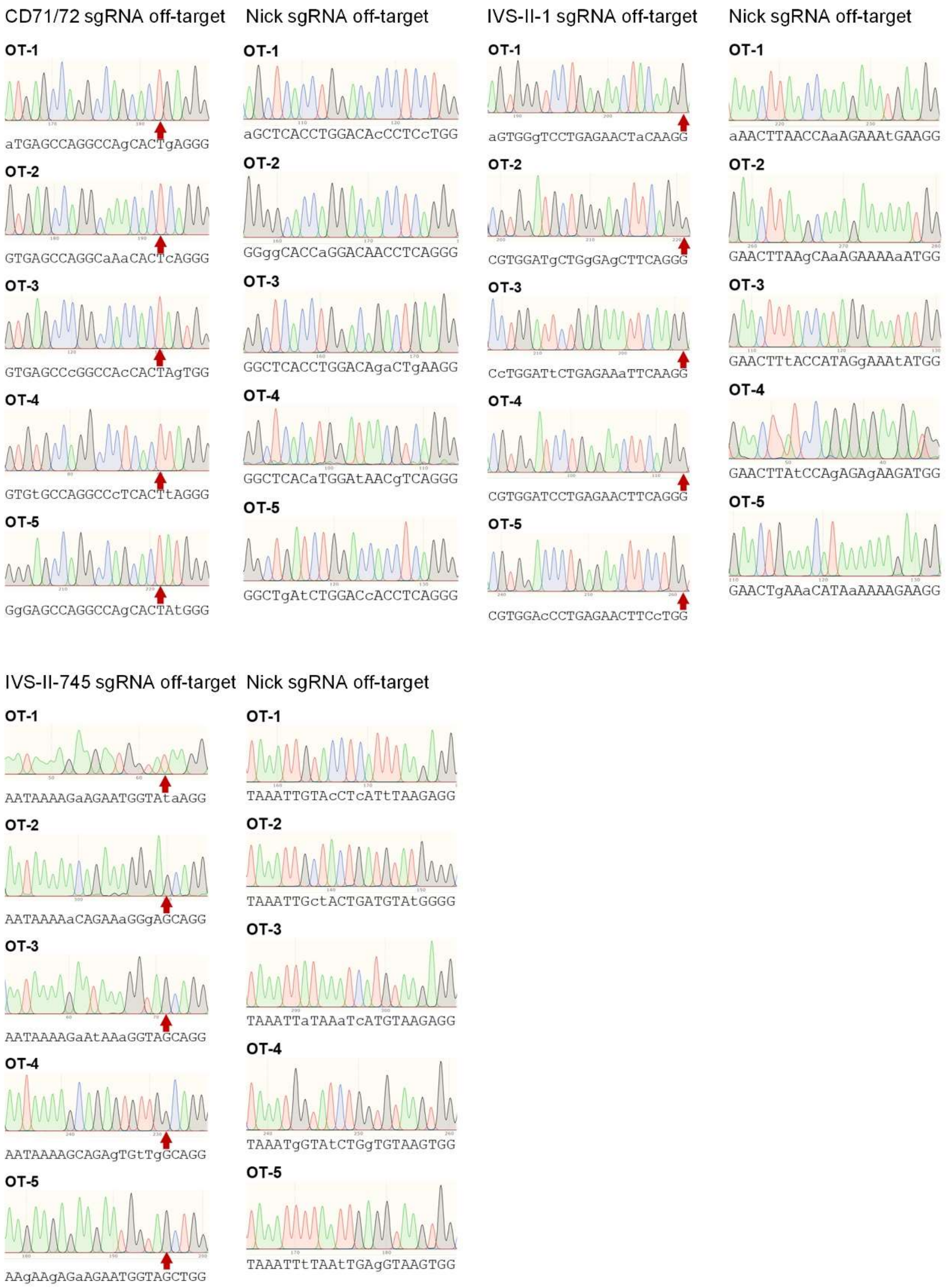

Using the webtool Cas-offinder[25], off-targets were identified with at least three mismatches compared with the on-target sequences. Red arrow indicates the editing position of pegRNA. Asterisk and blue arrow illustrate the single nucleotide polymorphism site in the OT-1 in WT and -88 cells, respectively. Lowercase letters indicate mismatches.

Supplemental figure 3, related to Figure 4

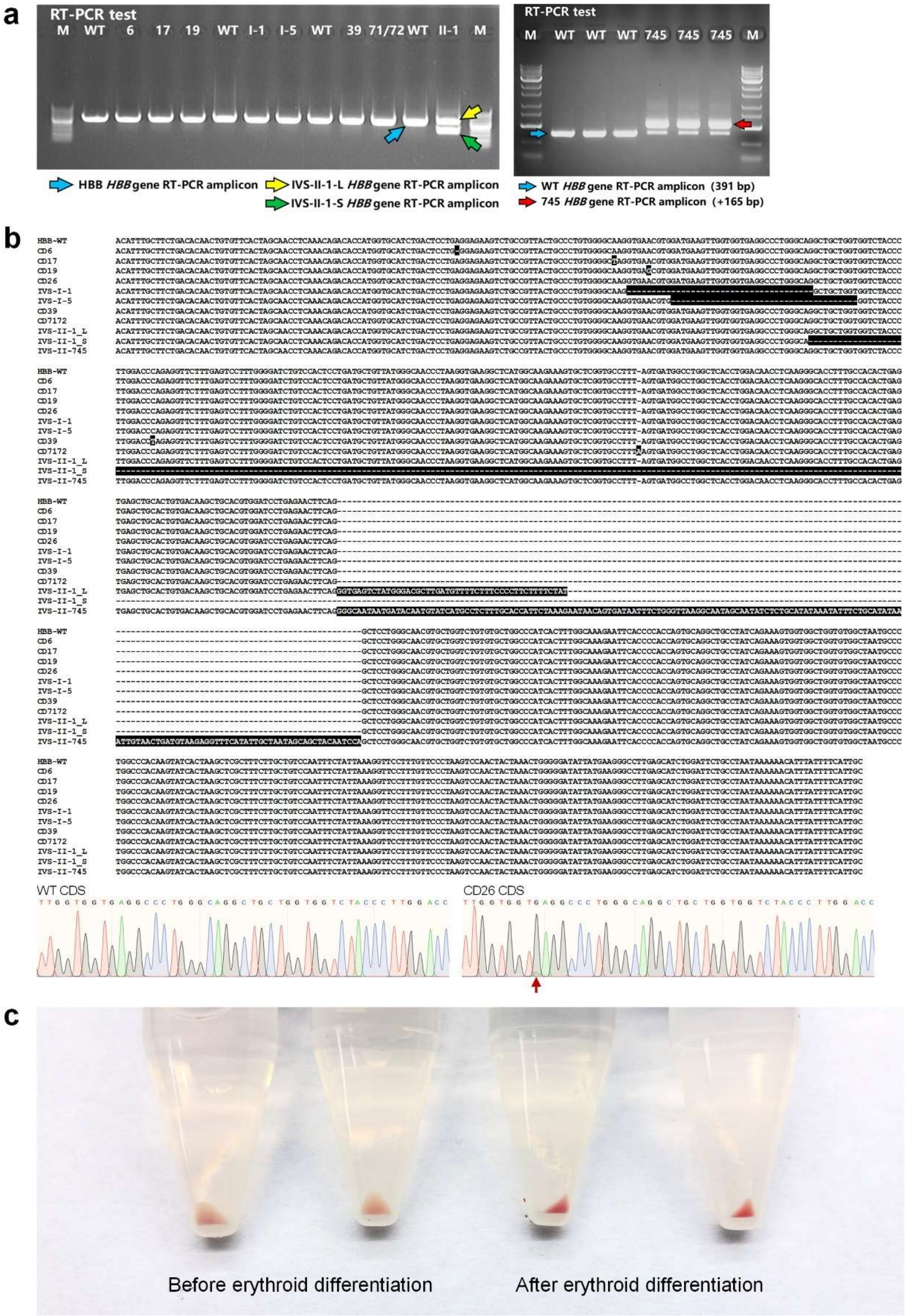

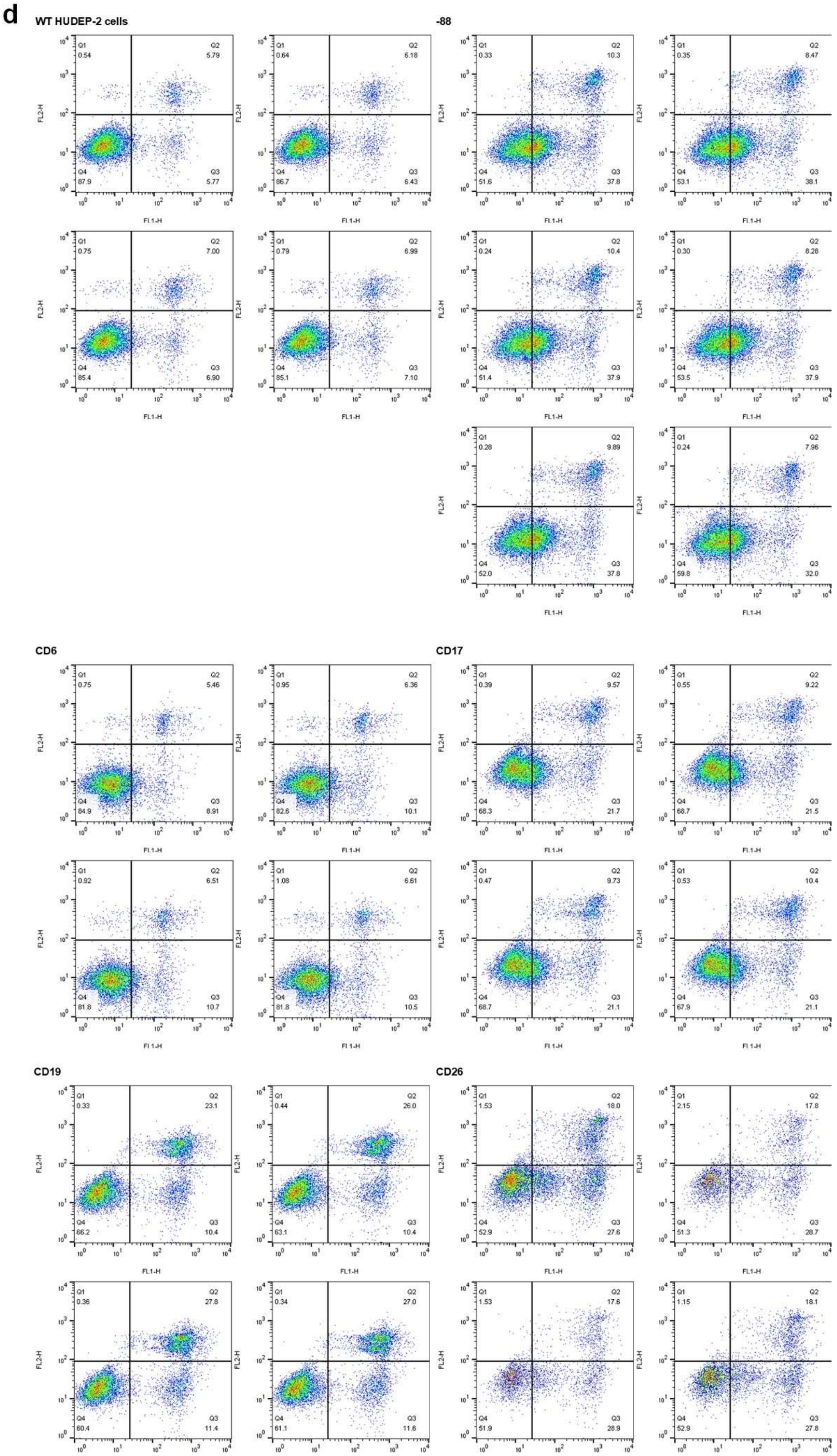
d
WT HUDEP-2 cells
-88
CD6
CD17
CD19
CD26
FL1-H
FL2-H

continuous d

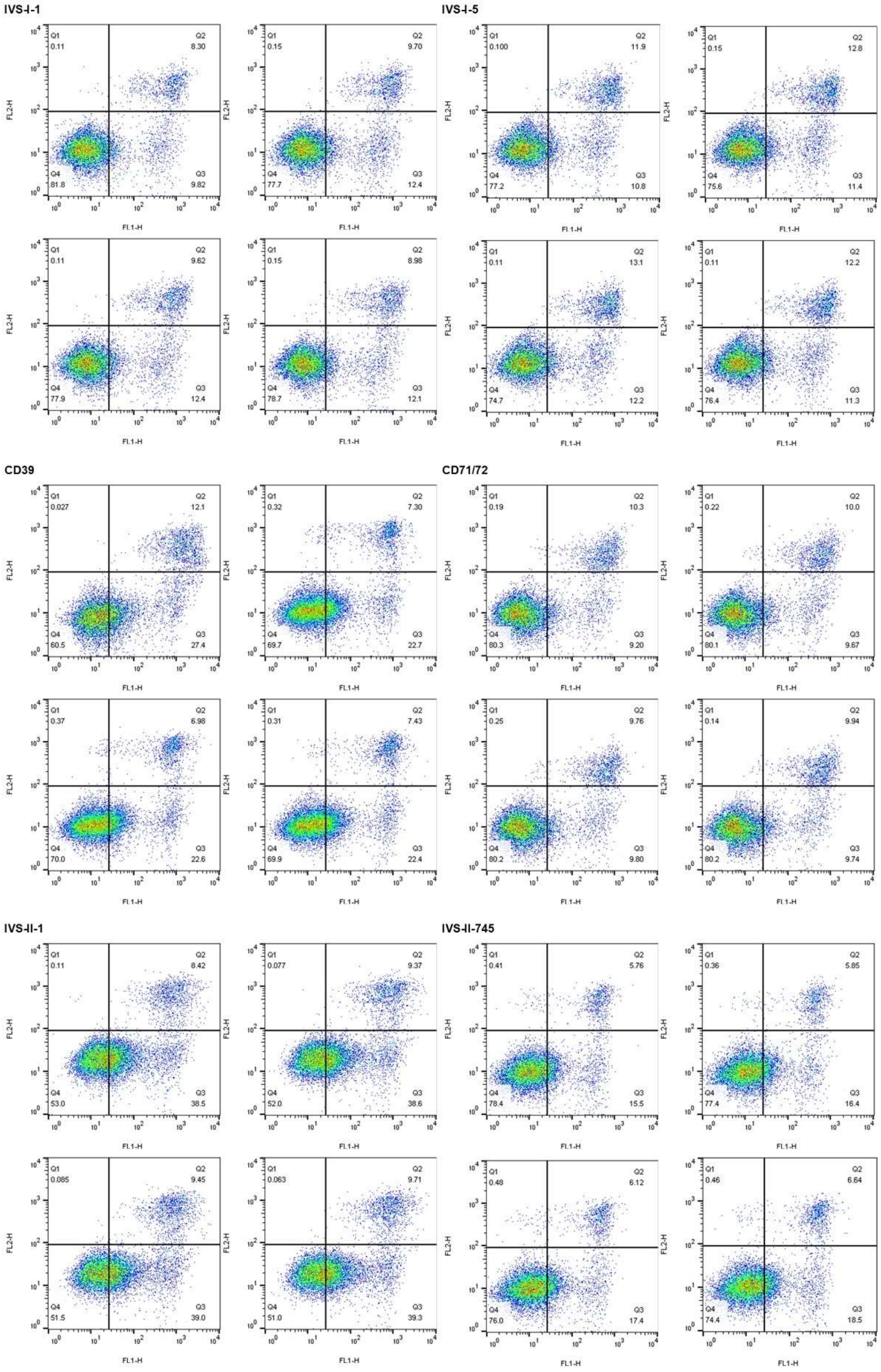

e

	Viable cells (%)						Apoptotic cells (%)						Necrotic cells (%)					
WT	87.9	86.7	85.4	85.1			5.77	6.43	6.9	7.1			5.79	6.18	7	6.99		
-88	5.16	53.1	51.4	53.5	52	59.8	37.8	38.1	37.9	37.9	37.8	32	10.3	8.47	10.4	8.28	9.89	7.96
CD6	84.9	82.6	81.8	81.8			8.91	10.1	10.7	10.5			5.46	6.36	6.51	6.61		
CD17	68.3	68.7	68.7	67.9			21.7	21.5	21.1	21.1			9.57	9.22	9.73	10.4		
CD19	66.2	63.1	60.4	61.1			10.4	10.4	11.4	11.6			23.1	26	27.8	27		
CD26	52.9	51.3	51.9	52.9			27.6	28.7	28.9	27.8			18	17.8	17.6	18.1		
IVS-I-1	81.8	77.7	77.9	78.7			9.82	12.4	12.4	12.1			8.3	9.7	9.62	8.98		
IVS-I-5	77.2	75.6	74.7	76.4			10.8	11.4	12.2	11.3			11.9	12.8	13.1	12.2		
CD39	60.5	69.7	70	69.9			27.4	22.7	22.6	22.4			12.1	7.3	6.98	7.43		
CD71/72	80.3	80.1	80.2	80.2			9.2	9.67	9.8	9.74			10.3	10	9.76	9.94		
IVS-II-1	53	52	51.5	51			38.5	38.6	39	39.3			8.42	9.37	9.45	9.71		
IVS-II-745	78.4	77.4	76	74.4			15.5	16.4	17.4	18.5			5.76	5.85	6.12	6.64		

f Cell observation during erythroid differentiation

WT HUDEP-2 cells

β-thalassemic mutant

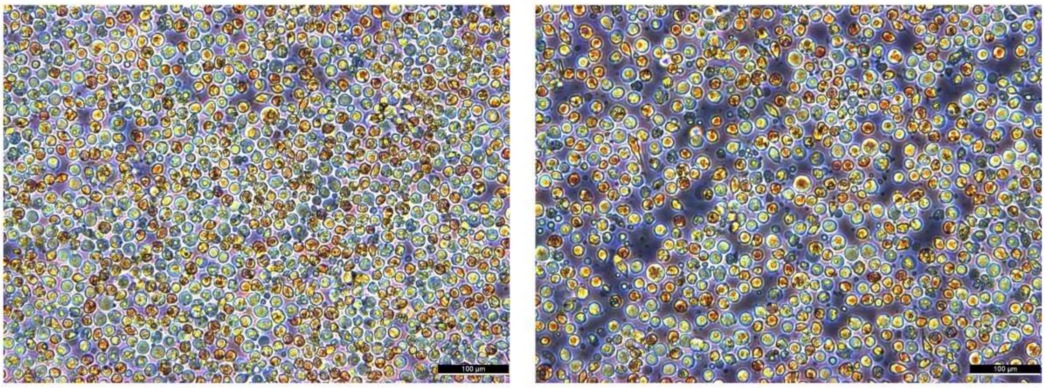

g Sampling chips

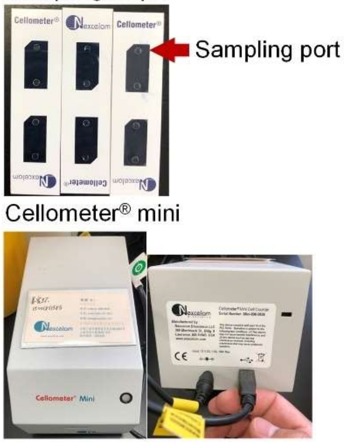

Sample	Before erythroid differentiation			After erythroid differentiation		
	Repeat 1	Repeat 2	Repeat 3	Repeat 1	Repeat 2	Repeat 3
WT	0.825	0.844	0.876	0.687	0.71	0.698
-88	0.575	0.677	0.66	0.483	0.49	0.521
CD6	0.489	0.505	0.495	0.395	0.381	0.441
CD17	0.618	0.671	0.655	0.554	0.602	0.659
CD19	0.606	0.606	0.597	0.545	0.538	0.566
CD26	0.463	0.456	0.452	0.426	0.393	0.457
IVS-I-1	0.798	0.762	0.782	0.492	0.407	0.411
IVS-I-5	0.764	0.748	0.673	0.582	0.542	0.532
CD39	0.608	0.617	0.629	0.423	0.579	0.491
CD71/72	0.762	0.766	0.815	0.617	0.621	0.648
IVS-II-1	0.749	0.68	0.712	0.548	0.581	0.537
IVS-II-745	0.677	0.709	0.655	0.451	0.461	0.434

(a) Electrophoresis results of RT-PCR amplicons. 6, 17, 19, I-1, I-5, 39, 71/72, II-1, and 745 mean CD6, CD17, CD19, IVS-I-1, IVS-I-5, CD39, CD71/72, and IVS-II-745, respectively. (b) Alignment of RT-PCR sequencing results and sequencing traces of WT and CD26 HBB cDNA. White word with a black background indicates the mutation site. Red arrow indicates the detectable adenine signal in the *HBB* transcript. (c) Observation of HUDEP-2 cells before and after erythroid differentiation. (d) Flow cytometry results for apoptosis analysis. Values displayed in the lower left, lower right, and upper right quadrants are the proportion of viable, apoptotic, and necrotic cells, respectively. (e) Row data of apoptosis analysis. (f) Microscopic examination of WT HUDEP-2 and beta-thal cells during erythropoiesis. More necrotic cells and debris are observed in beta-thal cells. Scale bar: 100 μm. (g) Sampling chip and Cellometer for cell viability analysis. Red arrow indicates the sampling port. Row data of cell viability (%) are shown in the table.

Supplemental figure 4

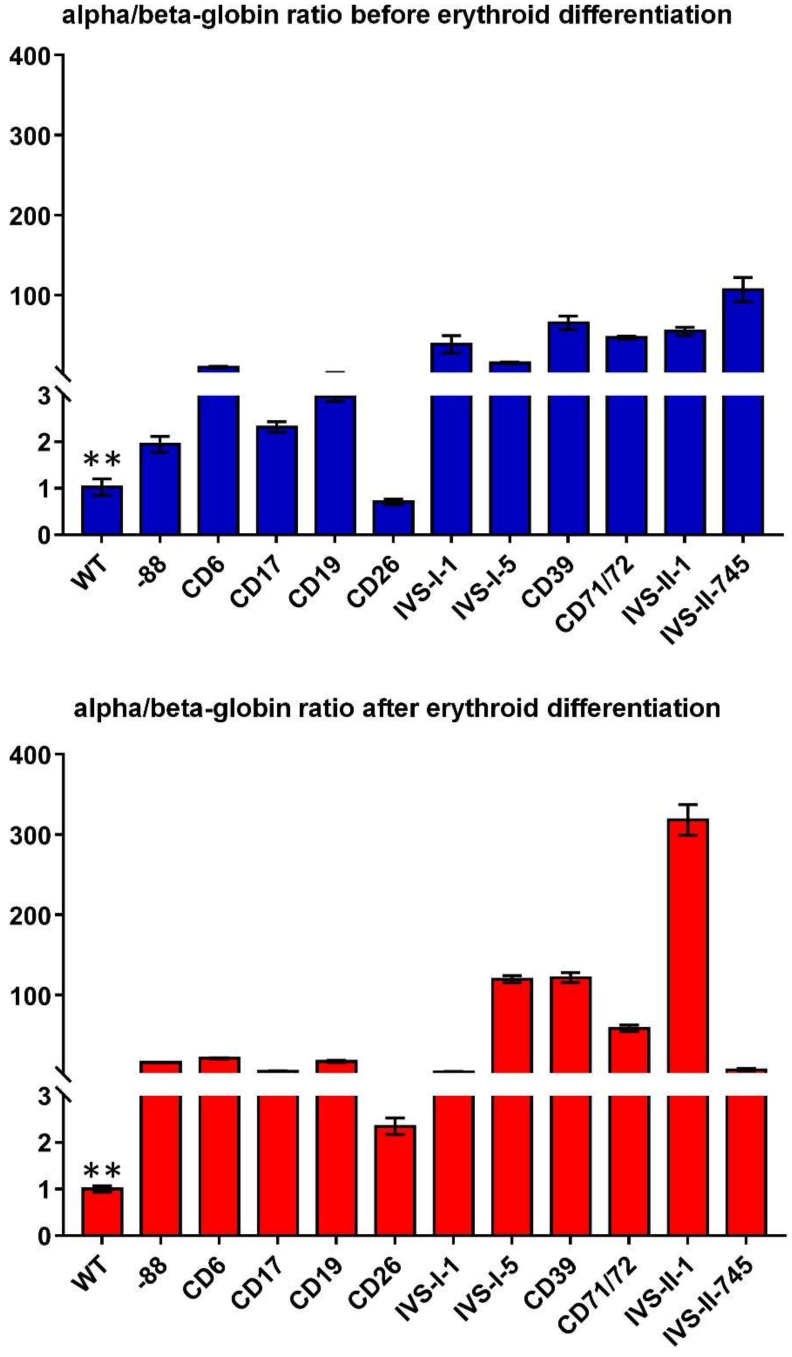

Alpha/beta-globin mRNA ratios of WT HUDEP-2 and beta-thal cells, before and after erythroid differentiation, respectively. All gene expression values were normalized to the *GAPDH* gene (Table S6). *x*-axis shows WT HUDEP-2 and different beta-thal cells, and *y*-axis shows the alpha/beta-globin mRNA ratio (mean ± SEM value). ** means the *P*-value ≤ 0.01 (*t*-test).

Supplemental Table 1: Formulas of pegRNAs and nick sgRNAs for inducing beta-thalassemic mutations in this study

Mutant	Spacer sequence	3' extension sequence (PBS + RT-template)	Nick sgRNA	Note
-88	GATTGGCCAACCCTAGGGTG	GTGGAGCCACATCCTAGGGTTGGC	AGCCAGTGCCAGAAGAGCCA	
-28	CCAATCTACTCCCAGGAGCA	CCTGACTTTCATGCCCAGCCCTGGCTCCTGCCCTCCCTGCTCCTGGGAGTAGA	ACCCTAGGGTGTGGCTCCAC	
CD1	CTCAGGAGTCAGATGCACCA	GCAACCTCAAACAGACACCATGTGCATCTGACTCCT	AGGGCTGGGCATAAAAGTCA	
CD6	GTAACGGCAGACTTCTCCTC	ATCTGACTCCTGGGAGAAGTCTGCCG	AAGGTGAACGTGGATGAAGT	
CD14/15	GTCTGCCGTTACTGCCCTGT	TCACCTTGCCCCACCAGGGCAGTAACG	CTCAGGAGTCAGATGCACCA	
CD17	TCTGCCGTTACTGCCCTGTG	GTTCACCTAGCCCCACAGGGCAGTAACGG	CTCAGGAGTCAGATGCACCA	formula 1
	CGTTACTGCCCTGTGGGGCA	ACGTTCACCTAGCCCCACAGGGCA	CTCAGGAGTCAGATGCACCA	formula 2
CD19	CCTGTGGGGCAAGGTGAACG	ACTTCATCCACGCTCACCTTGCCC	CTCAGGAGTCAGATGCACCA	
CD26	AAGGTGAACGTGGATGAAGT	GCCCAGGGCCTTACCACCAACTTCATCCACGTTCA	CTTGCCCCACAGGGCAGTAA	formula 1
	CGTGGATGAAGTTGGTGGTG	GCCCAGGGCCTTACCACCAACTTCATC	CTCAGGAGTCAGATGCACCA	formula 2
CD27/28	AAGGTGAACGTGGATGAAGT	CCTGCCCAGGGGCCTCACCACCAACTTCATCCACGTTCA	CTTGCCCCACAGGGCAGTAA	formula 1
	GAAGTTGGTGGTGAGGCCCT	GATACCAACCTGCCCAGGGGCCTCACCACCAAC	CTCAGGAGTCAGATGCACCA	formula 2
IVS-I-1	TGGTGAGGCCCTGGGCAGGT	ACCTTGATACCAATCTGCCCAGGGC	TCCACATGCCCAGTTTCTAT	
IVS-I-5	TGGTGAGGCCCTGGGCAGGT	ACCTTGATAGCAACCTGCCCAGGGC	TCCACATGCCCAGTTTCTAT	
IVS-I-6	TGGTGAGGCCCTGGGCAGGT	ACCTTGATGCCAACCTGCCCAGGGC	TCCACATGCCCAGTTTCTAT	
IVS-I-110	GACCACCAGCAGCCTAAGGG	CTCTCTGCCTATTAGTCTATTTTCCCACCCTTAGGCTGCTGGT	TGGTCTACCCTTGGACCCAG	formula 1
	CACTGACTCTCTCTGCCTAT	AAAATAGACTAATAGGCAGAGAGAGTCA	GTAGACCACCAGCAGCCTAA	formula 2
CD39	TGGTCTACCCTTGGACCCAG	TCAAAGAACCTCTAGGTCCAAGGGTA	GGGTGGGAAAATAGACCAAT	
CD41/42	TGGTCTACCCTTGGACCCAG	GGACTCAACCTCTGGGTCCAAGGGTAG	GGGTGGGAAAATAGACCAAT	formula 1
	AGGAGTGGACAGATCCCCAA	GGACCCAGAGGTTGAGTCCTTTGGGGATCTGTCCAC	GTGCTCGGTGCCTTTAGTGA	formula 2
CD44	AGGAGTGGACAGATCCCCAA	TCTTTGAGTCTTTGGGGATCTGTCCAC	GTGCTCGGTGCCTTTAGTGA	
CD71/72	GTGAGCCAGGCCATCACTAA	GTGCTCGGTGCCTTTAAGTGATGGCCTGGC	GGCTCACCTGGACAACCTCA	
IVS-II-1	CGTGGATCCTGAGAACTTCA	AGACTCATCCTGAAGTTCTCAGGATCC	GAACTTAACCATAGAAAAGA	
IVS-II-654	CAGTGATAATTTCTGGGTTA	TTGCTATTACCTTAACCCAGAAATTATC	AATAAAAGCAGAATGGTAGC	formula 1
	CAGTGATAATTTCTGGGTTA	GCAGAGATATTGCTATTACCTTAACCCAGAAATTATC	GCCCTGAAAGAAAGAGATTA	formula 2
	CAGTGATAATTTCTGGGTTA	TGCTATTACCTTAACCCAGAAATTATCACTG	GCCCTGAAAGAAAGAGATTA	formula 3
IVS-II-745	AATAAAAGCAGAATGGTAGC	CAATCCAGGTACCATTCTGCTTT	TAAATTGTAACTGATGTAAG	

Supplemental Table 2: The information of pegRNAs and nick sgRNAs inducing beta-thalassemia mutations in this study

Mutation	Formula	Editing %	Editing position (nt)	Nicking position (nt)	Template length (nt)	PBS length (nt)	pegRNA folding, kcal/mol		GC %			Tm (ºC)		
							Initial ΔG	ΔΔG	sgRNA	3’ extension	Nick sgRNA	sgRNA	3’ extension	Nick sgRNA
-88		100.00	1	52	12	12	-55.30~-30.40	24.9	60	62.5	60	67.46	74	69.65
-28		0.00	31	-37	40	13	-65.50~-34.90	30.6	55	64.15	65	64.08	74.88	67.4
CD1		4.55	1	78	22	14	-53.50~-30.00	23.5	55	50	50	64.22	80.77	65.53
CD6		44.44	2	-47	13	13	-53.20~-30.40	22.8	55	57.69	45	60.42	73.92	61
CD14/15		0.00	2	-39	16	11	-48.30~-30.20	18.1	60	55	55	66.4	64.86	64.22
CD17	F1	7.46	8	-40	16	13	-61.80~-30.30	31.5	60	62.07	55	68.5	78.51	64.22
	F2	14.29	3	-45	13	11	-52.00~-31.50	20.5	65	62.5	55	72.42	76.62	64.22
CD19		57.14	1	-54	11	13	-42.80~-21.20	21.6	65	58.33	55	72.05	74.73	64.22
CD26	F1	15.79	11	-31	22	13	-54.40~-24.00	30.4	45	57.14	60	61	84.45	69.27
	F2	50.00	3	-72	14	13	-51.30~-21.50	29.8	55	59.26	55	66.45	76.88	64.22
CD27/28	F1	0.00	16	-31	26	13	-54.40~-25.50	28.9	45	61.54	60	61	69.75	69.27
	F2	0.00	1	-79	19	14	-61.30~-27.80	33.5	60	63.64	55	67.51	84.44	64.22
IVS-I-1		44.44	2	42	15	10	-49.50~-28.30	21.2	70	56	45	75.45	73.85	61.9
IVS-I-5		100.00	6	42	15	10	-55.10~-31.00	24.1	70	60	45	75.45	75.61	61.9
IVS-I-6		0.00	7	42	15	10	-53.60~-28.90	24.7	70	64	45	75.45	79.81	61.9
IVS-I-110	F1	0.00	17	-29	30	13	-63.10~-30.30	32.8	65	48.84	60	68.01	65.97	66.11
	F2	0.00	5	24	14	14	-55.30~-24.70	30.6	50	35.71	55	56.01	61.66	61.82
CD39		18.18	1	-46	14	12	-46.60~-25.70	20.9	60	46.15	45	66.11	67.44	61.33
CD41/42	F1	0.00	7	-46	14	13	-59.00~-33.50	25.5	60	59.26	45	66.11	73.52	61.33
	F2	0.00	13	-79	23	13	-56.90~-36.00	20.9	55	58.33	55	65.8	82.87	65.24
CD44		0.00	5	-79	14	13	-53.20~-24.80	28.4	55	48.15	55	65.8	71.6	65.24
CD71/72		44.44	1	-27	17	13	-57.60~-25.50	32.1	55	60	60	65.12	81.15	67.67
IVS-II-1		50.00	6	44	13	14	-53.90~-26.00	27.9	50	48.15	30	62.86	68.55	50.08
IVS-II-654	F1	0.00	7	99	15	13	-43.10~-26.00	17.1	35	32.14	35	54.1	63.02	55.15
	F2	0.00	7	-82	24	13	-46.30~-20.80	25.5	35	35.14	40	54.1	58.09	57.53
	F3	0.00	7	-82	14	17	-52.00~-28.90	23.1	35	35.48	40	54.1	66.93	57.53
IVS-II-745		43.48	2	39	10	13	-44.00~-23.30	20.7	35	43.48	25	55.15	64.91	46.63

Supplemental Table 3: Search results of Cas-OFFinder for off-target examination in this study

-88 sgRNA	crRNA	DNA	Chromosome	Position	Direction	Mismatches	Bulge Size
No.1	GATTGGCCAACCCTAGGGTGNGG	GcTTtGCCAACCCTAGGGTaTGG	chr15	44910699	+	3	0
No.2	GATTGGCCAACCCTAGGGTGNGG	GcTTGGCCAcCCCTgGGGTGTGG	chr20	61796608	+	3	0
No.3	GATTGGCCAACCCTAGGGTGNGG	GcTTtGCCAtCCCTAGGGTGTGG	chr1	63259619	-	3	0
No.4	GATTGGCCAACCCTAGGGTGNGG	GATTGGgCAAtCCTgGGGTGGGG	chr1	88388885	+	3	0
No.5	GATTGGCCAACCCTAGGGTGNGG	GATTGGCCAcCCCaAGGaTGAGG	chr4	17714017	-	3	0
No.6	GATTGGCCAACCCTAGGGTGNGG	ttTTGGCCAACCCTgGGGTGGGG	chrX	17687669	-	3	0
No.7	GATTGGCCAACCCTAGGGTGNGG	GATTGGCCAACCCTAGGGTGTGG	chr11	5227141	+	0	0
No.8	GATTGGCCAACCCTAGGGTGNGG	GATTGGCCAACaaTAGGGaGTGG	chr11	96034018	-	3	0
No.9	GATTGGCCAACCCTAGGGTGNGG	cAgTGGaCAACCCTAGGGTGGGG	chr11	119312998	-	3	0
No.10	GATTGGCCAACCCTAGGGTGNGG	cATcGGCCAAtCCTAGGGTGGGG	chr10	101589028	-	3	0
No.11	GATTGGCCAACCCTAGGGTGNGG	GAcTGGCCAACCtcAGGGTGGGG	chr19	16883771	-	3	0
-88 nick sgRNA	crRNA	DNA	Chromosome	Position	Direction	Mismatches	Bulge Size
No.1	AGCCAGTGCCAGAAGAGCCANGG	AGCCAGgGCCAGAAGAGggATGG	chr8	6850407	+	3	0
No.2	AGCCAGTGCCAGAAGAGCCANGG	AGCCtGTGCCAGcAcAGCCAGGG	chr8	143515883	-	3	0
No.3	AGCCAGTGCCAGAAGAGCCANGG	AGCtAGTGgCAGcAGAGCCAGGG	chr15	77654706	-	3	0
No.4	AGCCAGTGCCAGAAGAGCCANGG	AGtCAGTGggAGAAGAGCCACGG	chr1	25614534	+	3	0
No.5	AGCCAGTGCCAGAAGAGCCANGG	AGCCAGTGgaAGcAGAGCCAAGG	chr1	83994288	-	3	0
No.6	AGCCAGTGCCAGAAGAGCCANGG	AGaCAGTGCCAGAAGAGaCtTGG	chr1	236523416	-	3	0
No.7	AGCCAGTGCCAGAAGAGCCANGG	AGCCAGTtCCtGAAcAGCCACGG	chr7	130153537	+	3	0
No.8	AGCCAGTGCCAGAAGAGCCANGG	AGCaAtTGCCAGtAGAGCCATGG	chr2	10136036	-	3	0
No.9	AGCCAGTGCCAGAAGAGCCANGG	tGCCAGaGgCAGAAGAGCCAGGG	chr2	46460323	+	3	0
No.10	AGCCAGTGCCAGAAGAGCCANGG	AGgCAGTGCCAGAgGAGaCAGGG	chr2	46963181	-	3	0
No.11	AGCCAGTGCCAGAAGAGCCANGG	AGgCAGTGgCAGAAGAGCaAAGG	chr2	54927873	-	3	0
No.12	AGCCAGTGCCAGAAGAGCCANGG	AcCCtGTGCCAGAAGAGCCAGGG	chr2	109062964	+	2	0
No.13	AGCCAGTGCCAGAAGAGCCANGG	AGCaAGTGCCcGcAGAGCCAGGG	chr2	120346945	-	3	0
No.14	AGCCAGTGCCAGAAGAGCCANGG	AGCCtGTGaCAGAAGAGCtACGG	chr12	13912172	-	3	0
No.15	AGCCAGTGCCAGAAGAGCCANGG	gcCCAGTGCCAGAAGAGaCATGG	chr12	124399310	+	3	0
No.16	AGCCAGTGCCAGAAGAGCCANGG	AGCCAGgGCCAGAgGAGCCgAGG	chr9	96074155	-	3	0
No.17	AGCCAGTGCCAGAAGAGCCANGG	AGCCtGTGtCAGcAGAGCCAAGG	chrX	132875545	+	3	0
No.18	AGCCAGTGCCAGAAGAGCCANGG	tGtCAGTGCCAGcAGAGCCAGGG	chr14	58152286	-	3	0
No.19	AGCCAGTGCCAGAAGAGCCANGG	AcCCAGTtCCAGAAaAGCCAGGG	chr14	105586251	-	3	0

No.20	AGCCAGTGCCAGAAGAGCCANGG	AcCCAGTtCCAGAAaAGCCAGGG	chr14	105706482	-	3	0
No.21	AGCCAGTGCCAGAAGAGCCANGG	AGCCAcTtCCtGAAGAGCCAGGG	chr6	66382411	+	3	0
No.22	AGCCAGTGCCAGAAGAGCCANGG	AGCCAGTGgCAGAAGAGggATGG	chr6	168374299	-	3	0
No.23	AGCCAGTGCCAGAAGAGCCANGG	AGCCAGTctCAGAAGAGCCcAGG	chr11	2094618	-	3	0
No.24	AGCCAGTGCCAGAAGAGCCANGG	AGCCAGTGCCAGAAGAGCCAAGG	chr11	5227204	-	0	0
No.25	AGCCAGTGCCAGAAGAGCCANGG	AGCCAaaGCCtGAAGAGCCAAGG	chr13	24996610	+	3	0
No.26	AGCCAGTGCCAGAAGAGCCANGG	AGgCAGTGagAGAAGAGCCAGGG	chr13	98224738	+	3	0
CD6 sgRNA	crRNA	DNA	Chromosome	Position	Direction	Mismatches	Bulge Size
No.1	GTAACGGCAGACTTCTCCTCNGG	GgAAaGGCAGACTTCTCCTtAGG	chr5	56751342	-	3	0
No.2	GTAACGGCAGACTTCTCCTCNGG	GTAAtGGCAtAtTTCTCCTCAGG	chr1	227706689	+	3	0
No.3	GTAACGGCAGACTTCTCCTCNGG	GcAAaGGCAGACTTtTCCTCAGG	chr6	11370393	-	3	0
No.4	GTAACGGCAGACTTCTCCTCNGG	GTAACGGCAGACTTCTCCTCAGG	chr11	5226983	+	0	0
No.5	GTAACGGCAGACTTCTCCTCNGG	tTAAaGGaAGACTTCTCCTCAGG	chr3	182066119	-	3	0
CD6 nick sgRNA	crRNA	DNA	Chromosome	Position	Direction	Mismatches	Bulge Size
No.1	AAGGTGAACGTGGATGAAGTNGG	AAGGTGAcgGgGGATGAAGTGGG	chr1	36122669	-	3	0
No.2	AAGGTGAACGTGGATGAAGTNGG	AAGGTGAACGTGGcaGAgGTGGG	chr1	119326861	+	3	0
No.3	AAGGTGAACGTGGATGAAGTNGG	tAtGTGtACGTGGATGAAGTGGG	chr2	60542664	+	3	0
No.4	AAGGTGAACGTGGATGAAGTNGG	AAGGTtAACtTGGATGAgGTTGG	chr2	196541035	-	3	0
No.5	AAGGTGAACGTGGATGAAGTNGG	gAGGTGAAaGTGGAaGAAGTGGG	chr2	240945918	-	3	0
No.6	AAGGTGAACGTGGATGAAGTNGG	AAGGTGcAgGTGGgTGAAGTGGG	chr17	63593297	-	3	0
No.7	AAGGTGAACGTGGATGAAGTNGG	AAGGTGAgCGTGGAgGcAGTGGG	chr16	85601541	-	3	0
No.8	AAGGTGAACGTGGATGAAGTNGG	AAGGaaAACGTGGAgGAAGTCGG	chr14	64338866	-	3	0
No.9	AAGGTGAACGTGGATGAAGTNGG	AAGGTGAACGaGGATGAgGaAGG	chr6	5849912	-	3	0
No.10	AAGGTGAACGTGGATGAAGTNGG	AAaGTGAACGTGGATGcAGTTGG	chr11	5234359	-	2	0
No.11	AAGGTGAACGTGGATGAAGTNGG	AAGGTGAACGTGGATGAAGTTGG	chr11	5226947	-	0	0
No.12	AAGGTGAACGTGGATGAAGTNGG	AAtGTGAAtGTGGATGAAaTGGG	chr11	18570832	-	3	0
No.13	AAGGTGAACGTGGATGAAGTNGG	AAtGTGAACGTGGATGAAaaTGG	chr10	60586199	+	3	0
No.14	AAGGTGAACGTGGATGAAGTNGG	AAGGTGAAtGTGGAgtAAGTGGG	chr18	63706608	+	3	0
No.15	AAGGTGAACGTGGATGAAGTNGG	AAaGTGAcCtTGGATGAAGTTGG	chr3	6827641	-	3	0
CD17 sgRNA	crRNA	DNA	Chromosome	Position	Direction	Mismatches	Bulge Size
No.1	CGTTACTGCCCTGTGGGGCANGG	CGTTtgTGCCCTGTGGGGCATGG	chr5	77084750	-	2	0
No.2	CGTTACTGCCCTGTGGGGCANGG	tGTTACTGCCCTGTGGttCAAGG	chr1	46263647	+	3	0

No.3	CGTTACTGCCCTGTGGGGCANGG	CGTcACTGCCgTGTGGcGCATGG	chr2	233314251	-	3	0
No.4	CGTTACTGCCCTGTGGGGCANGG	CagcACTGCCCTGTGGGGCAAGG	chr2	237360397	+	3	0
No.5	CGTTACTGCCCTGTGGGGCANGG	CGTgACTGCCCTGTGaGtCAGGG	chr6	156836330	+	3	0
No.6	CGTTACTGCCCTGTGGGGCANGG	CGTTACTGCCCTGTGGGGCAAGG	chr11	5226966	-	0	0
No.7	CGTTACTGCCCTGTGGGGCANGG	CGTcACTGaCCTGTGGGGCcAGG	chr19	40783764	-	3	0
No.8	CGTTACTGCCCTGTGGGGCANGG	gGTccCTGCCCTGTGGGGCAGGG	chr18	48170923	-	3	0
CD19 sgRNA	crRNA	DNA	Chromosome	Position	Direction	Mismatches	Bulge Size
No.1	CCTGTGGGGCAAGGTGAACGNGG	CCTGTGGGGCAAGGgGgACcAGG	chr1	3709556	+	3	0
No.2	CCTGTGGGGCAAGGTGAACGNGG	CCTGTGGGGgAAGGTaAAtGTGG	chr1	6965456	+	3	0
No.3	CCTGTGGGGCAAGGTGAACGNGG	CCTGTaGGGCAtGGgGAACGGGG	chr1	10719591	+	3	0
No.4	CCTGTGGGGCAAGGTGAACGNGG	CCTGTGGGGCAAGGTtcACGTGG	chr1	56356790	+	2	0
No.5	CCTGTGGGGCAAGGTGAACGNGG	CCTGTGGGGtAAGGTGAggGTGG	chr1	175868998	+	3	0
No.6	CCTGTGGGGCAAGGTGAACGNGG	CCTcTGGaGCAAGGTGAAgGTGG	chr1	233083163	+	3	0
No.7	CCTGTGGGGCAAGGTGAACGNGG	aagGTGGGGCAAGGTGAACGGGG	chr22	48118787	-	3	0
No.8	CCTGTGGGGCAAGGTGAACGNGG	CCTGTGGGGgAAGGTcAAtGGGG	chr2	51712473	-	3	0
No.9	CCTGTGGGGCAAGGTGAACGNGG	CCTGTGGGGCAgGGgGAAgGGGG	chr12	5589684	+	3	0
No.10	CCTGTGGGGCAAGGTGAACGNGG	CCTGTGGGGgAAGGTGAggGGGG	chr12	70127565	-	3	0
No.11	CCTGTGGGGCAAGGTGAACGNGG	CCTGTGGGaCAAGGctAACGTGG	chrX	16693369	+	3	0
No.12	CCTGTGGGGCAAGGTGAACGNGG	CCTGTGGaGCAAGagGAACGTGG	chrX	39991392	-	3	0
No.13	CCTGTGGGGCAAGGTGAACGNGG	CCTGTGGGGCAAGGTGAACGTGG	chr11	5226957	-	0	0
No.14	CCTGTGGGGCAAGGTGAACGNGG	CCTGTGGGGCAAaGTGAACGTGG	chr11	5234369	-	1	0
No.15	CCTGTGGGGCAAGGTGAACGNGG	CCTGTGGGGCAAGGTGAAtGTGG	chr11	5249740	-	1	0
No.16	CCTGTGGGGCAAGGTGAACGNGG	CCTGTGGGGCAAGGTGAAtGTGG	chr11	5254664	-	1	0
No.17	CCTGTGGGGCAAGGTGAACGNGG	CCTGTGGaGCAAGaTGAAtGTGG	chr11	5269826	-	3	0
No.18	CCTGTGGGGCAAGGTGAACGNGG	CCTGTtGGGgAAGGTGAACaGGG	chr19	13139598	-	3	0
CD26 sgRNA	crRNA	DNA	Chromosome	Position	Direction	Mismatches	Bulge Size
No.1	CGTGGATGAAGTTGGTGGTGNGG	CGTGGAaGAAGgTGGaGGTGGGG	chr8	138367823	-	3	0
No.2	CGTGGATGAAGTTGGTGGTGNGG	CGTGGAgGAAGagGGTGGTGTGG	chr5	3139007	-	3	0
No.3	CGTGGATGAAGTTGGTGGTGNGG	CGTGGATGAAGaTGGgGGTGTGG	chr7	158998085	-	2	0
No.4	CGTGGATGAAGTTGGTGGTGNGG	CGTGGATGAAGaTGGgGGTGTGG	chr7	158998187	-	2	0
No.5	CGTGGATGAAGTTGGTGGTGNGG	CGTGGcTaAAGTgGGTGGTGGGG	chr2	23572188	-	3	0
No.6	CGTGGATGAAGTTGGTGGTGNGG	gGTGtATGAAGTTaGTGGTGAGG	chr17	5703270	+	3	0
No.7	CGTGGATGAAGTTGGTGGTGNGG	CaTGGAgGAAGTTGGTGGgGGGG	chr17	73872265	-	3	0

No.8	CGTGGATGAAGTTGGTGGTGNGG	gGTGGgTGAgGTTGGTGGTGAGG	chrX	55833355	+	3	0
No.9	CGTGGATGAAGTTGGTGGTGNGG	CGTGGATGAAGTTGGTGGTGAGG	chr11	5226939	-	0	0
No.10	CGTGGATGAAGTTGGTGGTGNGG	CGTGGATGcAGTTGGTGGTGAGG	chr11	5234351	-	1	0
No.11	CGTGGATGAAGTTGGTGGTGNGG	tGTGcATGAAGcTGGTGGTGTGG	chr3	54294544	+	3	0
No.12	CGTGGATGAAGTTGGTGGTGNGG	CGTGGATGAAGaTGGTGaTtGGG	chr3	143298210	+	3	0
CD17/19/26 nick sgRNA	crRNA	DNA	Chromosome	Position	Direction	Mismatches	Bulge Size
No.1	CTCAGGAGTCAGATGCACCANGG	CTCAGGAGTCAGcTGCAggAAGG	chr8	95219189	+	3	0
No.2	CTCAGGAGTCAGATGCACCANGG	CTCAGGAGgCtGAgGCACCAGGG	chr8	115828378	-	3	0
No.3	CTCAGGAGTCAGATGCACCANGG	gTCAGGAGTCAGATGgtCCAGGG	chr8	138888718	+	3	0
No.4	CTCAGGAGTCAGATGCACCANGG	CTCtGGAGTCAGAaGCACCtGGG	chr15	60100014	+	3	0
No.5	CTCAGGAGTCAGATGCACCANGG	CTCAGGAGTCAGATtgcCCAAGG	chr15	83651008	+	3	0
No.6	CTCAGGAGTCAGATGCACCANGG	aTCAGGAGgCAGATGCAaCAGGG	chr15	91259465	+	3	0
No.7	CTCAGGAGTCAGATGCACCANGG	CTCAGGtGaCAGgTGCACCAAGG	chr5	159457784	+	3	0
No.8	CTCAGGAGTCAGATGCACCANGG	CTCAtGAGTCAGgTGCtCCAGGG	chr5	178388975	+	3	0
No.9	CTCAGGAGTCAGATGCACCANGG	CTCAGGAGTCtGggGCACCAGGG	chr20	61636724	-	3	0
No.10	CTCAGGAGTCAGATGCACCANGG	CTCtGGAGTCAGATGaACCAGGG	chr1	11453145	-	2	0
No.11	CTCAGGAGTCAGATGCACCANGG	CTCtGGAGTCAGATGCtCCtGGG	chr1	203447616	+	3	0
No.12	CTCAGGAGTCAGATGCACCANGG	CcCAGGAGTCAGATGaACCtGGG	chr22	23132220	-	3	0
No.13	CTCAGGAGTCAGATGCACCANGG	CTCAGGAGTCAGAaaaACCAGGG	chr22	35921102	+	3	0
No.14	CTCAGGAGTCAGATGCACCANGG	CaaAGGAGTCAGATGCtCCAAGG	chr7	43280153	+	3	0
No.15	CTCAGGAGTCAGATGCACCANGG	CaCAtGAGaCAGATGCACCAAGG	chr7	46712803	-	3	0
No.16	CTCAGGAGTCAGATGCACCANGG	CTCAGGAGTCAcATGCAgCtAGG	chr7	50341887	+	3	0
No.17	CTCAGGAGTCAGATGCACCANGG	CTCAGGAGTCtGATGCtgCAGGG	chr2	40322597	+	3	0
No.18	CTCAGGAGTCAGATGCACCANGG	CTCAGGAcTCAGAaGCAgCAGGG	chr2	111070024	+	3	0
No.19	CTCAGGAGTCAGATGCACCANGG	CTttGGgGTCAGATGCACCAGGG	chr4	38169177	+	3	0
No.20	CTCAGGAGTCAGATGCACCANGG	CTCtGGAGTCAGATcCACCtAGG	chr17	35779656	+	3	0
No.21	CTCAGGAGTCAGATGCACCANGG	CTCAGGAtgCAGAgGCACCAAGG	chr17	80695784	+	3	0
No.22	CTCAGGAGTCAGATGCACCANGG	CTCgGGAcTCgGATGCACCAGGG	chr17	80967791	+	3	0
No.23	CTCAGGAGTCAGATGCACCANGG	CTCAGGAGTCAGATGgACatGGG	chr16	8647937	-	3	0
No.24	CTCAGGAGTCAGATGCACCANGG	CTttGGAGTCAGATGCACCtGGG	chr9	78475740	-	3	0
No.25	CTCAGGAGTCAGATGCACCANGG	CTCAGGAGTCAGAcaCACtAAGG	chr9	85142374	+	3	0
No.26	CTCAGGAGTCAGATGCACCANGG	CTCtGGAGTCAGATGgACCtGGG	chr9	130438223	-	3	0
No.27	CTCAGGAGTCAGATGCACCANGG	CTCAGGAGTCtGATGaACCcAGG	chr14	38987580	+	3	0
No.28	CTCAGGAGTCAGATGCACCANGG	CTCtGGAGTCAGAaGCgCCAGGG	chr6	46243965	+	3	0

No.29	CTCAGGAGTCAGATGCACCANGG	CTCAGGAGTCAGATGCACCATGG	chr11	5234412	+	0	0
No.30	CTCAGGAGTCAGATGCACCANGG	CTCAGGAGTCAGATGCACCATGG	chr11	5227000	+	0	0
No.31	CTCAGGAGTCAGATGCACCANGG	CTCAGGAGTCAGgTcCACCtCGG	chr11	61741228	-	3	0
No.32	CTCAGGAGTCAGATGCACCANGG	CTCAGGgGTCtGATGCAtCAAGG	chr11	62603874	-	3	0
No.33	CTCAGGAGTCAGATGCACCANGG	CTCAGGAGTCAGATaCAggAGGG	chr11	64237838	+	3	0
No.34	CTCAGGAGTCAGATGCACCANGG	gTCAGaAGTCAGATGCcCCAGGG	chr10	131320294	-	3	0
No.35	CTCAGGAGTCAGATGCACCANGG	CcCAGGAGTCAGATGCACatAGG	chr3	194596760	-	3	0
IVS-I-1/5 sgRNA	crRNA	DNA	Chromosome	Position	Direction	Mismatches	Bulge Size
No.1	TGGTGAGGCCCTGGGCAGGTNGG	TGGaGAGGCCCTGGGgAGGgTGG	chr8	21265273	-	3	0
No.2	TGGTGAGGCCCTGGGCAGGTNGG	TtGTGAtGCCCTGGtCAGGTGGG	chr8	104322609	+	3	0
No.3	TGGTGAGGCCCTGGGCAGGTNGG	gGGcGAGGCCgTGGGCAGGTGGG	chr15	33311113	+	3	0
No.4	TGGTGAGGCCCTGGGCAGGTNGG	TcGTaAaGCCCTGGGCAGGTGGG	chr5	1341983	-	3	0
No.5	TGGTGAGGCCCTGGGCAGGTNGG	TGGTGAGGCatTGaGCAGGTAGG	chr5	54449162	-	3	0
No.6	TGGTGAGGCCCTGGGCAGGTNGG	gGGTGAGaCCaTGGGCAGGTGGG	chr5	134142881	+	3	0
No.7	TGGTGAGGCCCTGGGCAGGTNGG	TGGTGAGGCCCTGGGgtGGgTGG	chr5	140240613	+	3	0
No.8	TGGTGAGGCCCTGGGCAGGTNGG	TGGTGAGGggCTGGGCAGGgAGG	chr1	17554232	+	3	0
No.9	TGGTGAGGCCCTGGGCAGGTNGG	TGGTGAGGCCCaGGGagGGTGGG	chr1	27380510	-	3	0
No.10	TGGTGAGGCCCTGGGCAGGTNGG	gGccGAGGCCCTGGGCAGGTGGG	chr1	54281370	-	3	0
No.11	TGGTGAGGCCCTGGGCAGGTNGG	TacTGAGGCCCTGGGCAGGaTGG	chr1	116150564	-	3	0
No.12	TGGTGAGGCCCTGGGCAGGTNGG	gGGTGAGGCCCTGGGgAGGcAGG	chr1	160371650	+	3	0
No.13	TGGTGAGGCCCTGGGCAGGTNGG	gGGTGAGaCCCTGGGCAGGgAGG	chr22	22599219	-	3	0
No.14	TGGTGAGGCCCTGGGCAGGTNGG	TGGgGAGGtCaTGGGCAGGTGGG	chr22	23153370	-	3	0
No.15	TGGTGAGGCCCTGGGCAGGTNGG	TGaTGgGGCCCTGGGCAGGTAGG	chr7	44266688	-	2	0
No.16	TGGTGAGGCCCTGGGCAGGTNGG	aGaTGAGGCCCTGGGCAGGaAGG	chr7	127590052	-	3	0
No.17	TGGTGAGGCCCTGGGCAGGTNGG	TGGTGAtGCCCaGGGCtGGTGGG	chr2	7383148	+	3	0
No.18	TGGTGAGGCCCTGGGCAGGTNGG	TGcTGtGGCCCTGGGCtGGTCGG	chr2	218383021	-	3	0
No.19	TGGTGAGGCCCTGGGCAGGTNGG	TGGTaAGaCCCTGGGCAGGcGGG	chr2	237911485	-	3	0
No.20	TGGTGAGGCCCTGGGCAGGTNGG	TGGTGAGGCCgTGGGCAGGgAGG	chr2	239729702	+	2	0
No.21	TGGTGAGGCCCTGGGCAGGTNGG	TGGgGAGGCaCTGGGtAGGTGGG	chr12	112163302	-	3	0
No.22	TGGTGAGGCCCTGGGCAGGTNGG	TGGgGAGGCCgTGGGCAGGaCGG	chr12	130435569	-	3	0
No.23	TGGTGAGGCCCTGGGCAGGTNGG	TGGTGgtGCCCaGGGCAGGTGGG	chr12	132609259	+	3	0
No.24	TGGTGAGGCCCTGGGCAGGTNGG	TGGTGAGGggCTGGGCAtGTTGG	chr4	72373608	+	3	0
No.25	TGGTGAGGCCCTGGGCAGGTNGG	TGGTGAGGCCCTGGGggGcTTGG	chr17	39673492	-	3	0
No.26	TGGTGAGGCCCTGGGCAGGTNGG	aGGTGgGGCCCTGaGCAGGTTGG	chr17	79125192	+	3	0

No.27	TGGTGAGGCCCTGGGCAGGTNGG	TGGTGAaGCaCTGGGCgGGTGGG	chr16	14789621	+	3	0
No.28	TGGTGAGGCCCTGGGCAGGTNGG	TGGTGAaGCaCTGGGCgGGTGGG	chr16	15339276	-	3	0
No.29	TGGTGAGGCCCTGGGCAGGTNGG	TGGTGAaGCaCTGGGCgGGTGGG	chr16	16417211	+	3	0
No.30	TGGTGAGGCCCTGGGCAGGTNGG	TGGTGAaGCaCTGGGCgGGTGGG	chr16	18294645	-	3	0
No.31	TGGTGAGGCCCTGGGCAGGTNGG	gGGTGgGGCCCTGGGCAGGaTGG	chr16	50386362	+	3	0
No.32	TGGTGAGGCCCTGGGCAGGTNGG	gGGTGAGGCCCcGGcCAGGTGGG	chrX	7922227	-	3	0
No.33	TGGTGAGGCCCTGGGCAGGTNGG	TGGTGAGGCCCaGcGaAGGTAGG	chr14	20969464	-	3	0
No.34	TGGTGAGGCCCTGGGCAGGTNGG	TGGTGAGGgCCTctGCAGGTGGG	chr14	93040421	-	3	0
No.35	TGGTGAGGCCCTGGGCAGGTNGG	ccGTGAGGCCCTGGGaAGGTGGG	chr14	95504641	-	3	0
No.36	TGGTGAGGCCCTGGGCAGGTNGG	aGcTGAGGgCCTGGGCAGGTGGG	chr14	104786985	+	3	0
No.37	TGGTGAGGCCCTGGGCAGGTNGG	TGGTGAGGCCtTGGGCAtGgAGG	chr6	34021671	-	3	0
No.38	TGGTGAGGCCCTGGGCAGGTNGG	TGGTGAGGCCCTGGGCAGGTTGG	chr11	5226924	-	0	0
No.39	TGGTGAGGCCCTGGGCAGGTNGG	TGGTGAGGCCCTGGGCAGGTTGG	chr11	5234336	-	0	0
No.40	TGGTGAGGCCCTGGGCAGGTNGG	TtGTGgGGCCCTGGGCtGGTTGG	chr11	12206236	+	3	0
No.41	TGGTGAGGCCCTGGGCAGGTNGG	TGGTGAGGagCTGGGCtGGTGGG	chr11	64258418	+	3	0
No.42	TGGTGAGGCCCTGGGCAGGTNGG	TGGTGgGGCCCaGGGCAGaTGGG	chr11	71444248	-	3	0
No.43	TGGTGAGGCCCTGGGCAGGTNGG	gGGTGAGGCCCcGGGCAGcTGGG	chr11	72703304	-	3	0
No.44	TGGTGAGGCCCTGGGCAGGTNGG	TtGgGAGGCCCgGGGCAGGTGGG	chr10	15951550	-	3	0
No.45	TGGTGAGGCCCTGGGCAGGTNGG	TGGTGgGGCaCTtGGCAGGTGGG	chr10	128457509	+	3	0
No.46	TGGTGAGGCCCTGGGCAGGTNGG	TGGTtAGGCaaTGGGCAGGTGGG	chr13	18739234	-	3	0
No.47	TGGTGAGGCCCTGGGCAGGTNGG	aGGTGAGGgCCTGGcCAGGTGGG	chr13	112697058	+	3	0
No.48	TGGTGAGGCCCTGGGCAGGTNGG	TGGTtAGGCaaTGGGCAGGTGGG	chr18	14341366	+	3	0
No.49	TGGTGAGGCCCTGGGCAGGTNGG	TGGaGAGGCCCTGGGCAGGTGGG	chr18	78939382	-	1	0
No.50	TGGTGAGGCCCTGGGCAGGTNGG	TGGaGAGGCCCTGGGgAGGaAGG	chr3	44424917	-	3	0
No.51	TGGTGAGGCCCTGGGCAGGTNGG	TGGTcAGGCCCTGGGCAGGgAGG	chr3	140530825	-	2	0
IVS-I-1/5 nick sgRNA	crRNA	DNA	Chromosome	Position	Direction	Mismatches	Bulge Size
No.1	TCCACATGCCCAGTTTCTATNGG	TtCACATGCCCAGTTTCTtTTGG	chr5	78762393	-	2	0
No.2	TCCACATGCCCAGTTTCTATNGG	TCCACATGCaCAGTgTtTATTGG	chr14	27980926	+	3	0
No.3	TCCACATGCCCAGTTTCTATNGG	TCCACcaGCCCAGcTTCTATGGG	chr6	43444870	+	3	0
No.4	TCCACATGCCCAGTTTCTATNGG	TCCACATGtCCtGTTTtTATTGG	chr6	121990498	+	3	0
No.5	TCCACATGCCCAGTTTCTATNGG	TCCACATGCCCAGTTTCTATTGG	chr11	5226871	+	0	0
No.6	TCCACATGCCCAGTTTCTATNGG	TCCAtATGCCCAGTTTtTAaGGG	chr11	46437205	-	3	0
No.7	TCCACATGCCCAGTTTCTATNGG	TCCAaATGCCCAGTTTCctTAGG	chr11	72071981	-	3	0
No.8	TCCACATGCCCAGTTTCTATNGG	TCCcCATtCCCAGTTTCTcTTGG	chr11	92851552	-	3	0

CD39 sgRNA	crRNA	DNA	Chromosome	Position	Direction	Mismatches	Bulge Size
No.1	TGGTCTACCCTTGGACCCAGNGG	gGGTCTACtCTTtGACCCAGGGG	chr1	151599093	+	3	0
No.2	TGGTCTACCCTTGGACCCAGNGG	TaGcCTACCCTTtGACCCAGAGG	chr2	230951588	+	3	0
No.3	TGGTCTACCCTTGGACCCAGNGG	TGGTCTACCtTTGGACCaAGTGG	chr16	22327981	+	2	0
No.4	TGGTCTACCCTTGGACCCAGNGG	TtGTgaACCCTTGGACCCAGCGG	chr6	71620728	+	3	0
No.5	TGGTCTACCCTTGGACCCAGNGG	TGGTCTACCCTTGGACCCAGAGG	chr11	5226768	-	0	0
No.6	TGGTCTACCCTTGGACCCAGNGG	TGGTCTACCCTTGGACCCAGAGG	chr11	5234182	-	0	0
No.7	TGGTCTACCCTTGGACCCAGNGG	TtGTCTACCCaTGGACCCAGAGG	chr11	5254483	-	2	0
No.8	TGGTCTACCCTTGGACCCAGNGG	TtGTCTACCCaTGGACCtAGAGG	chr11	5243297	-	3	0
No.9	TGGTCTACCCTTGGACCCAGNGG	TtGTCTACCCaTGGACCCAGAGG	chr11	5249559	-	2	0
No.10	TGGTCTACCCTTGGACCCAGNGG	TccTCTcCCCTTGGACCCAGAGG	chr10	62668225	-	3	0
No.11	TGGTCTACCCTTGGACCCAGNGG	TGGTCTAgCCTTGGcCCCAcTGG	chr19	7626436	+	3	0
No.12	TGGTCTACCCTTGGACCCAGNGG	TGGgaTACCCTgGGACCCAGCGG	chr3	128934102	-	3	0
CD39 nick sgRNA	crRNA	DNA	Chromosome	Position	Direction	Mismatches	Bulge Size
No.1	GGGTGGGAAAATAGACCAATNGG	GGGTGGGAAAATAGACaAAaTGG	chr15	70203642	+	2	0
No.2	GGGTGGGAAAATAGACCAATNGG	GGtaGGGAAAAaAGACCAATAGG	chr5	103629305	-	3	0
No.3	GGGTGGGAAAATAGACCAATNGG	taGTGGaAAAATAGACCAATGGG	chr1	81273103	+	3	0
No.4	GGGTGGGAAAATAGACCAATNGG	GGcTGGGAAAATtGACCAgTGGG	chr1	111509291	-	3	0
No.5	GGGTGGGAAAATAGACCAATNGG	GaGTGGGAAAATAGctCAATTGG	chr1	236068972	+	3	0
No.6	GGGTGGGAAAATAGACCAATNGG	GGcTGGGgAAAgAGACCAATGGG	chr4	166435189	-	3	0
No.7	GGGTGGGAAAATAGACCAATNGG	GGGTGGGcggATAGACCAATGGG	chr16	5347776	-	3	0
No.8	GGGTGGGAAAATAGACCAATNGG	GGGTGGGAAAATgGAgaAATAGG	chr16	62752897	-	3	0
No.9	GGGTGGGAAAATAGACCAATNGG	GGGTGGGgAAATAGACaAAaTGG	chr6	83469734	-	3	0
No.10	GGGTGGGAAAATAGACCAATNGG	GGGTGGGAAAATAGACCAATAGG	chr11	5226803	+	0	0
No.11	GGGTGGGAAAATAGACCAATNGG	aGaTGGGAAAATAGACtAATGGG	chr13	78330130	-	3	0
No.12	GGGTGGGAAAATAGACCAATNGG	GGGTGaGAAAATAaAaCAATGGG	chr19	5066561	-	3	0
No.13	GGGTGGGAAAATAGACCAATNGG	GaGTGGGAAAAcAGACCAgTAGG	chr19	36012984	+	3	0
No.14	GGGTGGGAAAATAGACCAATNGG	GGGTGGGAAgAcAGACaAATAGG	chr3	49907322	-	3	0
CD71/72 sgRNA	crRNA	DNA	Chromosome	Position	Direction	Mismatches	Bulge Size
No.1	GTGAGCCAGGCCATCACTAANGG	aTGAGCCAGGCCAgCACTgAGGG	chr8	133274926	-	3	0
No.2	GTGAGCCAGGCCATCACTAANGG	GTGAGCCAGGCaAaCACTcAGGG	chr20	57134922	+	3	0
No.3	GTGAGCCAGGCCATCACTAANGG	GTGAGCCcGGCCAcCACTAgTGG	chr20	58391812	+	3	0

No.4	GTGAGCCAGGCCATCACTAANGG	GTGtGCCAGGCCcTCACTtAGGG	chr22	45990543	-	3	0
No.5	GTGAGCCAGGCCATCACTAANGG	GgGAGCCAGGCCAgCACTAtGGG	chr7	143063673	-	3	0
No.6	GTGAGCCAGGCCATCACTAANGG	GaGAGgCAGGCCATCAgTAAAGG	chr2	217882216	-	3	0
No.7	GTGAGCCAGGCCATCACTAANGG	aTGAGCCAGGCaAaCACTAATGG	chr17	51903484	+	3	0
No.8	GTGAGCCAGGCCATCACTAANGG	GTGAGCCAaGCCcTgACTAAAGG	chrX	10241084	-	3	0
No.9	GTGAGCCAGGCCATCACTAANGG	GTGAGCCAGGCCAgCACTttGGG	chr6	41959422	+	3	0
No.10	GTGAGCCAGGCCATCACTAANGG	tTaAGCCAGaCCATCACTAAGGG	chr6	123645765	-	3	0
No.11	GTGAGCCAGGCCATCACTAANGG	GTGAGCCAGGCCATCACTAAAGG	chr11	5226657	+	0	0
No.12	GTGAGCCAGGCCATCACTAANGG	GTGAGCCAGGCCATCACTAAAGG	chr11	5234071	+	0	0
No.13	GTGAGCCAGGCCATCACTAANGG	GTcAGCCAGGCCATgACTtACGG	chr11	72589153	+	3	0
No.14	GTGAGCCAGGCCATCACTAANGG	GTGAGCtgGGCCAgCACTAAAGG	chr3	49418947	-	3	0
CD71/72 nick sgRNA	crRNA	DNA	Chromosome	Position	Direction	Mismatches	Bulge Size
No.1	GGCTCACCTGGACAACCTCANGG	aGCTCACCTGGACAcCCTCcTGG	chr15	101307606	+	3	0
No.2	GGCTCACCTGGACAACCTCANGG	GGggCACCaGGACAACCTCAGGG	chr1	1495368	-	3	0
No.3	GGCTCACCTGGACAACCTCANGG	GGCTCACCTGGACAgaCTgAAGG	chr1	117588988	+	3	0
No.4	GGCTCACCTGGACAACCTCANGG	GGCTCACaTGGAtAACgTCAGGG	chr1	209064179	+	3	0
No.5	GGCTCACCTGGACAACCTCANGG	GGCTgAtCTGGACcACCTCAGGG	chr2	113309624	+	3	0
No.6	GGCTCACCTGGACAACCTCANGG	GGCTtcCCTGGgCAACCTCATGG	chr12	109267889	-	3	0
No.7	GGCTCACCTGGACAACCTCANGG	GtCTCACCTGGgCAcCCTCAGGG	chr12	114007671	-	3	0
No.8	GGCTCACCTGGACAACCTCANGG	GGCTCACCTtGACcACCcCAAGG	chr4	10613277	-	3	0
No.9	GGCTCACCTGGACAACCTCANGG	GGCTCACCTGGACAACCTCAAGG	chr11	5226641	-	0	0
No.10	GGCTCACCTGGACAACCTCANGG	GGCTCACCTGGACAACCTCAAGG	chr11	5234055	-	0	0
No.11	GGCTCACCTGGACAACCTCANGG	GGCTgACCcGGACAACCTCcAGG	chr11	77123740	+	3	0
No.12	GGCTCACCTGGACAACCTCANGG	aGCTCACCTGaACAACCaCAGGG	chr11	96554748	-	3	0
IVS-II-1 sgRNA	crRNA	DNA	Chromosome	Position	Direction	Mismatches	Bulge Size
No.1	CGTGGATCCTGAGAACTTCANGG	aGTGGgTCCTGAGAACTaCAAGG	chr1	160549792	+	3	0
No.2	CGTGGATCCTGAGAACTTCANGG	CGTGGATgCTGgGAgCTTCAGGG	chr2	55112633	-	3	0
No.3	CGTGGATCCTGAGAACTTCANGG	CcTGGATtCTGAGAAaTTCAAGG	chr16	60504053	-	3	0
No.4	CGTGGATCCTGAGAACTTCANGG	CGTGGATCCTGAGAACTTCAGGG	chr11	5233989	-	0	0
No.5	CGTGGATCCTGAGAACTTCANGG	CGTGGATCCTGAGAACTTCAGGG	chr11	5226575	-	0	0
No.6	CGTGGATCCTGAGAACTTCANGG	CGTGGAcCCTGAGAACTTCcTGG	chr11	5243104	-	2	0
No.7	CGTGGATCCTGAGAACTTCANGG	tGTGGATCCTGAGAACTTCAAGG	chr11	5249366	-	1	0
No.8	CGTGGATCCTGAGAACTTCANGG	tGTGGATCCTGAGAACTTCAAGG	chr11	5254290	-	1	0

No.9	CGTGGATCCTGAGAACTTCANGG	tGTGGATCCTGAGAACTTCAAGG	chr11	5269452	-	1	0
No.10	CGTGGATCCTGAGAACTTCANGG	CGTGGAcCCTGAGAACcTCcGGG	chr10	131260342	-	3	0
No.11	CGTGGATCCTGAGAACTTCANGG	CtTaGATCCTGtGAACTTCAGGG	chr13	89389953	-	3	0
No.12	CGTGGATCCTGAGAACTTCANGG	CcTGGATtCTGAGAgCTTCATGG	chr3	184461650	+	3	0
IVS-II-1 nick sgRNA	crRNA	DNA	Chromosome	Position	Direction	Mismatches	Bulge Size
No.1	GAACTTAACCATAGAAAAGANGG	tAACTTAACCAaAGAAgAGAAGG	chr8	43176754	-	3	0
No.2	GAACTTAACCATAGAAAAGANGG	aAACTTAACCAaAGAAAtGAAGG	chr8	57315512	+	3	0
No.3	GAACTTAACCATAGAAAAGANGG	GAACTTAAgCAaAGAAAAaATGG	chr5	15284601	+	3	0
No.4	GAACTTAACCATAGAAAAGANGG	GAACTTtACCATAGgAAAtATGG	chr5	29833980	-	3	0
No.5	GAACTTAACCATAGAAAAGANGG	GAACTTAtCCAgAGAgAAGATGG	chr5	78725417	-	3	0
No.6	GAACTTAACCATAGAAAAGANGG	GAACTgAAaCATAaAAAAGAAGG	chr5	112831752	-	3	0
No.7	GAACTTAACCATAGAAAAGANGG	GcACTTAACCATtGAAAAaAGGG	chr1	52102805	-	3	0
No.8	GAACTTAACCATAGAAAAGANGG	aAACTTAACCAcAGAAAAGtAGG	chr1	63976233	+	3	0
No.9	GAACTTAACCATAGAAAAGANGG	GAAaTTAAtCAgAGAAAAGAAGG	chr1	181725049	-	3	0
No.10	GAACTTAACCATAGAAAAGANGG	GAACTTtAaCAaAGAAAAGATGG	chr7	92435629	+	3	0
No.11	GAACTTAACCATAGAAAAGANGG	GAACTTAACCAaAGAAgtGAAGG	chr7	113023050	-	3	0
No.12	GAACTTAACCATAGAAAAGANGG	aAACTTAACCAaAGAAAtGAAGG	chr12	82208926	-	3	0
No.13	GAACTTAACCATAGAAAAGANGG	cAACTTAACCAaAGAAAtGAAGG	chr21	16875888	+	3	0
No.14	GAACTTAACCATAGAAAAGANGG	GAACTTAAaCATAGAcAAaATGG	chrX	51571348	+	3	0
No.15	GAACTTAACCATAGAAAAGANGG	GAtaTTAACCATAGAAAAGAAGG	chrX	119807445	-	2	0
No.16	GAACTTAACCATAGAAAAGANGG	GAACTgAACCAgAaAAAAGAAGG	chr6	152330468	-	3	0
No.17	GAACTTAACCATAGAAAAGANGG	GAACTTAACCATAGAAAAGAAGG	chr11	5226520	+	0	0
No.18	GAACTTAACCATAGAAAAGANGG	GAACTTAACCATAtAAAAcATGG	chr11	88694985	+	2	0
No.19	GAACTTAACCATAGAAAAGANGG	GAgaTTAAaCATAGAAAAGAAGG	chr13	27611955	+	3	0
No.20	GAACTTAACCATAGAAAAGANGG	tAtCTTAACCATAGAAAAtAAGG	chr13	72547367	-	3	0
No.21	GAACTTAACCATAGAAAAGANGG	GAACTTAtgCATgGAAAAGAAGG	chr18	30095766	-	3	0
No.22	GAACTTAACCATAGAAAAGANGG	GAAaTTAACCAaAGAAAAGAAGG	chr3	19205713	+	2	0
No.23	GAACTTAACCATAGAAAAGANGG	GAACTTAACCATcaAAAtGATGG	chr3	128714714	+	3	0
IVS-II-745 sgRNA	crRNA	DNA	Chromosome	Position	Direction	Mismatches	Bulge Size
No.1	AATAAAAGCAGAATGGTAGCNGG	AATAAAAGaAGAATGGTAtaAGG	chr8	77912456	+	3	0
No.2	AATAAAAGCAGAATGGTAGCNGG	AATAAAAaCAGAAaGGgAGCAGG	chr5	67092869	-	3	0
No.3	AATAAAAGCAGAATGGTAGCNGG	AATAAAAGaAtAAaGGTAGCAGG	chr5	86227158	+	3	0
No.4	AATAAAAGCAGAATGGTAGCNGG	AATAAAAGCAGAgTGtTgGCAGG	chr5	89710870	-	3	0

No.5	AATAAAAGCAGAATGGTAGCNGG	AAgAAgAGaAGAATGGTAGCTGG	chr5	131919788	+	3	0
No.6	AATAAAAGCAGAATGGTAGCNGG	AATAAAAGCAGcATGtTAGtAGG	chr20	9177194	+	3	0
No.7	AATAAAAGCAGAATGGTAGCNGG	AATAAAAGCAaAATGtaAGCTGG	chr20	33517937	-	3	0
No.8	AATAAAAGCAGAATGGTAGCNGG	AATAAAAGCccAATGGcAGCTGG	chr20	62003655	+	3	0
No.9	AATAAAAGCAGAATGGTAGCNGG	AATAAAAtCAttATGGTAGCAGG	chr1	7181517	+	3	0
No.10	AATAAAAGCAGAATGGTAGCNGG	AATAAAAGCAGggTGGaAGCAGG	chr1	19081596	+	3	0
No.11	AATAAAAGCAGAATGGTAGCNGG	AATAAAAGCAGctTGGaAGCAGG	chr1	23823211	-	3	0
No.12	AATAAAAGCAGAATGGTAGCNGG	cAaAAAAGCAGAAaGGTAGCTGG	chr1	75223230	-	3	0
No.13	AATAAAAGCAGAATGGTAGCNGG	AATAAAAGCAGAATGGgAaaAGG	chr2	53812385	+	3	0
No.14	AATAAAAGCAGAATGGTAGCNGG	AATAAAAaCAGAAaGGcAGCCGG	chr2	98742296	+	3	0
No.15	AATAAAAGCAGAATGGTAGCNGG	gAgAcAAGCAGAATGGTAGCTGG	chr2	206870719	-	3	0
No.16	AATAAAAGCAGAATGGTAGCNGG	AAgcAgAGCAGAATGGTAGCTGG	chr2	214478877	-	3	0
No.17	AATAAAAGCAGAATGGTAGCNGG	cATAAAAGCAGAgTGGTAaCAGG	chr2	231368509	+	3	0
No.18	AATAAAAGCAGAATGGTAGCNGG	AATAAAAGCAGAgTGGaAGCAGG	chr12	70791050	-	2	0
No.19	AATAAAAGCAGAATGGTAGCNGG	AAgAAAAGCAGAATGccAGCCGG	chr12	130367703	+	3	0
No.20	AATAAAAGCAGAATGGTAGCNGG	AAgAAAAGCAGAATaGTAaCTGG	chr21	33999273	-	3	0
No.21	AATAAAAGCAGAATGGTAGCNGG	AATAAAAGCAtAATGGagGCAGG	chr4	103812767	-	3	0
No.22	AATAAAAGCAGAATGGTAGCNGG	AATAAAAGCAGAgaGGaAGCGGG	chr17	82685180	+	3	0
No.23	AATAAAAGCAGAATGGTAGCNGG	AATAAAtGCAcAAaGGTAGCTGG	chr16	2881807	+	3	0
No.24	AATAAAAGCAGAATGGTAGCNGG	AATAAAAGCAcAATGcTAGCAGG	chr16	55387816	+	2	0
No.25	AATAAAAGCAGAATGGTAGCNGG	AATAAtAagAGAATGGTAGCAGG	chr9	25597846	+	3	0
No.26	AATAAAAGCAGAATGGTAGCNGG	AAcAAAAGCAagATGGTAGCAGG	chr9	73659555	+	3	0
No.27	AATAAAAGCAGAATGGTAGCNGG	AATAAAgGCAGAATGGcAtCGGG	chr9	87202244	+	3	0
No.28	AATAAAAGCAGAATGGTAGCNGG	AATAAAgGCAtAATGGgAGCTGG	chr9	88810679	-	3	0
No.29	AATAAAAGCAGAATGGTAGCNGG	AATtAAAGCAaAATGGTAGgTGG	chrX	44807078	-	3	0
No.30	AATAAAAGCAGAATGGTAGCNGG	AAgAAAAGCAGAAgGGTAGaAGG	chrX	98773192	+	3	0
No.31	AATAAAAGCAGAATGGTAGCNGG	AATAAAAGgAGAATGGgAGgAGG	chr14	40097881	-	3	0
No.32	AATAAAAGCAGAATGGTAGCNGG	AATcAcAGCAGAATGGaAGCTGG	chr14	63048893	+	3	0
No.33	AATAAAAGCAGAATGGTAGCNGG	AATAAAAtCAatATGGTAGCAGG	chr6	46564971	+	3	0
No.34	AATAAAAGCAGAATGGTAGCNGG	AATAAAAGCAGAATGGTAGCTGG	chr11	5225813	+	0	0
No.35	AATAAAAGCAGAATGGTAGCNGG	AAcAAAAGCAGAAgaGTAGCTGG	chr13	39402731	+	3	0
No.36	AATAAAAGCAGAATGGTAGCNGG	AATAAAAGCAGgtTGGTAGtGGG	chr13	42147206	+	3	0
No.37	AATAAAAGCAGAATGGTAGCNGG	AATAAAAGCAGAAgGGgAGtTGG	chr18	42175211	+	3	0
IVS-II-745 nick sgRNA	crRNA	DNA	Chromosome	Position	Direction	Mismatches	Bulge Size

No.1	TAAATTGTAACTGATGTAAGNGG	TAAATTGTAcCTcATtTAAGAGG	chr8	112833641	+	3	0
No.2	TAAATTGTAACTGATGTAAGNGG	TAAATTGctACTGATGTAtGGGG	chr8	139515013	+	3	0
No.3	TAAATTGTAACTGATGTAAGNGG	TAAATTaTAAaTcATGTAAGAGG	chr5	11348797	+	3	0
No.4	TAAATTGTAACTGATGTAAGNGG	TAAATgGTAtCTGgTGTAAGTGG	chr1	92065250	-	3	0
No.5	TAAATTGTAACTGATGTAAGNGG	TAAATTtTAAtTGAgGTAAGTGG	chr7	130561278	+	3	0
No.6	TAAATTGTAACTGATGTAAGNGG	TAAATTtTAAaTGATGaAAGTGG	chr2	41446312	+	3	0
No.7	TAAATTGTAACTGATGTAAGNGG	TAtATTGaAACTGgTGTAAGTGG	chr2	76075652	+	3	0
No.8	TAAATTGTAACTGATGTAAGNGG	TAttTaGTAACTGATGTAAGTGG	chr2	164693037	+	3	0
No.9	TAAATTGTAACTGATGTAAGNGG	TtAATTGTAAgTcATGTAAGTGG	chr2	187979210	+	3	0
No.10	TAAATTGTAACTGATGTAAGNGG	TAAAgTGTAACTcATGTAcGTGG	chr12	44707623	+	3	0
No.11	TAAATTGTAACTGATGTAAGNGG	TAAATTtTAACTGATGTctGTGG	chr4	116673927	-	3	0
No.12	TAAATTGTAACTGATGTAAGNGG	TAcATTGTAAgTGATaTAAGAGG	chr16	2417028	+	3	0
No.13	TAAATTGTAACTGATGTAAGNGG	gAAAaTGTAACTGAaGTAAGAGG	chrX	31243942	+	3	0
No.14	TAAATTGTAACTGATGTAAGNGG	TAAATTGTttCTGATtTAAGAGG	chrX	103239183	+	3	0
No.15	TAAATTGTAACTGATGTAAGNGG	TAAATTGTAACTGATGTAAGAGG	chr11	5225863	-	0	0
No.16	TAAATTGTAACTGATGTAAGNGG	TgAATTGTAtCTcATGTAAGTGG	chr13	104671581	+	3	0
No.17	TAAATTGTAACTGATGTAAGNGG	TAAATacTAACTGATaTAAGTGG	chr3	190711410	-	3	0

Supplemental Table 4: The Sanger sequencing primers for beta-thalassemia mutant off-target examination

Primer	5'-Sequence-3'	nt	note
88 spOT1-F	AGGTCAACAGCTTGCCCTTA	20	-88
88 spOT1-R	AAGAAATGGAATCCGGGAAC	20	
88 spOT2-F	GGAGGACTCAGCTTGGGTTA	20	
88 spOT2-R	CCTCACCTCAGCCTTCATGT	20	
88 spOT3-F	ATGACCACGCTGAGGCTAAT	20	
88 spOT3-R	CTAGGGGAGGATCTGGAGGA	20	
88 spOT4-F	GAAAAAGGGGTGAGGCAGAT	20	
88 spOT4-R	TGAATTCAGCACACAACACACT	22	
88 spOT5-F	AGGAAAGCGTGAATCCTTGA	20	
88 spOT5-R	GGTTACCTGCTGGCCAATAA	20	
88 niOT1-F	ATCTGCACAGGGAGTGCTTT	20	
88 niOT1-R	GCTTTGTGGAACAGGTGGAT	20	
88 niOT2-F	CCATTGCGGCTCATTTAGAG	20	
88 niOT2-R	CCTTTGGAAGGGATGGAGAC	20	
88 niOT3-F	GCCTCCAATAGCAACAGAGC	20	
88 niOT3-R	ATGCCCCTGAAATCCTCTCT	20	
88 niOT4-F	GTGGAAGGCATTGTGGTTAAG	21	
88 niOT4-R	TGCCTTTCACACTCAGGCTA	20	
88 niOT5-F	CCCTCATGCAGGAGAGAGAC	20	
88 niOT5-R	TGAAAAGTGGGGTGGGAAT	19	
CD6 spOT1-F	GGCTGCAAATCAGAATACCC	20	CD6
CD6 spOT1-R	CACACAGTGGAGCCTTCAAA	20	
CD6 spOT2-F	ACTCACCCAAGGACACCAAG	20	
CD6 spOT2-R	AGGGTGGCAGAGACAGAAGA	20	
CD6 spOT3-F	AGCCTGCTCAGAAGTGAAGC	20	
CD6 spOT3-R	AAGCTCTGCTGACGTTTTCC	20	
CD6 spOT4-F	CCAGTGCCAGACACATAGCA	20	
CD6 spOT4-R	GGAGAGTGCCATGTCAAGGT	20	
CD6 niOT1-F	GGTGTGAGCTGCGAATGATA	20	
CD6 niOT1-R	AAGGCTACACTCAGCCTCCA	20	
CD6 niOT2-F	TGGCCTTAGTCCCAAATCAA	20	
CD6 niOT2-R	GGGACTTAGGTGCCAGTCCT	20	

CD6 niOT3-F	ACTGCAAGTCAAAGGCTGCT	20	
CD6 niOT3-R	CTCCTGACCTCAGGTGATCC	20	
CD6 niOT4-F	GCCAACCCAGCTGATAGGTA	20	
CD6 niOT4-R	TGGCTGTTCCTCCCATTTAC	20	
CD6 niOT5-F	GCTCCTCAGACAGAGCATCC	20	
CD6 niOT5-R	CCTAGGAGTGCCCAGTTCAA	20	
CD17 spOT1-F	GAGCTTGGAAGCCTCGTTAT	20	
CD17 spOT1-R	TTGCCCAGTCTTGGGTATGT	20	
CD17 spOT2-F	AGAGGGGACAGAGGGAAAAA	20	
CD17 spOT2-R	CACGTCACTGCACTCCATCT	20	
CD17 spOT3-F	AGATGTTTGTCCCCACCTTG	20	
CD17 spOT3-R	AGGCTGAGGTGAGAGGATCA	20	
CD17 spOT4-F	AGCCCTTCATCACCCTTTCT	20	
CD17 spOT4-R	TCACGAGGAACAGTGTGGTC	20	
CD17 spOT5-F	AACAACGGAAATCCCACAAG	20	
CD17 spOT5-R	CCTTTGGGTCAGTGGTGAGT	20	
CD19 spOT1-F	CAGTGCTAACACCCCGTTTT	20	
CD19 spOT1-R	GCCTGAGTGTGGGAGAGAAG	20	
CD19 spOT2-F	GGGCATGCACATGTTTAAGT	20	
CD19 spOT2-R	GAGAAACCAGGGCACACAAT	20	
CD19 spOT3-F	CAAGACAAGGGACCCTGAAA	20	CD17/CD19/CD26
CD19 spOT3-R	CCATGCTCTTTGTGTGCAGT	20	
CD19 spOT4-F	AGCTTCCCAGAGGAAACTCC	20	
CD19 spOT4-R	TCAGGACCTTCTGAGGCTGT	20	
CD19 spOT5-F	CAAGGGGGCAGGTATTTGTA	20	
CD19 spOT5-R	AACTGGGGCTCAGTGTGATT	20	
CD26 spOT1-F	GCCAGGTTCTTTCTGAGTGG	20	
CD26 spOT1-R	GGGGGAGGAGATGAATTAGC	20	
CD26 spOT2-F	GGAGCTAACAGCCAAACAGG	20	
CD26 spOT2-R	TGATCCACACACTCGAAGGA	20	
CD26 spOT3-F	ATCACAGGGGGTGGTAACCT	20	
CD26 spOT3-R	TATTCATTCATCCCGGCACT	20	
CD26 spOT4-F	AAAGGGAGCAGAGCAAACAA	20	
CD26 spOT4-R	CACCCTGTTCTTCCCACACT	20	
CD26 spOT5-F	CCACACCCGGCTAATTTTTA	20	

CD26 spOT5-R	CCTCTGTGGGGACAAGAAAA	20	
CD26 niOT1-F	CATGACCCCTGGAAAGGATA	20	
CD26 niOT1-R	GAGTTGGGAGCTGTGGAGTC	20	
CD26 niOT2-F	TCCGAACTGCAGGAAAATAAA	21	
CD26 niOT2-R	CTCTGTCACCCAGGCTGTAGT	21	
CD26 niOT3-F	AAGGCCACTTGTGAGAGCAC	20	
CD26 niOT3-R	TCCTGGCTACCCCAATGTAA	20	
CD26 niOT4-F	GTCAGGAGACCGAGACCATC	20	
CD26 niOT4-R	AGCCAGGCTGAATATGATGC	20	
CD26 niOT5-F	TACCTTGGCCACTCACACAG	20	
CD26 niOT5-R	CGGTGTCTGCAACTCAAGAA	20	
IVS-1 spOT1-F	GAGACCAAAATGGCCAGATG	20	IVS-I-1/IVS-I-5
IVS-1 spOT1-R	AGAACAGGGCGAGAGATGTG	20	
IVS-1 spOT2-F	ACCGGATCTACACCATCAGC	20	
IVS-1 spOT2-R	CCACAGCCCTTGACAAGTTT	20	
IVS-1 spOT3-F	AGGACGAAAAGGAGGAGGAG	20	
IVS-1 spOT3-R	GTAGGGGAACAAGGGAGAGG	20	
IVS-1 spOT4-F	CCAGAAGCTGCTTTCCATCT	20	
IVS-1 spOT4-R	CGTCCCATTGTTTCTCGTTT	20	
IVS-1 spOT5-F	TCATGACCCCAAAATTGGTT	20	
IVS-1 spOT5-R	TCACTCCCTGACCAACTGC	19	
IVS-1 niOT1-F	GCCTGGAGAAGTCACCAAAG	20	
IVS-1 niOT1-R	CTGGTCCCAAGATTGGATTG	20	
IVS-1 niOT2-F	CGTGGACATAAGGCAGAGTG	20	
IVS-1 niOT2-R	CGCAAGCACATGCACTAAAT	20	
IVS-1 niOT3-F	GGGGCATTGGAATAGAATGA	20	
IVS-1 niOT3-R	CCCTCCAGAAGTCACAGCAT	20	
IVS-1 niOT4-F	TTTCAAAAACCCACCTTTGG	20	
IVS-1 niOT4-R	TCTGCAAATGTATGGAAACCA	21	
IVS-1 niOT5-F	ATGCTACCGGAGAAGCTGTT	20	
IVS-1 niOT5-R	CCCTCAAAACTCATCACCAA	20	
CD39 spOT1-F	AAGCATGTCCTGCAAAATCA	20	CD39
CD39 spOT1-R	GCCTTGCATCAGCTTTCTCT	20	
CD39 spOT2-F	GTTTGGGAGTGTTTCCCTGA	20	
CD39 spOT2-R	TCCCTGCTTACCCTCAGTTG	20	

CD39 spOT3-F	GACCATGGACAGTGAGCAAA	20	
CD39 spOT3-R	CTGAAGATGCCAACCCTGAT	20	
CD39 spOT4-F	ACTTTCCTCCAACTGCCTGA	20	
CD39 spOT4-R	AGACCTGGAGAAACCCCAGT	20	
CD39 spOT5-F	CATGGTGCATCTGACTCCTG	20	
CD39 spOT5-R	GAGCCAGGCCATCACTAAAG	20	
CD39 niOT1-F	TTGAATGGGAGGTGGGTTTA	20	
CD39 niOT1-R	GGGCTCAGAGAGGAACACAA	20	
CD39 niOT2-F	GTTCCACTGGCCAAAGATGT	20	
CD39 niOT2-R	TGGCTGGTTTCTCTCAATTTC	21	
CD39 niOT3-F	TGTGCACAAAGACAATGCAG	20	
CD39 niOT3-R	CCCCTTCATCCATGACTAGC	20	
CD39 niOT4-F	AGGGCATGAGAATCAACACC	20	
CD39 niOT4-R	GATGAGAATGGGGAAGACCA	20	
CD39 niOT5-F	ATTACAGGCATGCCACCAC	19	
CD39 niOT5-R	TTCCCTGGAATTGGAACACT	20	
7172 spOT1-F	AGTGTGACGACGGAAGCTCT	20	CD71/72
7172 spOT1-R	CAAGCATCACGGTTGAGAGA	20	
7172 spOT2-F	CCTTGCCCTCATGGATCTTA	20	
7172 spOT2-R	TCCCTATTTTGCCACCACTC	20	
7172 spOT3-F	AGTGATTCTCCTGCCTCAGC	20	
7172 spOT3-R	CAAGGAAGGACTGCTCAAGG	20	
7172 spOT4-F	CAGGTGGGATCACTGCAAAT	20	
7172 spOT4-R	TCATTCCTCTCCAGGACACC	20	
7172 spOT5-F	CAGACCCAGCCAAATGTCTT	20	
7172 spOT5-R	GAAAATCCCAGGAGCACAAA	20	
7172 niOT1-F	GACACAGGGGCTGAGTGTCT	20	
7172 niOT1-R	TCAGCTCGAGTCAGGTGTGT	20	
7172 niOT2-F	GCGCATCTTCTGTCGGTACT	20	
7172 niOT2-R	CTCCGCCTTGGGTTTTCTAT	20	
7172 niOT3-F	ATCTGCAAGCTCCACAGGAT	20	
7172 niOT3-R	CACTGCAAGGTACAGGGTCA	20	
7172 niOT4-F	GCAGGTGTTAGGGAAGCAAC	20	
7172 niOT4-R	GAGGAAGATTTCCCCTGGAG	20	
7172 niOT5-F	TACGCCAAGGTGAAGTTTCC	20	

7172 niOT5-R	GCTTCTGCAATGCTTGACTG	20	
II-1 spOT1-F	GGGAAGCACCTGTTTTCTCA	20	IVS-II-1
II-1 spOT1-R	ATTCCGAAGTGCAGTTTGCT	20	
II-1 spOT2-F	GGTTGCTCCAGAAACTCCAG	20	
II-1 spOT2-R	GGAAGCTCCACACTGACCTC	20	
II-1 spOT3-F	TTTGCCAAAACTTACTTGGAAGA	23	
II-1 spOT3-R	TGCAGATGCCAGAAACCATA	20	
II-1 spOT4-F	CCTAAGGTGAAGGCTCATGG	20	
II-1 spOT4-R	AGGGGGTTGGGAGAGAAAG	19	
II-1 spOT5-F	AGGGATTAGCCCGTTGTCTT	20	
II-1 spOT5-R	CAAGAGAAAGCCACCTCTGC	20	
II-1 niOT1-F	AAATGGGAAGAGAAGCCAAA	20	
II-1 niOT1-R	TGACTCTTCCAATCCCTGAA	20	
II-1 niOT2-F	AACCCACCCAGTTGTTTGTG	20	
II-1 niOT2-R	CTGGACCCGAAGCATAGAAA	20	
II-1 niOT3-F	TTCATGCCGTGAGTCTTCCT	20	
II-1 niOT3-R	CCTGCAAAGCATTGCTGAAT	20	
II-1 niOT4-F	CCAGTTACTTGGGAGGCTGA	20	
II-1 niOT4-R	TGCTCAGGTTTGTCCCTTTC	20	
II-1 niOT5-F	AACTGCCCCAGAAACACTCT	20	
II-1 niOT5-R	ACCAGCACTCTGTACTCCGT	20	
745 spOT1-F	CCTTCTTTCTGGGAAGGTGA	20	IVS-II-745
745 spOT1-R	GAGACGTCCATGGGCTAACT	20	
745 spOT2-F	AAAACATCTGATGGGAACACG	21	
745 spOT2-R	GTACTGGGCTGGAAGTCAGG	20	
745 spOT3-F	CAAAGACGTCAAGGAGAGCAG	21	
745 spOT3-R	TCAAACCCATGTTGCTCAAA	20	
745 spOT4-F	CCCCCGGCTAATTTTGTATT	20	
745 spOT4-R	CCCCCATCTGCTTAAGTGAC	20	
745 spOT5-F	CCAATGTGGAGTGATTTCCA	20	
745 spOT5-R	TTTTGCATTCACCTTTCCTG	20	
745 niOT1-F	GGCACTGGATAAAGCTTCCA	20	
745 niOT1-R	GGCAGGATGACAGATGAGGT	20	
745 niOT2-F	GATCTCTGCCCTGGAGAACA	20	
745 niOT2-R	GACAATGAGGACCAGCCAAT	20	

745 niOT3-F	CTGGCATTTTTCATAGATGTGC	22	
745 niOT3-R	TTGCCTTCCTGTTTCTGCTT	20	
745 niOT4-F	TCCCATTCTTTCCCTTGTTG	20	
745 niOT4-R	TGCCAATGCCCATAGTGTAA	20	
745 niOT5-F	GGTCCACACTGCTCTCACTTC	21	
745 niOT5-R	ACTCTGGCCGTTGATCATTT	20	

Supplemental Table 5: Primers for qPCR

Gene	forward (5'-3')	reverse (5'-3')
HBA	AACTTCAAGCTCCTAAGCCA	CAGGAACTTGTCCAGGGAG
HBG	GAACTTCAAGCTCCTGGGA	AGTCACCATCTTCTGCCAG
HBB	GAGAACTTCAGGCTCCTGG	ACTTTCTGATAGGCAGCCTG
GAPDH	TCATTTCCTGGTATGACAACGA	GTCTTACTCCTTGGAGGCC
BCL11A	GGGTATTTGTAAAGATGAGCCC	TGCAAGAGAAACCATGCAC
GATA1	ACTACCTATGCAACGCCTG	TTACTGACAATCAGGCGCT
GATA2	CAAGCTGCACAATGTTAACAG	GACTTGTTGGACATCTTCCG
GYPA	TCTTACGGTATTCGCCGAC	TCATTGATCACTTGTCTCTGGA
KLF1	TGACTTCCTCAAGTGGTGG	CTCAGACTTCACGTGGAGG
EPOR	GAGACACCCATGACGTCTC	GATCTCCACCCTCTGTACG
GFI1B	GTGAAGTGCAACAAGGAGC	GTCTGAGTGGATGAGCAGG
MYB	AATGTCTCCAGTCATGTTCC	ATAGTGTCTCTGAATGGCTG
Band3	ATGAAGACCCAGACATCCC	CCACATAGACCTTGTGGGT
Band4.1	TCAGACTCGGAATCCAAGC	CTTGTCAGTGACCTCCCTC
Ank1	TGAGTTTCAGAATATTCCAGGG	TGCGAATGATCTTCTTGGT
SPTA1	AAATGACTTTGCTGTCCATGAG	TCCTGCAACACCTTATTTAGGA
AHSP	TCAAGAGTGTGGGAGTTCAG	AGTCACCATGTCTTCTTCAGAG
E2F2	CAACTTTAAGGAGCAGACAGTG	CAGGTTGTCCTCAGTCCTG
FOG1	CAGCGCAGATGTTAACTCAC	CTGGTTCCTGTCCTTCCAG
BCL-xL	GGCGGATTTGAATCTCTTTCTC	AGAAAGTCAACCACCAGCT
HSP70	CTGGAACTATGAATAGAGGCA	AAACACTACGAAGTGGTGG
Caspase-3	AATGGATTATCCTGAGATGGG	GACCGAGATGTCATTCCAG
JAK2	GACAGATGATCGTGTTCCA	TCATATAGATCTCATCTGGGCA
FAS-L	ATTTAACAGGCAAGTCCAACTC	TCACTCCAGAAAGCAGGAC
PRDX2	CTACAAAGGGAAGTACGTGGT	GAACTGAGAGTCCACCGAG
TWSG1	AATGCCTCATTCAGGAGCT	CAAGACACAGCATGCACTC
LYZ	TTGAAAGGTGTGAGTTGGC	AACACATCCAGTTTGCTAGG
TUBB6	AAGTGATCAGCGATGAGCA	CACATATTTCTGAGACGATGACTC
UCHL1	ATTGGGAATTCCTGTGGCA	TGATCCATCCTCAAATCCCAG
SMAD1	TATGAGCTTTGTGAAGGGCT	AGATGTATCTCAATCCAGCAGG
TGFB3	GAAATCAAATTCAAAGGCGTGG	GTGATCCTTCTGCTTCTTGAG
STAT5	TTCAACAGGGAGAACTTGC	GTGCTTCTTCAACACCTCC

BCL-xL	GGCGGATTTGAATCTCTTTCTC	AGAAAGTCAACCACCAGCT
HSP70	CTGGAACTATGAATAGAGGCA	AAACACTACGAAGTGGTGG
Caspase-3	AATGGATTATCCTGAGATGGG	GACCGAGATGTCATTCCAG
JAK2	GACAGATGATCGTGTTCCA	TCATATAGATCTCATCTGGGCA
FAS-L	ATTTAACAGGCAAGTCCAACTC	TCACTCCAGAAAGCAGGAC
PRDX2	CTACAAAGGGAAGTACGTGGT	GAACTGAGAGTCCACCGAG
TWSG1	AATGCCTCATTCAGGAGCT	CAAGACACAGCATGCACTC
LYZ	TTGAAAGGTGTGAGTTGGC	AACACATCCAGTTTGCTAGG
TUBB6	AAGTGATCAGCGATGAGCA	CACATATTTCTGAGACGATGACTC
UCHL1	ATTGGGAATTCCTGTGGCA	TGATCCATCCTCAAATCCCAG
SMAD1	TATGAGCTTTGTGAAGGGCT	AGATGTATCTCAATCCAGCAGG
TGFB3	GAAATCAAATTCAAAGGCGTGG	GTGATCCTTCTGCTTCTTGAG
STAT5	TTCAACAGGGAGAACTTGC	GTGCTTCTTCAACACCTCC
AKT1	CTTCTTTGCCGGTATCGTG	TGCTGTCATCTTGGTCAGG
CASP8	CTGGATGATGACATGAACCTG	GATGTCCAACTTTCCTTCTCC
CASP9	AAACCAGAGGTTCTCAGACC	AACTCTCAAGAGCACCGAC
TNF	CTCTAATCAGCCCTCTGGC	GAGGGTTTGCTACAACATGG
TNFSF10	GATCTTCACAGTGCTCCTG	TACTTGTCCTGCATCTGCT
GDF15	TAGACAAGGAGAGCCAGGA	ATGAGGACACCATTCCAGC
GDF11	ATCAACGCCTTTGATCCCA	CTCCATGAATGGATGCAGC
CCNA2	GCCATCAGTTATTGCTGGA	CGTATTAATGATTCAGGCCAG
CDK2	CTCATCAAGAGCTATCTGTTCC	TTTAAGGTCTCGGTGGAGG
TFRC	CAGCAAAGTTGAGAAACTCAC	CAGAAAGAAACTGCTGGGA
SMAD2	AGGACGATTAGATGAGCTTGAG	GAGCAAGTGCTTGGTATGG
SMAD3	ACTACAGCCATTCCATCCC	TAGGTTTGGAGAACCTGCG
CD79A	GAACCGAATCATCACAGCC	CCATCGTTTCCTGAACAGC
CRHBP	TTCTTCCGAGTCCATGAACC	AATGACATTGCAAGGAAAGAGG

Supplemental Table 6: Raw data of qPCR

**Ct values for qPCR**													
raw data		Before erythroid differentiation											
		WT	-88	CD6	CD17	CD19	CD26	IVS-I-1	IVS-I-5	CD39	CD7172	IVS-II-1	IVS-II-745
well	Gene	Ct value	Ct value	Ct value	Ct value	Ct value	Ct value	Ct value	Ct value	Ct value	Ct value	Ct value	Ct value
A1	*HBA*	17.53	15.97	14.27	15.26	16.6	18.39	14.94	16.11	16.27	14.86	14.96	14.75
A2		16.66	15.62	13.82	15.27	16.46	18.1	15.94	15.79	15.61	14.68	15.16	14.16
A3		17.09	15.54	13.97	15.48	16.44	18.48	16.16	15.77	15.96	14.84	15.43	14.74
A4	*KLF1*	23.56	24.12	22.84	23.09	23.58	23.7	23.43	23.63	23.25	23.23	23.88	22.99
A5		23.6	24.05	22.92	22.7	23.33	23.38	23.09	23.33	23.06	23.21	23.5	22.96
A6		23.55	23.89	22.28	22.58	23.33	23.23	22.97	23.26	22.92	22.95	22.97	22.85
A7	*AHSP*	27	26.36	25.21	26.39	27.69	28.79	26.35	26.34	26.44	26.26	24.6	26.05
A8		27.03	26.85	25.76	26.33	27.79	27.85	25.99	26.3	26.21	26.07	24.45	26.02
A9		26.88	26.76	24.94	26.19	27.05	27.69	25.9	26.35	26.29	26.02	24.56	26.53
A10	*PRDX2*	17.49	16.94	16.56	16.3	17.08	18.22	16.6	16.67	16.39	16.22	16.57	17.24
A11		17.39	16.94	15.96	16.44	17.04	18.18	16.56	16.68	16.53	16.41	16.41	16.81
A12		17.31	16.92	15.86	16.43	17.09	18.21	16.64	16.55	16.6	16.44	16.47	16.81
B1	*HBB*	12.41	12.12	12.68	11.91	13.57	13.46	16.22	15.46	17.38	15.79	16.2	16.61
B2		12.61	12.13	12.85	12.11	13.51	13.2	16.35	15.16	17.38	15.8	16.53	16.78
B3		12.66	12.13	12.82	12.03	13.53	13.2	16.36	15.17	17.47	15.81	16.51	16.8
B4	*EPOR*	25.17	25.02	24.41	25.01	25.68	25.8	25.13	25.11	25.53	25.13	25.91	25.51
B5		25.11	24.85	23.93	24.87	25.55	25.79	25.01	24.91	25.44	24.99	25.94	25.23
B6		24.76	24.54	23.59	24.65	24.91	25.39	24.62	24.39	25.02	24.63	25.07	24.88
B7	*E2F2*	24.99	23.4	23.14	23.91	24.87	25.36	24.69	24.76	24.97	23.78	24.7	24.21
B8		25.14	23.43	23.17	24.04	25.1	25.52	24.82	24.95	24.82	23.87	24.61	24.42
B9		25.15	23.53	23.51	24.09	25.14	25.35	24.84	25.03	24.95	23.87	24.68	24.42
B10	*TWSG1*	26.1	26.75	26.42	26.67	25.9	26.05	26.16	25.91	25.87	26.14	26.42	26.68
B11		26.11	26.74	26.62	26.42	25.94	25.97	25.95	25.98	25.39	26.12	26.4	26.69
B12		26.8	26.78	26.44	27	25.88	26.1	26.76	26.44	25.55	26.68	26.65	27.23
C1	*HBG*	21.18	20.37	18.5	15.77	21.85	22.82	18.81	19.37	11.82	17.18	15.66	14.98
C2		20.92	20.46	18.6	15.87	21.84	22.85	18.92	18.96	11.82	17.18	15.65	14.85
C3		20.88	20.37	18.67	15.89	21.83	22.83	18.91	19.01	11.88	17.21	15.71	14.85
C4	*GF1B*	24.54	24.65	24.15	24.54	23.91	25.01	24.62	24.66	24.33	24.84	24.74	25.64
C5		24.45	24.63	24.01	24	23.7	24.99	24.72	24.51	24.57	24.77	24.72	25.54

C6		24.57	24.68	23.99	24.02	23.87	24.81	24.55	24.54	24.05	24.7	24.46	25.19
C7	*TAL1*	30.99	30.57	29.77	30.12	31.07	30.31	30.11	30.64	31.05	29.87	31.12	30.63
C8		31.14	30.79	30.62	30.6	31.34	30.96	30.56	30.29	31.47	29.81	32.53	31.1
C9		31.03	30.74	29.78	29.95	30.63	30.19	30.02	30.95	31.39	29.79	31.47	30.62
C10	*LYZ*	32.45	35.32	33.37	34.7	35.34	32.27	32.71	34.57	32.58	33.54	35.32	33.48
C11		32.96	35.44	34.31	34.32	35.85	30.96	33.38	34.54	32.39	33.96	35.46	32.85
C12		34.36	34.86	34.18	33.66	35.02	32.11	33.8	34.43	32.7	34.87	35.4	33.89
D1	*GAPDH*	17.81	18.56	18.41	17.65	17.33	17.47	17.39	17.05	16.61	17.5	17.36	17.9
D2		17.68	18.55	18.37	17.66	17.18	17.41	17.57	17.01	16.7	17.5	17.15	17.65
D3		17.6	18.48	18.36	17.76	17.17	17.41	17.49	17.04	16.73	17.47	17.13	17.63
D4	*C-MYB*	20.98	22.51	22.37	21.67	20.85	20.69	21.09	20.89	20.54	21.94	21.69	22.16
D5		20.99	22.59	22.15	21.65	20.83	20.62	21.06	20.91	20.54	21.9	21.73	22.09
D6		20.95	22.5	22.15	21.61	20.83	20.66	21.03	20.85	20.53	21.9	21.67	22.09
D7	*BCL-xL*	23.76	23.2	22.91	23.15	23.53	23.88	23.5	23.76	23.64	23.38	23.74	23.88
D8		23.78	23.36	22.92	23.18	23.54	23.66	23.6	23.76	23.62	23.38	24.09	23.77
D9		23.8	23.4	22.98	23.34	23.51	23.54	23.5	23.82	23.43	23.39	23.8	23.82
D10	*TUBB6*	24.7	24.93	24.58	24.78	24.67	26.81	24.44	24.49	24.95	24.76	24.72	24.71
D11		24.68	24.85	24.54	24.82	24.63	25.01	24.6	24.65	24.53	24.68	24.84	24.83
D12		25.33	25.76	25.18	24.84	25.34	24.68	24.93	25.02	25.18	25.15	25.2	25.19
E1	*BCL11A*	24.62	23.79	24.42	23.98	23.92	24.14	24.3	23.91	25.59	24.8	24.53	26.63
E2		24.43	23.86	24.06	24.4	23.89	24.31	24.36	23.75	25.77	24.81	24.75	25.92
E3		24.18	23.8	24.01	24.28	23.86	24.2	24.36	23.75	25.63	24.85	24.53	25.98
E4	*Band3*	23.73	21.91	20.8	21.45	23.82	25.74	22.16	23.1	22.99	21.55	22.56	22.05
E5		23.72	21.91	20.8	21.45	23.84	25.68	22.19	23.1	23.02	21.54	22.57	22.09
E6		23.77	21.87	20.71	21.33	23.78	25.69	22.19	23.09	23	21.55	22.48	22.05
E7	*HSP70*	21.82	22.62	22.17	21.88	21.59	23.72	21.86	21.48	21.15	21.68	21.74	22.15
E8		21.85	22.73	22.22	21.83	21.5	21.93	21.91	21.43	21.21	21.8	21.8	22.18
E9		21.85	22.76	22.35	21.89	21.61	21.75	21.85	21.57	21.16	21.67	21.77	22.13
E10	*UCHL1*	28.96	37.43	33.67	28.71	26.79	37.73	31.98	29.96	27.81	31.45	28.99	32.25
E11		28.56	34.51	37.76	28.91	26.75	34.26	33.29	30.86	27.54	31.94	29.53	32.68
E12		29.77	35.93	34.25	29.06	27.94	35.22	32.99	31.19	27.79	33.44	29.92	32.93
F1	*GATA1*	23.5	23.56	22.94	22.96	23.19	22.89	23.04	23.67	23.46	23.46	23.65	24.18
F2		23.49	23.27	22.98	23.19	23.1	22.81	23.04	23.66	23.45	23.2	23.68	23.87
F3		23.46	23.44	22.94	23.05	23.08	22.85	23.03	23.63	23.48	23.14	23.67	23.89
F4	*Band4.1*	22.9	22.42	22.08	22.2	22.68	22.68	22.6	22.88	22.67	22.38	23.08	22.9

F5		22.89	22.41	22.11	22.2	22.64	22.68	22.62	22.91	22.67	22.39	23.02	22.89
F6		22.91	22.41	22.04	22.21	22.65	22.69	22.58	22.86	22.64	22.21	22.98	22.92
F7	*Caspase-3*	22.4	23.04	22.81	22.72	22.66	22.09	22.39	21.98	22.26	22.57	22.73	22.84
F8		22.2	23.01	22.79	22.44	22.58	22.08	22.41	21.97	21.91	22.6	22.71	22.81
F9		22.16	23.02	22.83	22.58	22.59	22.1	22.45	21.99	22.13	22.6	22.66	22.84
F10	*SMAD1*	29.58	33.25	34.14	27.04	33.7	35.58	33.64	33.29	34.53	33.67	32.75	33.65
F11		29.41	33.66	34.74	27.14	32.97	34.43	33.93	33.59	37.18	32.91	32.99	33.59
F12		30.02	35.71	34.83	27.19	32.85	36.91	33.77	33.39	32.89	32.88	33.3	34.24
G1	*GATA2*	23.68	25.13	24.1	24.55	22.89	23.56	23.23	22.94	23.57	24.52	23.93	24.25
G2		23.7	25.19	24.17	25	22.87	23.65	23.08	22.95	23.69	24.61	23.98	24.4
G3		23.69	25.41	24.22	24.85	22.84	23.67	23.02	22.91	23.61	24.62	23.96	24.36
G4	*Ank1*	20.36	19.93	19.46	19.65	19.94	20.05	19.91	20	19.77	19.57	20.24	20.12
G5		20.22	19.8	19.42	19.58	19.91	20.03	19.86	19.95	19.77	19.47	20.42	20.14
G6		20.34	19.9	19.39	19.51	19.93	20	19.9	19.97	19.8	19.48	20.21	20.03
G7	*JAK2*	23.97	23.43	23.12	23.3	23.61	23.98	23.63	23.4	23.5	23.27	23.55	23.75
G8		23.75	23.39	23.14	23.31	23.56	23.95	23.82	23.34	23.43	23.21	23.46	23.62
G9		23.9	23.35	23.38	23.27	23.43	23.96	23.61	23.44	23.47	23.22	23.47	23.75
G10	*TGFB3*	28.56	29.68	29.6	29.17	28.63	28.1	28.05	28.62	29.04	28.25	29.25	29.81
G11		28.71	29.5	29.47	29.02	28.47	28.05	27.97	28.52	28.87	28.72	29.4	29.73
G12		28.91	29.88	29.97	29.72	28.48	28.65	28.18	28.96	29.92	29.13	29.95	30.33
H1	*GYPA*	19.79	19	17.94	18.58	19.17	20.18	19.38	19.12	19.03	18.18	17.79	18.89
H2		19.87	18.98	17.97	18.64	19.09	20.34	19.21	19.06	19.39	18.5	17.78	18.92
H3		19.86	18.97	17.99	19.09	19.13	20.16	19.38	19.05	19.3	18.3	18.41	18.89
H4	*SPTA1*	21.11	20.76	20.4	21.45	21.12	21.06	21.17	20.71	21.27	20.85	21.48	20.96
H5		21.13	20.73	20.4	21.39	21.09	21.05	21.12	20.73	21.29	20.92	21.28	20.93
H6		21.13	20.71	20.44	21.14	21.03	21.08	21.14	20.71	21.01	21.12	20.95	20.96
H7	*FAS-L*	35.5	33.4	31.88	27.58	32.86	30.13	29.66	32.36	34.42	35.22	34.23	35.28
H8		35.41	35.33	30	28.58	32.59	31.14	32.78	34.86	34.35	34.58	33.61	34.44
H9		35.41	35.24	29.71	27.58	33.7	32.61	31.36	34.98	34.13	33.97	34.87	34.73
H10	*STAT5*	22.95	24.68	24.22	23.66	23.29	22.48	22.86	23.27	23.23	24.13	24.45	23.55
H11		22.98	24.68	24.18	23.28	23.29	22.44	22.71	23.01	23.08	23.98	24.56	23.53
H12		22.97	24.72	24.19	23.63	23.32	22.45	22.85	23.01	23.08	23.8	24.35	23.59
A1	*Caspase-9*	28.7	28.68	28.54	28.68	28.72	28	28.4	29.18	29.1	28.81	29.59	28.76
A2		28.8	28.51	28.76	28.86	28.71	28.15	28.83	28.89	28.86	29.31	28.95	29.06

A3		28.43	28.74	28.99	29.4	28.58	28.3	29.32	29.76	29.26	29.2	29.26	29.12
A4	*Cyclin-A*	20.72	21.12	20.58	20.89	20.79	21	20.98	20.48	20.33	20.6	20.42	20.49
A5		20.97	21.46	20.58	20.9	20.82	21.12	21.02	20.27	20.47	20.54	20.5	20.83
A6		20.96	21.39	20.61	21.03	20.74	21.12	20.95	20.3	20.32	20.67	20.38	20.76
A7	*SOX6*	26.51	24.43	23.48	25.57	25.79	26.92	25.24	25.34	27.11	26.21	25.1	27.24
A8		26.16	24.02	23.42	25.4	25.67	27.36	24.91	25.41	27.16	25.84	25.18	26.99
A9		26.45	24.07	23.15	25.39	25.56	27.15	25.06	25.39	27.1	25.93	25.2	27.07
A10	*TNF*	35.17	37.41	35.37	37.29	37.5	35.77	37.49	35.34	30.66	37.48	37.65	35.51
A11		33.76	35.77	35.34	37.64	37.7	34.12	37.01	36.78	30.08	37.66	37.71	34.76
A12		33.62	34.95	36.69	35.57	37.72	34.51	35.29	34.73	30.04	35.88	35.89	34.97
B1	*NFE4*	35.29	35.08	35.94	36.82	35.66	36.77	34.44	34.4	34.89	37.22	36.02	36.77
B2		35.11	35.22	35.87	36.93	36.09	36	34.15	32.52	35.99	37.39	37.12	35.29
B3		35.02	35.01	35.99	36.77	36.71	36.65	35.77	34.41	36.14	37.19	36.75	37.52
B4	*CDHBP*	27.92	25.59	29.81	28.27	26.75	27.86	30.88	26.79	26.86	27.61	26.65	27.7
B5		28.13	25.5	29.89	28.8	26.92	27.74	31.31	26.97	26.8	27.56	26.77	27.38
B6		27.87	25.21	30.02	28.46	26.82	27.81	31.22	26.91	26.67	27.4	26.8	27.55
B7	*AKT*	24.28	24.75	24.93	24.93	23.75	23.81	23.81	24.31	24.42	24.64	24.93	24.78
B8		24.84	25.05	25.54	25.28	24.01	24.2	23.95	24.76	25.02	25.41	25.29	25.41
B9		25.02	25.65	25.43	25.53	24.31	24.41	24.36	24.69	25.06	25.3	25.54	25.52
B10	*TFR1*	17.46	17.46	16.98	17.52	17.2	17.49	18.49	17.74	17.47	18.04	17.91	18.2
B11		17.41	17.48	17.01	17.07	17.34	17.48	18.5	17.73	17.47	18.05	17.94	18.22
B12		17.37	17.42	17.1	17.32	17.27	17.51	18.81	17.76	17.52	18.49	18.22	18.37
C1	*NFE2*	21.47	21.59	20.82	21.38	21.44	21.18	21.65	21.78	21.67	21.59	21.6	21.92
C2		21.41	21.48	20.74	21.11	21.36	21.09	21.43	21.73	21.56	21.59	21.49	21.86
C3		21.54	21.46	20.7	21.12	21.44	21.12	21.45	21.75	21.57	21.59	21.51	21.89
C4	*SMAD2*	22.91	23.16	23.06	22.95	22.72	22.72	23.38	22.72	23.11	22.93	22.95	23.09
C5		22.9	23.2	23.06	22.89	22.76	22.71	23.38	22.75	23.09	22.94	23	23.1
C6		22.9	23.17	23.01	22.85	22.72	22.71	23.39	22.72	23.07	22.95	22.96	23.06
C7	*CDK2*	23.42	23.67	23.41	23.39	23.33	23.28	23.63	23.37	23.66	23.47	23.92	23.63
C8		23.51	23.86	23.48	23.48	23.3	23.29	23.67	23.43	23.62	23.46	23.9	23.6
C9		23.57	23.67	23.52	23.53	23.45	23.43	23.67	23.44	23.58	23.62	23.89	23.82
C10	*GDF11*	27.55	31.21	29.48	28.87	27.7	26.58	28.23	27.58	27.57	29.16	29.44	29.19
C11		27.99	30.24	29.53	28.98	27.59	26.74	27.86	27.56	27.54	29.51	29.06	29.17
C12		27.92	31.4	30.08	29.47	27.93	27.03	28.87	27.84	27.83	29.59	30.15	29.62
D1	*LYAR*	22.45	23.3	23.38	22.56	21.79	22.31	22.77	22.14	21.71	22.73	21.83	23.25

D2		22.07	23.06	23.08	22.22	21.9	21.99	22.06	21.87	21.37	22.58	21.58	22.7
D3		22.05	22.98	23.02	22.23	21.86	22.02	22.05	21.91	21.4	22.49	21.62	22.77
D4	*Caspase-8*	25.84	26.78	26.4	26.43	25.89	25.52	25.67	26.16	25.81	26.09	26.59	26.95
D5		25.76	26.67	26.54	26.52	25.85	25.51	25.68	26.16	25.86	26.14	26.43	27
D6		25.74	26.5	26.52	26.44	25.82	25.55	25.82	26.07	25.93	26.05	26.46	26.83
D7	*GDF15*	34.92	32.83	32.75	32.73	33.4	34.19	36.11	34.53	35.41	34.92	37.35	35.27
D8		37.67	33.33	32.57	35.67	34.38	33.47	35.28	36.02	33.45	35.2	34.11	34.95
D9		36.55	33.48	32.69	36.15	34.93	34.4	33.31	34.93	35.15	33.86	35.64	35.53
D10	*CD79A*	33.39	34.56	33.36	34.17	33.92	34.53	32.63	33.26	32.81	34.34	33.88	34.74
D11		31.58	35.25	33.32	34.33	32.16	34.14	33.01	32.97	31.37	34.4	32.99	34.84
D12		33.51	34.76	34.79	34.34	33.85	34.61	34.26	34.17	33.56	34.46	35.32	34.38
E1	*ZBTB7A*	25.35	25.59	24.96	25.52	25.15	25.09	25.55	25.24	25.12	25.72	25.61	26.13
E2		25.4	25.4	25.1	24.96	25.14	24.73	24.88	25.18	25.08	25.62	25.61	25.91
E3		25.41	25.44	25.19	25.02	24.99	24.71	24.82	24.79	25.12	25.54	25.64	25.86
E4	*TRAIL*	32.53	35.97	36.36	34.94	36.66	34.33	33.57	35.37	34.92	34.58	34.97	37.64
E5		33.66	36.45	36.62	36.89	37.24	37.75	37.76	36.13	34.91	36.14	36.59	37.53
E6		33.76	35.85	35.54	35.79	35.25	34.88	36.6	35.13	37.88	34.99	35.18	36.11
E7	*KLF3*	22.9	22.59	22.16	22.63	22.75	22.89	22.75	22.69	22.77	22.67	22.86	22.78
E8		22.87	22.51	22.17	22.64	22.75	22.95	22.77	22.72	22.81	22.71	22.84	22.77
E9		22.87	22.68	22.16	22.63	22.78	22.89	22.77	22.68	22.78	22.69	22.88	22.79
E10	*SMAD3*	24.65	25.9	26.94	25.48	24.81	24.18	25.81	24.9	25.23	25.71	26.12	25.46
E11		24.54	25.85	27.05	25.17	24.73	24.17	25.94	24.91	25.42	25.88	26.09	25.91
E12		24.62	26.31	27.2	25.91	25.21	24.48	26.13	25.45	25.82	26	26.47	26.16
F1	*GAPDH*	17.47	18.29	18.17	17.49	16.82	17.21	17.45	17.05	16.81	17.77	17.43	17.77
F2		17.48	18.43	18.22	17.69	16.91	17.17	17.49	17.03	16.88	17.65	17.1	17.78
F3		17.46	18.38	18.17	17.67	16.91	17.14	17.47	17.03	16.9	17.72	17.13	17.75

raw data		After erythroid differentiation											
		WT	-88	CD6	CD17	CD19	CD26	IVS-I-1	IVS-I-5	CD39	CD7172	IVS-II-1	IVS-II-745
well	Gene	Ct value	Ct value	Ct value	Ct value	Ct value	Ct value	Ct value	Ct value	Ct value	Ct value	Ct value	Ct value
A1	*HBA*	29.17	20.91	21.74	27.18	24.47	27.51	26.39	18.95	25	26.62	19.95	27.59
A2		28.96	20.84	21.88	27.3	24.12	27.21	26.49	19.13	24.76	26.64	19.98	28.75
A3		28.82	20.85	21.78	26.91	24.43	27.14	26.11	19.04	24.94	26.94	20.23	27.93
A4	*KLF1*	35.44	27.81	31.72	33.86	31.41	32.7	34.96	28.64	35.77	34.52	32.55	35.6
A5		34.22	28.02	31.73	35.03	31.87	33.06	35.05	29.64	34.26	34.43	31.26	36.29

A6		35.08	26.06	31.52	33.83	31.27	33.67	35.27	29.53	36.26	34.31	31.69	34.06
A7	*AHSP*	-	35.52	34.13	38.12	35.57	35.66	35.56	31.78	35.23	35.47	32.15	35.4
A8		-	34.84	35.38	38.05	35.33	35.93	35.55	31.62	35.79	35.54	32.83	35.32
A9		-	34.89	34.27	37.87	35.27	35.8	35.59	31.59	35.56	35.37	33.73	35.67
A10	*PRDX2*	34.38	23.58	26.46	32.27	29.22	31.94	30.78	21.52	29.22	32.13	24.37	33.42
A11		34.15	23.46	25.73	31.25	29.1	32.25	30.39	21.49	29.06	30.3	23.83	32.9
A12		34.36	23.56	25.79	31.92	28.72	33.25	30.39	21.53	29.11	30.98	23.89	30.53
B1	*HBB*	19.92	15.75	17.17	20.38	19.42	19.49	19.27	16.89	22.76	23.55	19.33	21.75
B2		19.94	15.82	17.13	20.46	19.39	19.42	19.3	16.87	22.78	23.5	19.21	21.74
B3		19.9	15.89	17.16	20.48	19.37	19.43	19.57	16.88	22.74	23.55	19.36	21.74
B4	*EPOR*	-	32.66	36.91	-	35.23	-	36.97	31.49	35.25	-	33.02	35.33
B5		-	31.58	36.43	-	35.23	-	37.59	30.63	35.57	-	34.42	35.43
B6		-	31.68	36.8	-	35.37	-	37.78	30.57	34.99	-	34.26	35.29
B7	*E2F2*	-	31.46	33.67	-	35.39	-	35.29	30	34.87	35.51	32.42	35.6
B8		-	30.18	32.89	-	34.61	-	35.38	29.86	35.64	34.98	32.29	35.59
B9		-	31.63	33.31	-	34.55	-	35.3	29.66	35.32	35.31	32.37	35.48
B10	*TWSG1*	-	33	35.77	38.01	34.97	36.21	-	31.61	35.36	-	33.93	-
B11		-	33.34	35.26	37	34.26	35.64	-	32.95	35.35	-	34.59	-
B12			32.62	36.12	37.77	34.77	35.23	-	33.52	36.05	-	33.77	-
C1	*HBG*	24.63	19.94	18.46	26.45	25.18	21.78	20.99	17.33	17.19	17.83	17.79	19.43
C2		24.81	20.04	18.57	26.49	24.96	21.73	21.35	17.14	17.17	17.89	17.88	19.69
C3		24.74	20.08	18.54	26.41	25.03	21.7	21.02	17.13	17.17	17.91	17.89	19.59
C4	*GF1B*	-	35.55	34.98	-	-	-	-	31.61	-	-	35.12	-
C5		-	35.25	35.48	-	-	-	-	32.24	-	-	34.84	-
C6		-	34.99	35.82	-	-	-	-	31.91	-	-	35.13	-
C7	*TAL1*	-	34.54	-	-	-	-	-	34.63	-	-	35.36	-
C8		-	35.15	-	-	-	-	-	35.38	-	-	35.4	-
C9		-	34.75	-	-	-	-	-	35.37	-	-	35.33	-
C10	*LYZ*	38.15	34.92	-	36.98	-	-	-	-	-	-	-	-
C11		37.95	35.75	-	37.33	-	-	-	-	-	-	-	-
C12		38.34	35.31	-	36.93	-	-	-	-	-	-	-	-
D1	*GAPDH*	30.89	23.51	24.79	29.23	26.76	28.53	28.11	22.48	25.51	26.58	22.65	29.69
D2		30.58	23.47	24.87	28.56	26.88	28.78	27.94	22.63	25.68	26.66	22.43	29.07
D3		30.7	23.45	24.79	28.63	26.82	28.52	27.9	22.4	25.59	26.59	22.57	28.97
D4	*C-MYB*	35.31	31.44	33.67	35.62	33.45	34.51	32.62	30.23	33.47	34.23	32.38	33.8

D5		36.82	31.03	34.42	35.05	35.22	34.27	33.88	29.96	34.18	34.3	31.87	33.78
D6		37.65	30.79	32.81	35.15	35.17	34.98	34.31	29.81	33.86	33.66	31.88	33.86
D7	*BCL-xL*	33.93	30.1	33.19	-	34.77	-	-	28.71	33	-	31.95	34.44
D8		34.46	30.11	33.32	-	34.63	-	-	28.22	34.49	-	31.08	34.5
D9		35.43	29.61	32.41	-	34.32	-	-	28.58	33.88	-	32.37	34.42
D10	*TUBB6*	38.22	33.64	34.64	-	-	38.72	-	32.58	-	36.14	33.31	-
D11		37.6	34.37	34.18	-	-	39.43	-	34.11	-	35.92	34.35	-
D12		37.1	33.79	36.15	-	-	39.32	-	33.62	-	36.01	35.43	-
E1	*BCL11A*	35.8	28.53	34.12	35.34	35.45	33.71	34.64	30.52	-	-	32.21	35.2
E2		35.79	29.06	33.33	34.89	34.64	33.62	33.57	30.51	-	-	32.82	35.58
E3		35.58	29.42	34.93	35.56	35.32	33.95	33.99	29.87	-	-	31.68	35.44
E4	*Band3*	35.02	27.95	33.29	35.03	34.16	33.87	35.83	27.16	35.93	35.75	29.92	36.14
E5		34.9	27.94	31.61	35.93	34.55	33.91	34.49	27.33	35.65	37.64	29.64	35.29
E6		33.52	27.98	31.04	35.88	34.26	33.95	34.21	27.2	33.64	36.89	29.9	34.46
E7	*HSP70*	33.79	28.9	30.75	34.82	33.94	34.72	34.95	27.69	33.34	34.59	28.51	35.01
E8		33.89	28.95	30.12	36.49	33.39	34.36	34.43	27.71	32.59	34.18	28.54	35.66
E9		36.18	29.17	30.18	34.77	34.82	35.83	36.66	27.67	33.93	33.38	28.78	36.03
E10	*UCHL1*	-	-	-	36.55	-	37.6	-	-	-	-	36.53	35.74
E11		-	-	-	36.64	-	36.82	-	-	-	-	37.01	35.8
E12		-	-	-	36.89	-	36.11	-	-	-	-	35.94	35.88
F1	*GATA1*	-	31.65	35.06	35.72	35.78	-	35.12	30.03	35.83	35.61	33.6	-
F2		-	33.1	34.83	35.25	36.44	-	35.61	30.08	35.38	37.56	33.7	-
F3		-	31.57	34.37	35.57	35.98	-	34.99	30.15	35.72	34.91	34.82	-
F4	*Band4.1*	35.63	27.91	30.92	35.05	33.91	33.34	32.61	27.63	32.97	32.66	29.24	35.17
F5		35.75	27.94	30.33	35.74	33.91	32.16	34.84	27.66	32.24	32.84	29.77	33.33
F6		36.18	27.94	30.03	33.91	33.51	32.55	33.5	27.76	31.97	32.55	29.54	36.3
F7	*Caspase-3*	34.94	28.76	31.27	34.44	33.6	33.84	34.21	28.1	33.56	33.25	29.4	35.23
F8		35.2	28.7	30.8	33.95	33.54	34.42	34.33	27.97	33.27	34.16	29.46	35.18
F9		35.33	28.55	31.97	34.52	33.31	35.2	33.91	27.83	33.58	32.94	29.69	34.39
F10	*SMAD1*	35.98	35.69	36.27	-	36.45	36.54	-	-	36.01	36.79	-	36.9
F11		36.22	36.61	36.52	-	36.92	37.29	-	-	37.39	36.74	-	34.34
F12		36.57	36.48	36.58	-	36.78	35.86	-	-	36.89	36.82	-	37.36
G1	*GATA2*	37.21	31.52	35.25	35.13	36.12	35.57	34.12	33.04	-	36.94	34.76	-
G2		37.28	32.65	35.27	35.87	35.71	35.43	34.31	33.01	-	36.93	34.88	-
G3		37.11	32.36	36.19	35.45	35.89	34.92	34.25	32.76	-	34.49	32.85	-

G4	*Ank1*	32.75	26.09	28.71	34.47	31.27	32.21	33.84	21.13	25.88	30.87	27.42	29.82
G5		32.95	26.1	28.6	32.73	30.86	31.26	33.25	21	25.8	30.25	27.55	30.07
G6		31.95	26	28.53	31.53	31.97	31.28	33.5	21.28	24.62	28.59	27.12	30.33
G7	*JAK2*	33.78	27.78	28.48	32.11	31.11	30.95	33.26	27.38	30.23	32.46	27.95	33.72
G8		34.15	27.78	28.13	30.93	32.11	32.7	32.82	26.93	30.3	32.15	27.82	35.1
G9		34.28	28.13	28.85	32.5	32.39	31.46	32.38	27.58	29.72	30.14	27.9	35.1
G10	*TGFB3*	-	-	-	-	-	-	-	-	-	35.33	-	-
G11		-	-	-	-	-	-	-	-	-	35.04	-	-
G12		-	-	-	-	-	-	-	-	-	34.96	-	-
H1	*GYPA*	28.04	21.89	23.14	26.66	26.25	25.97	25.69	20.64	21.52	21.44	22.56	26.53
H2		28.13	22.1	23.12	26.54	25.93	25.96	25.75	20.72	21.48	21.51	22.52	26.45
H3		27.87	22.11	23.1	26.65	25.84	25.91	25.67	20.38	21.52	21.57	22.54	26.42
H4	*SPTA1*	34.02	26.68	28.87	34.82	32.72	34.52	34.58	25.76	34.32	34.8	27.19	-
H5		34.04	26.51	29.91	33.54	32.5	34.5	35.41	25.72	32.52	34.37	27.38	-
H6		33.97	26.61	29.57	32.84	32.88	35.45	34.93	25.63	27.61	34.13	27.39	-
H7	*FAS-L*	35.35	27.26	27.41	29.42	34.29	32.1	34.91	32.1	29.47	28.69	35.27	34.51
H8		34.43	26.95	27.35	30.28	37.62	31.91	33.75	29.58	33.41	29.05	34.8	34.95
H9		34.07	26.96	27.59	31.02	31.63	32.11	32.61	28.76	31.49	28.82	35.07	35.1
H10	*STAT5*	37.89	33.61	34.23	33.12	34.92	34.56	-	33.45	34.98	35.12	35.34	35.74
H11		37.67	32.27	33.98	33.3	34.69	35.37	-	34.69	35.7	35.68	34.11	34.98
H12		36.11	31.04	34.45	30.21	35.12	34.57	-	32.64	36.97	35.32	36.13	35.99
A1	*Caspase-9*	36.33	35.84	37.34	37.23	36.39	35.64	36.25	34.2	37.43	37.54	35.55	37.33
A2		36.19	36.12	37.23	37.29	36.35	35.65	36.8	34.13	37.4	35.71	35.6	37.36
A3		36.22	35.9	37.32	37.35	36.29	35.6	36.27	33.98	37.29	37.16	35.56	37.29
A4	*Cyclin-A*	35.57	29.83	29.72	33.63	33.01	31.51	30.94	26.14	32.9	34.37	27.85	34.2
A5		35.39	29.46	29.36	34.43	32.9	31.61	30.86	26.14	32.66	33.83	28.24	32.94
A6		35.47	29.4	29.66	34.42	32.99	32.51	31.49	26.14	32.67	33.73	28.2	35.01
A7	*SOX6*	37.47	28.67	32.6	34.62	36.44	32.42	34.47	27.93	34.64	34.77	30.96	34.86
A8		35.16	28.67	31.52	32.74	35.04	32.38	36.47	27.95	34.12	33.07	29.77	33.86
A9		34.39	28.21	32.15	33.53	33.67	32.78	36.19	27.74	35.88	33.59	30.86	35.51
A10	*TNF*	37.13	37.13	37.39	36.77	36.58	37.11	37.34	37.35	37.35	37.36	37.42	37.63
A11		37.23	37.33	37.31	36.93	36.53	37.31	37.3	37.34	37.38	37.39	37.3	37.22
A12		37.21	37.45	37.12	37.52	36.49	37.2	37.29	37.4	37.34	37.35	37.35	37.3
B1	*NFE4*	37.34	34.92	35.14	37.54	37.43	34.63	35.12	35.29	34.93	37.35	34.39	34.73

B2		37.27	36.02	35.57	37.27	37.82	34.14	35.6	35.35	35.56	37.2	34.54	35.55
B3		37.42	35.02	34.67	37.44	37.55	34.44	35.93	35.23	35.27	37.28	34.21	34.89
B4	*CDHBP*	32.94	28.57	28.93	31.02	30.66	28.98	29.12	28.59	30.03	29.4	27.55	29.18
B5		33.65	28.38	28.86	30.82	30.35	31.25	30.71	28.37	30.29	28.93	27.76	29.25
B6		32.81	28.19	28.91	30.11	31.14	30.11	29.91	28.44	31.2	29.19	27.51	28.37
B7	*AKT*	35.99	33.43	36.91	34.98	37.82	37.29	37.26	31.65	37.79	35.14	38.14	35.9
B8		36.25	33.93	37.12	34.73	37.13	37.36	37.24	32.95	37.97	37.1	34.92	35.63
B9		37.43	33.67	37.09	34.84	37.27	37.33	37.28	33.41	37.88	36.59	34.89	35.81
B10	*TFR1*	31.26	23.1	27.12	29.64	28.7	29.14	28.95	22.52	27.99	29.13	25.03	30.01
B11		31.48	23.04	26.99	29.76	28.75	28.88	29.55	22.59	28.68	29.2	25.45	29.82
B12		31.53	23.39	27.6	30.67	29.2	29.59	29.63	22.78	28.62	30.36	25.75	30.88
C1	*NFE2*	33.69	26.93	28.82	33.37	32.65	33.31	33.71	25.21	31.36	33.63	27.5	33.29
C2		34.68	26.52	28.98	34.24	31.65	32.9	32.98	25.42	32.11	32.98	27.52	34.63
C3		34.44	26.84	29.1	33.29	33.62	33.66	33.73	25.37	33.32	33.5	27.33	33.76
C4	*SMAD2*	35.58	28.55	30.8	34.38	32.87	35.44	32.39	28.43	33.67	35.5	29.47	35.51
C5		35.53	28.65	31.36	35.17	34.18	34.83	33.8	28.5	35.35	35.55	29.45	35.57
C6		35.51	28.7	31.03	35.63	33.56	35.4	33.59	28.37	33.98	33.92	29.26	34.84
C7	*CDK2*	35.32	34.9	36.43	37.54	37.78	36.13	35.04	31.4	36.88	35.89	35.25	34.34
C8		34.76	35.41	35.28	34.9	36.76	36.14	36.17	31.15	37.53	35.34	34.3	33
C9		35.3	37.73	36.71	37.67	35.48	37.19	36.7	31.49	35.13	34.67	34.87	36.27
C10	*GDF11*	37.7	35.89	34.59	35.67	37.64	37.58	34.7	37.02	33.51	34.15	32.74	35.58
C11		37.31	35.88	32.89	35.44	37.79	37.35	33.9	37.66	35.3	35.85	33.7	34.69
C12		37.33	35.85	35.81	33.77	35.26	35.43	35.66	34.89	34.49	34.46	34.68	33.7
D1	*LYAR*	35.15	30.83	31.11	33.27	33.95	33.56	31.7	28.73	32.82	33.33	29.74	32.65
D2		34.75	30.34	31.48	33.6	34.23	32.52	31.45	28.72	33.33	32.33	29.45	32.64
D3		33.65	29.96	30.12	33.38	33.69	33.59	32.46	28.15	32.35	32.86	29.9	33.56
D4	*Caspase-8*	36.39	34.2	35.11	35.01	35.08	34.87	34.8	34.2	36.62	32.84	33.34	35.16
D5		36.23	32.8	35.32	37.63	34.57	34.59	35.07	33.3	34.58	34.91	32.51	35.58
D6		36.37	33.45	37.32	34.9	34.26	33.9	34.65	34.28	34.45	35.55	33.31	37.01
D7	*GDF15*	36.93	37.45	36.02	35.99	36.89	37.25	37.59	35.36	36.74	37.29	37.78	37.33
D8		37.2	37.53	34.52	35.97	36.81	35.09	37.88	37.64	37.14	37.2	37.15	37.4
D9		37.59	37.39	36.04	36.13	36.93	37.68	37.91	37.04	36.85	37.43	37.42	37.35
D10	*CD79A*	37.27	35.67	37.02	37.57	36.97	37.39	35.61	37.32	37.28	37.57	35.12	34.29
D11		37.39	35.12	37.13	37.42	37.02	37.42	36.7	37.33	37.29	37.7	35.1	34.21
D12		37.84	36.42	37.09	34.49	37.42	37.4	37.82	37.47	37.3	38.11	35.11	34.24

E1	*ZBTB7A*	35.19	31.52	35.15	37.2	34.51	34.3	33.68	30.95	35.32	37.29	31.6	35.3
E2		35.11	30.94	34.21	37.13	35.16	34.43	33.76	30.03	34.9	37.5	31.04	35.29
E3		35.07	32.1	33.75	37.3	34.3	34.24	33.82	30.36	34.32	37.38	31.91	35.26
E4	*TRAIL*	37.87	37.35	36.94	35.78	37.34	37.53	32.15	35.77	35.16	37.71	35.96	33.88
E5		37.39	36.79	36.93	35.94	37.43	37.6	34.4	35.82	35.65	37.5	36.16	35.15
E6		37.45	35.82	37.2	35.86	37.31	37.02	34.75	35.69	35.32	37.46	36.02	34.5
E7	*KLF3*	35.28	27.71	29.93	32.99	29.66	32.53	31.22	26.89	32.93	29.7	25.91	33.61
E8		34.66	27.62	28.63	29.15	30.62	32.99	32.6	26.59	32.34	30.88	27.78	30.06
E9		33.53	27.59	29.64	33.66	30.22	29.05	30.73	26.91	30.8	29.6	27.68	27.78
E10	*SMAD3*	37.55	32.34	35.04	34.65	36.12	36.3	33.42	34.95	36.58	32.69	33.72	36.88
E11		37.64	33.78	36.17	34.65	35.21	34.56	33.49	34.48	34.99	33.79	33.36	36.87
E12		37.52	33.37	35.18	34.67	35.82	35.14	33.45	34.86	35.66	35.47	32.96	36.83
F1	*GAPDH*	29.86	22.99	24.64	27.84	26.54	28.14	27.55	22.05	25.31	26.32	22.13	28.72
F2		29.74	22.96	24.58	27.79	26.51	28	27.42	22.05	25.25	26.33	22.15	28.52
F3		30.17	22.88	24.51	27.85	26.54	28.04	27.35	21.98	25.21	26.33	22.33	28.21
F4	*AHSP*	37.2											
F5		37.48											
F6		37.35											
F7	*EPOR*	36.42			35.56		35.12				37.53		
F8		36.43			35.76		35.13				37.28		
F9		36.19			34.91		35.1				37.31		
F10	*E2F2*	35.09			34.43		35.45						
F11		34.92			35.14		35.46						
F12		34.89			35.23		35.46						
G1	*TWSG1*	37.44						36.67			37.27		37.28
G2		37.65						36.64			37.3		37.31
G3		37.39						36.59			37.25		37.39
G4	*GF1B*	37.53			36.88	37.23	36.56	37.86		37.1	37.19		34.99
G5		37.43			37.11	37.59	36.54	37.52		37.08	37.15		35.18
G6		37.19			37.5	37.64	36.51	37.93		37.22	37.45		34.95
G7	*TAL1*	37.69		35.52	36.97	37.74	37.39	37.5		37.69	37.53		37.34
G8		37.53		35.54	37.22	37.7	37.74	37.48		37.77	37.27		37.29
G9		37.67		35.51	37.5	37.34	37.58	37.61		37.68	37.43		37.4
G10	*LYZ*			37.2		37.54	37.47	37.38	37.39	37.43	37.29	37.2	37.43
G11				37.12		37.92	37.44	37.39	37.28	37.39	37.37	37.18	37.3

G12				37.1		37.5	37.43	37.7	37.19	37.4	37.35	37.24	37.32
H1	*BCL-xL*				34.33		34.95	34.45			34.79		
H2					34.42		34.97	34.89			35.7		
H3					34.47		34.95	35.36			37.01		
H4	*TUBB6*				35.57	37.49		37.55		37.54			35.59
H5					35.54	37.76		37.53		37.19			35.62
H6					35.58	37.23		37.55		37.38			35.5
H7	*BCL11A*									35.45	37.58		
H8										35.47	35.5		
H9										35.45	35.98		
H10	*UCHL1*	35.56	37.13	37.22		36.7		35.21	36.92	36.24	37.75		
H11		36.69	37.32	37.57		35.44		35.26	35.89	33.46	37.25		
H12		35.92	37.2	35.86		36.75		35.27	36.75	36.74	37.26		
H13	*GATA1*	34.79					35.55						35.89
H14		35.28					35.61						35.39
H15		35.35					35.49						36.49
H16	*SMAD1*				37.42			34.53	37.13			37.49	
H17					37.48			34.59	37.43			37.76	
H18					37.41			34.54	37.34			37.36	
H19	*GATA2*									35.43			35.13
H20										35.48			37.21
H21										35.46			36.86
H22	*TGFB3*	36.79	37.22	35.96	36.5	37.23	37.34	37.43	37.37	37.29		35.38	37.4
H23		37.43	37.39	35.74	36.52	37.19	37.2	37.56	37.35	37.4		35.43	37.52
H24		37.39	37.29	36.3	36.6	37.34	37.32	37.5	37.43	37.38		35.4	37.45
H25	*SPTA1*												35.13
H26													33.65
H27													34.6
H28	*STAT5*							34.36					
H29								31.81					
H30								34.39					

Logarithm values for qPCR

logarithm value	Before erythroid differentiation											
	WT	-88	CD6	CD17	CD19	CD26	IVS-I-1	IVS-I-5	CD39	CD7172	IVS-II-1	IVS-II-745

Gene	value	value	value	value	value	value	value	value	value	value	value	value
HBA	-0.303	1.356	2.431	1.266	0.016	-1.084	1.345	0.222	-0.134	1.405	1.144	1.645
	0.3	1.599	2.743	1.259	0.113	-0.883	0.652	0.444	0.323	1.53	1.005	2.054
	0.002	1.654	2.639	1.114	0.127	-1.146	0.499	0.457	0.081	1.419	0.818	1.652
KLF1	0.007	0.196	0.98	0.328	-0.333	-0.275	-0.051	-0.501	-0.483	0.092	-0.55	0.423
	-0.021	0.245	0.924	0.598	-0.159	-0.053	0.185	-0.293	-0.351	0.106	-0.287	0.444
	0.014	0.356	1.368	0.682	-0.159	0.051	0.268	-0.245	-0.254	0.287	0.081	0.52
AHSP	-0.021	1	1.694	0.397	-0.825	-1.446	0.282	-0.023	-0.337	0.349	1.308	0.658
	-0.042	0.661	1.312	0.439	-0.894	-0.795	0.531	0.005	-0.178	0.481	1.412	0.679
	0.062	0.723	1.881	0.536	-0.381	-0.684	0.594	-0.03	-0.233	0.515	1.335	0.326
PRDX2	-0.065	0.894	1.054	0.756	-0.106	-0.756	0.404	0.044	-0.007	0.672	0.238	0.129
	0.005	0.894	1.469	0.658	-0.079	-0.728	0.432	0.037	-0.104	0.541	0.349	0.427
	0.06	0.908	1.539	0.665	-0.113	-0.749	0.377	0.127	-0.152	0.52	0.307	0.427
HBB	0.104	0.883	0.39	0.446	-1.026	-0.809	-2.685	-2.47	-4.046	-2.382	-2.858	-2.786
	-0.035	0.876	0.273	0.307	-0.984	-0.628	-2.775	-2.262	-4.046	-2.389	-3.087	-2.904
	-0.069	0.876	0.293	0.363	-0.998	-0.628	-2.782	-2.269	-4.108	-2.396	-3.073	-2.918
EPOR	-0.109	0.573	0.892	-0.002	-0.788	-0.73	-0.229	-0.527	-1.063	-0.224	-0.957	-0.323
	-0.067	0.691	1.225	0.095	-0.698	-0.723	-0.146	-0.388	-1	-0.127	-0.977	-0.129
	0.176	0.906	1.46	0.247	-0.254	-0.446	0.125	-0.028	-0.709	0.122	-0.374	0.113
E2F2	0.072	1.751	1.828	0.816	-0.171	-0.37	0.132	-0.229	-0.619	0.767	-0.062	0.633
	-0.032	1.731	1.807	0.725	-0.33	-0.481	0.042	-0.36	-0.515	0.705	0	0.488
	-0.039	1.661	1.571	0.691	-0.358	-0.363	0.028	-0.416	-0.605	0.705	-0.049	0.488
TWSG1	0.164	0.291	0.416	-0.236	-0.023	0.014	-0.025	-0.164	-0.381	-0.007	-0.393	-0.217
	0.157	0.298	0.277	-0.062	-0.051	0.069	0.12	-0.213	-0.049	0.007	-0.379	-0.224
	-0.321	0.27	0.402	-0.464	-0.009	-0.021	-0.441	-0.531	-0.159	-0.381	-0.552	-0.598
HBG	-0.129	1.01	2.202	3.616	-0.92	-1.451	1.365	0.665	5.654	2.5	3.362	4.189
	0.051	0.947	2.133	3.547	-0.913	-1.472	1.289	0.95	5.654	2.5	3.369	4.279
	0.079	1.01	2.084	3.533	-0.906	-1.458	1.296	0.915	5.612	2.479	3.327	4.279
GF1B	-0.014	0.488	0.73	-0.018	0.097	-0.524	-0.217	-0.557	-0.573	-0.365	-0.488	-0.756
	0.049	0.501	0.827	0.356	0.243	-0.511	-0.287	-0.453	-0.739	-0.317	-0.474	-0.686
	-0.035	0.467	0.841	0.342	0.125	-0.386	-0.169	-0.474	-0.379	-0.268	-0.293	-0.444
TAL1	0.044	0.913	1.363	0.642	-0.337	0.33	0.506	-0.173	-0.702	0.677	-0.381	0.314
	-0.06	0.76	0.774	0.31	-0.524	-0.12	0.194	0.069	-0.994	0.719	-1.359	-0.012
	0.016	0.795	1.356	0.76	-0.032	0.414	0.568	-0.388	-0.938	0.732	-0.624	0.321
LYZ	0.559	-0.853	0.395	-1.005	-1.77	0.499	0.231	-1.37	-0.236	-0.34	-1.765	-0.134

	0.206	-0.936	-0.256	-0.742	-2.123	1.407	-0.233	-1.349	-0.104	-0.631	-1.862	0.303
	-0.765	-0.534	-0.166	-0.284	-1.548	0.61	-0.524	-1.273	-0.319	-1.262	-1.821	-0.418
C-MYB	-0.005	-0.488	-0.494	-0.488	-0.24	0.012	-0.229	-0.402	-0.404	-0.813	-0.832	-0.802
	-0.012	-0.543	-0.342	-0.474	-0.226	0.06	-0.208	-0.416	-0.404	-0.786	-0.86	-0.753
	0.016	-0.481	-0.342	-0.446	-0.226	0.032	-0.187	-0.374	-0.397	-0.786	-0.818	-0.753
BCL-xL	0.014	0.98	1.077	0.432	-0.152	-0.254	0.046	-0.446	-0.608	0.134	-0.307	-0.049
	0	0.869	1.07	0.411	-0.159	-0.102	-0.023	-0.446	-0.594	0.134	-0.55	0.028
	-0.014	0.841	1.028	0.3	-0.139	-0.018	0.046	-0.488	-0.462	0.127	-0.349	-0.007
TUBB6	0.141	0.559	0.698	0.081	-0.164	-1.506	0.173	-0.173	-0.737	-0.044	-0.208	0.155
	0.155	0.615	0.725	0.053	-0.136	-0.259	0.062	-0.284	-0.446	0.012	-0.291	0.072
	-0.296	-0.016	0.282	0.039	-0.628	-0.03	-0.166	-0.541	-0.896	-0.314	-0.541	-0.178
BCL11A	-0.146	1.007	0.467	0.293	0.014	0.002	-0.072	-0.113	-1.523	-0.414	-0.418	-1.518
	-0.014	0.959	0.716	0.002	0.035	-0.116	-0.113	-0.002	-1.647	-0.421	-0.571	-1.026
	0.159	1	0.751	0.085	0.055	-0.039	-0.113	-0.002	-1.55	-0.448	-0.418	-1.067
Band3	0.007	1.846	2.512	1.583	-0.381	-1.571	0.947	-0.016	-0.185	1.375	0.483	1.192
	0.014	1.846	2.512	1.583	-0.395	-1.53	0.927	-0.016	-0.206	1.382	0.476	1.164
	-0.021	1.874	2.574	1.666	-0.354	-1.536	0.927	-0.009	-0.192	1.375	0.538	1.192
HSP70	0.014	0.037	0.245	-0.032	-0.152	-1.488	-0.162	-0.21	-0.226	-0.032	-0.266	-0.194
	-0.007	-0.039	0.21	0.002	-0.09	-0.247	-0.196	-0.176	-0.268	-0.116	-0.307	-0.215
	-0.007	-0.06	0.12	-0.039	-0.166	-0.122	-0.155	-0.273	-0.233	-0.025	-0.287	-0.18
UCHL1	0.095	-5.199	-2.696	0.263	1.273	-6.169	-2.146	-1.058	0.187	-1.774	-0.261	-2.165
	0.372	-3.175	-5.531	0.125	1.301	-3.764	-3.054	-1.682	0.374	-2.114	-0.635	-2.463
	-0.467	-4.159	-3.098	0.021	0.476	-4.429	-2.847	-1.911	0.201	-3.154	-0.906	-2.636
GATA1	-0.012	0.524	0.85	0.358	-0.122	0.226	0.159	-0.589	-0.689	-0.127	-0.451	-0.462
	-0.005	0.725	0.823	0.199	-0.06	0.282	0.159	-0.582	-0.682	0.053	-0.471	-0.247
	0.016	0.608	0.85	0.296	-0.046	0.254	0.166	-0.561	-0.702	0.095	-0.464	-0.261
Band4.1	0	0.91	1.042	0.481	-0.173	-0.032	0.06	-0.446	-0.545	0.217	-0.46	0.021
	0.007	0.917	1.021	0.481	-0.146	-0.032	0.046	-0.467	-0.545	0.21	-0.418	0.028
	-0.007	0.917	1.07	0.474	-0.152	-0.039	0.074	-0.432	-0.524	0.335	-0.39	0.007
Caspase-3	-0.102	0.032	0.088	-0.328	-0.608	-0.072	-0.243	-0.27	-0.709	-0.363	-0.665	-0.386
	0.037	0.053	0.102	-0.134	-0.552	-0.065	-0.256	-0.263	-0.467	-0.384	-0.652	-0.365
	0.065	0.046	0.074	-0.231	-0.559	-0.079	-0.284	-0.277	-0.619	-0.384	-0.617	-0.386
SMAD1	0.062	-1.904	-2.625	1.818	-3.119	-4.281	-2.9	-2.969	-4.073	-2.916	-2.47	-2.738
	0.18	-2.188	-3.041	1.749	-2.613	-3.484	-3.101	-3.177	-5.91	-2.389	-2.636	-2.696
	-0.243	-3.609	-3.103	1.714	-2.53	-5.203	-2.99	-3.038	-2.937	-2.368	-2.851	-3.147

GATA2	0.007	-0.421	0.189	-0.601	0.229	-0.095	0.171	0.06	-0.622	-0.719	-0.501	-0.367
	-0.007	-0.462	0.141	-0.913	0.243	-0.157	0.275	0.053	-0.705	-0.781	-0.536	-0.471
	0	-0.615	0.106	-0.809	0.263	-0.171	0.317	0.081	-0.649	-0.788	-0.522	-0.444
Ank1	-0.037	0.839	1.061	0.451	-0.072	-0.007	0.127	-0.247	-0.333	0.367	-0.289	0.15
	0.06	0.929	1.088	0.499	-0.051	0.007	0.162	-0.213	-0.333	0.437	-0.414	0.136
	-0.023	0.86	1.109	0.548	-0.065	0.028	0.134	-0.226	-0.354	0.43	-0.268	0.213
JAK2	-0.067	0.885	0.996	0.393	-0.143	-0.259	0.021	-0.132	-0.446	0.275	-0.111	0.106
	0.085	0.913	0.982	0.386	-0.109	-0.238	-0.111	-0.09	-0.397	0.317	-0.049	0.196
	-0.018	0.94	0.816	0.414	-0.018	-0.245	0.035	-0.159	-0.425	0.31	-0.055	0.106
TGFB3	0.116	-0.083	-0.132	-0.312	-0.259	0.25	0.321	-0.386	-0.922	0.187	-0.698	-0.73
	0.012	0.042	-0.042	-0.208	-0.148	0.284	0.377	-0.317	-0.804	-0.139	-0.802	-0.675
	-0.127	-0.222	-0.388	-0.693	-0.155	-0.132	0.231	-0.622	-1.532	-0.423	-1.183	-1.091
GYPA	0.035	1.16	1.791	0.869	0.139	-0.421	0.171	0.039	-0.143	1.007	1.086	0.679
	-0.021	1.174	1.77	0.827	0.194	-0.531	0.289	0.081	-0.393	0.786	1.093	0.658
	-0.014	1.181	1.756	0.515	0.166	-0.407	0.171	0.088	-0.33	0.924	0.656	0.679
SPTA1	0.009	0.829	0.975	-0.231	-0.323	-0.141	-0.18	-0.173	-0.806	0.046	-0.582	0.134
	-0.005	0.85	0.975	-0.189	-0.303	-0.134	-0.146	-0.187	-0.82	-0.002	-0.444	0.155
	-0.005	0.864	0.947	-0.016	-0.261	-0.155	-0.159	-0.173	-0.626	-0.141	-0.215	0.134
FAS-L	-0.042	1.992	2.941	5.444	1.463	3.496	3.859	1.675	0.002	0.009	0.504	0.132
	0.021	0.654	4.244	4.75	1.65	2.796	1.696	-0.058	0.051	0.453	0.933	0.714
	0.021	0.716	4.445	5.444	0.88	1.777	2.68	-0.141	0.203	0.876	0.06	0.513
STAT5	0.012	-0.61	-0.395	-0.485	-0.55	0.152	-0.074	-0.67	-0.887	-0.95	-1.363	-0.384
	-0.009	-0.61	-0.367	-0.222	-0.55	0.18	0.03	-0.49	-0.783	-0.846	-1.439	-0.37
	-0.002	-0.638	-0.374	-0.464	-0.571	0.173	-0.067	-0.49	-0.783	-0.721	-1.294	-0.411
Caspase-9	-0.039	0.596	0.568	0.076	-0.462	0.24	0.169	-0.672	-0.737	0.053	-0.829	0.125
	-0.109	0.714	0.416	-0.049	-0.455	0.136	-0.129	-0.471	-0.571	-0.293	-0.386	-0.083
	0.148	0.555	0.256	-0.423	-0.365	0.032	-0.469	-1.074	-0.848	-0.217	-0.601	-0.125
Cyclin-A	0.113	0.457	0.707	0.097	-0.344	-0.287	-0.067	-0.021	-0.037	0.365	0.148	0.478
	-0.06	0.222	0.707	0.09	-0.365	-0.37	-0.095	0.125	-0.134	0.407	0.092	0.243
	-0.053	0.27	0.686	0	-0.31	-0.37	-0.046	0.104	-0.03	0.317	0.176	0.291
SOX6	-0.095	1.969	2.502	0.658	-0.005	-0.585	0.786	0.416	-0.931	0.282	0.709	-0.395
	0.148	2.253	2.544	0.776	0.079	-0.89	1.014	0.367	-0.966	0.538	0.654	-0.222
	-0.053	2.218	2.731	0.783	0.155	-0.744	0.91	0.381	-0.924	0.476	0.64	-0.277
TNF	-0.684	-1.615	-0.326	-2.052	-2.708	-1.305	-2.292	-1.102	2.022	-2.116	-2.576	-0.714
	0.293	-0.478	-0.305	-2.294	-2.847	-0.162	-1.959	-2.1	2.424	-2.241	-2.618	-0.194

	0.39	0.09	-1.241	-0.86	-2.86	-0.432	-0.767	-0.679	2.451	-1.007	-1.356	-0.34
NFE4	-0.104	0.663	-0.058	-1.063	-0.769	-1.335	0.485	0.213	-0.247	-1.273	-0.783	-0.924
	0.021	0.566	-0.009	-1.139	-1.067	-0.802	0.686	1.516	-1.01	-1.391	-1.546	0.102
	0.083	0.712	-0.092	-1.028	-1.497	-1.252	-0.437	0.206	-1.114	-1.252	-1.289	-1.444
CDHBP	0.037	2.274	-0.776	-0.104	0.439	-0.127	-2.015	0.52	0.351	0.421	0.744	0.395
	-0.109	2.336	-0.832	-0.471	0.321	-0.044	-2.313	0.395	0.393	0.455	0.661	0.617
	0.072	2.537	-0.922	-0.236	0.39	-0.092	-2.25	0.437	0.483	0.566	0.64	0.499
AKT	0.3	0.596	0.347	-0.049	0.259	0.421	0.626	-0.021	-0.217	0.219	-0.323	0.159
	-0.088	0.388	-0.076	-0.291	0.079	0.15	0.529	-0.333	-0.633	-0.314	-0.573	-0.277
	-0.213	-0.028	0	-0.464	-0.129	0.005	0.245	-0.284	-0.661	-0.238	-0.746	-0.354
TFR1	-0.032	0.589	0.797	0.028	-0.261	-0.259	-0.746	-0.527	-0.46	-0.266	-0.518	-0.34
	0.002	0.575	0.776	0.34	-0.358	-0.252	-0.753	-0.52	-0.46	-0.273	-0.538	-0.354
	0.03	0.617	0.714	0.166	-0.31	-0.273	-0.968	-0.541	-0.494	-0.578	-0.732	-0.457
NFE2	0.002	0.541	0.95	0.166	-0.386	-0.002	-0.122	-0.513	-0.557	0.088	-0.261	-0.104
	0.044	0.617	1.005	0.354	-0.33	0.06	0.03	-0.478	-0.481	0.088	-0.185	-0.062
	-0.046	0.631	1.033	0.347	-0.386	0.039	0.016	-0.492	-0.488	0.088	-0.199	-0.083
SMAD2	-0.005	0.444	0.388	0.069	-0.282	-0.079	-0.33	-0.173	-0.564	0.15	-0.206	0.076
	0.002	0.416	0.388	0.111	-0.31	-0.072	-0.33	-0.194	-0.55	0.143	-0.24	0.069
	0.002	0.437	0.423	0.139	-0.282	-0.072	-0.337	-0.173	-0.536	0.136	-0.213	0.097
CDK2	0.055	0.504	0.559	0.178	-0.291	-0.053	-0.09	-0.21	-0.531	0.189	-0.464	0.116
	-0.007	0.372	0.511	0.116	-0.27	-0.06	-0.118	-0.252	-0.504	0.196	-0.451	0.136
	-0.049	0.504	0.483	0.081	-0.374	-0.157	-0.118	-0.259	-0.476	0.085	-0.444	-0.016
GDF11	0.187	-1.728	-0.654	-0.626	-0.326	0.654	-0.284	-0.134	-0.247	-0.76	-1.296	-0.744
	-0.118	-1.056	-0.689	-0.702	-0.25	0.543	-0.028	-0.12	-0.226	-1.003	-1.033	-0.73
	-0.069	-1.86	-1.07	-1.042	-0.485	0.342	-0.728	-0.314	-0.427	-1.058	-1.788	-1.042
LYAR	-0.18	-0.148	-0.328	-0.155	-0.132	-0.289	-0.402	-0.266	-0.088	-0.206	0.076	-0.529
	0.083	0.018	-0.12	0.081	-0.208	-0.067	0.09	-0.079	0.148	-0.102	0.25	-0.148
	0.097	0.074	-0.079	0.074	-0.18	-0.088	0.097	-0.106	0.127	-0.039	0.222	-0.196
Caspase-8	-0.042	-0.072	0.067	-0.349	-0.485	-0.025	0.076	-0.564	-0.441	-0.046	-0.735	-0.605
	0.014	0.005	-0.03	-0.411	-0.457	-0.018	0.069	-0.564	-0.476	-0.081	-0.624	-0.64
	0.028	0.122	-0.016	-0.356	-0.437	-0.046	-0.028	-0.501	-0.524	-0.018	-0.645	-0.522
GDF15	1.012	3.082	3.013	2.632	1.657	1.312	0.187	0.982	0.252	1.181	-0.846	0.975
	-0.894	2.736	3.138	0.594	0.977	1.811	0.762	-0.051	1.61	0.987	1.4	1.197
	-0.118	2.632	3.054	0.261	0.596	1.167	2.128	0.705	0.432	1.915	0.34	0.795
CD79A	-0.39	-0.58	0.127	-0.829	-1.167	-1.386	0.136	-0.601	-0.409	-0.88	-0.903	-1.121

	0.864	-1.058	0.155	-0.94	0.053	-1.116	-0.127	-0.4	0.589	-0.922	-0.287	-1.19
	-0.474	-0.719	-0.864	-0.947	-1.118	-1.442	-0.994	-1.231	-0.929	-0.963	-1.902	-0.871
ZBTB7A	0.025	0.481	0.792	0.009	-0.245	0	-0.113	-0.199	-0.236	-0.062	-0.328	-0.31
	-0.009	0.612	0.695	0.397	-0.238	0.25	0.351	-0.157	-0.208	0.007	-0.328	-0.157
	-0.016	0.585	0.633	0.356	-0.134	0.263	0.393	0.113	-0.236	0.062	-0.349	-0.122
TRAIL	0.545	-1.218	-1.613	-1.024	-2.726	-0.908	-0.176	-1.724	-1.532	-0.707	-1.319	-2.791
	-0.238	-1.55	-1.793	-2.375	-3.128	-3.279	-3.08	-2.25	-1.525	-1.788	-2.442	-2.715
	-0.307	-1.134	-1.044	-1.613	-1.749	-1.289	-2.276	-1.557	-3.584	-0.991	-1.465	-1.731
KLF3	-0.014	0.823	0.996	0.275	-0.319	-0.213	0.09	-0.169	-0.344	0.314	-0.159	0.275
	0.007	0.878	0.989	0.268	-0.319	-0.254	0.076	-0.189	-0.372	0.287	-0.146	0.282
	0.007	0.76	0.996	0.275	-0.34	-0.213	0.076	-0.162	-0.351	0.3	-0.173	0.268
SMAD3	-0.032	-0.277	-1.123	-0.506	-0.552	0.088	-0.836	-0.506	-0.855	-0.598	-1.225	-0.388
	0.044	-0.243	-1.199	-0.291	-0.497	0.095	-0.927	-0.513	-0.987	-0.716	-1.204	-0.7
	-0.012	-0.561	-1.303	-0.804	-0.829	-0.12	-1.058	-0.887	-1.264	-0.799	-1.467	-0.873

logarithm value	After erythroid differentiation											
	WT	-88	CD6	CD17	CD19	CD26	IVS-I-1	IVS-I-5	CD39	CD7172	IVS-II-1	IVS-II-745
Gene	value	value	value	value	value	value	value	value	value	value	value	value
HBA	-0.129	0.573	0.927	-0.079	0.423	-0.444	-0.102	1.257	-0.795	-1.213	0.596	-0.06
	0.016	0.622	0.829	-0.162	0.665	-0.236	-0.171	1.132	-0.628	-1.227	0.575	-0.864
	0.113	0.615	0.899	0.109	0.451	-0.187	0.092	1.195	-0.753	-1.435	0.402	-0.296
KLF1	-0.365	-0.099	-1.881	-0.598	-0.277	0.069	-1.932	-1.349	-4.15	-2.579	-4.027	-1.502
	0.481	-0.245	-1.888	-1.409	-0.596	-0.18	-1.994	-2.042	-3.103	-2.516	-3.133	-1.98
	-0.116	1.114	-1.742	-0.578	-0.18	-0.603	-2.146	-1.966	-4.489	-2.433	-3.431	-0.434
AHSP	0.099	-3.205	-1.312	-1.312	-0.922	0.256	-0.109	-1.287	-1.536	-0.998	-1.511	0.876
	-0.095	-2.733	-2.179	-1.264	-0.756	0.069	-0.102	-1.176	-1.925	-1.047	-1.982	0.931
	-0.005	-2.768	-1.409	-1.139	-0.714	0.159	-0.129	-1.155	-1.765	-0.929	-2.606	0.689
PRDX2	-0.058	2.405	1.338	0.076	0.813	0.169	0.538	3.158	-0.037	-1.349	1.215	-0.418
	0.102	2.488	1.844	0.783	0.896	-0.046	0.809	3.179	0.074	-0.081	1.59	-0.058
	-0.044	2.419	1.802	0.319	1.16	-0.739	0.809	3.152	0.039	-0.552	1.548	1.585
HBB	0	-2.133	-2.188	-1.647	-2.359	-1.167	-1.449	-3.597	-5.524	-5.367	-5.256	-2.294
	-0.014	-2.181	-2.16	-1.703	-2.338	-1.118	-1.469	-3.584	-5.538	-5.333	-5.173	-2.287
	0.014	-2.23	-2.181	-1.717	-2.324	-1.125	-1.657	-3.591	-5.511	-5.367	-5.277	-2.287
EPOR	-0.051	-1.913	-3.93	-0.908	-1.377	-0.441	-1.777	-1.777	-2.241	-3.313	-2.805	0.233
	-0.058	-1.164	-3.597	-1.047	-1.377	-0.448	-2.207	-1.181	-2.463	-3.14	-3.775	0.164

	0.109	-1.234	-3.854	-0.457	-1.474	-0.427	-2.338	-1.139	-2.061	-3.161	-3.664	0.261
E2F2	-0.085	-2.038	-2.641	-1.081	-2.444	-1.627	-1.569	-1.701	-2.934	-2.673	-3.346	-0.91
	0.032	-1.151	-2.1	-1.573	-1.904	-1.634	-1.631	-1.603	-3.468	-2.306	-3.255	-0.903
	0.053	-2.156	-2.391	-1.636	-1.862	-1.634	-1.576	-1.465	-3.246	-2.535	-3.311	-0.827
TWSG1	0.037	-1.354	-2.345	-1.132	-0.402	-0.021	-1.151	-1.065	-1.523	-2.338	-2.641	-0.85
	-0.109	-1.59	-1.992	-0.432	0.09	0.374	-1.13	-1.994	-1.516	-2.359	-3.098	-0.871
	0.072	-1.091	-2.588	-0.966	-0.263	0.658	-1.095	-2.389	-2.001	-2.324	-2.53	-0.927
HBG	0.067	-1.705	0.25	-2.523	-3.02	0.578	0.691	-0.571	1.668	1.929	-0.857	2.646
	-0.058	-1.774	0.173	-2.551	-2.867	0.612	0.441	-0.439	1.682	1.888	-0.92	2.465
	-0.009	-1.802	0.194	-2.495	-2.916	0.633	0.67	-0.432	1.682	1.874	-0.927	2.535
GF1B	-0.102	-3.198	-1.874	-1.104	-2.246	-0.721	-2.052	-1.141	-3.038	-2.359	-3.542	0.661
	-0.032	-2.99	-2.22	-1.264	-2.495	-0.707	-1.816	-1.578	-3.024	-2.331	-3.348	0.529
	0.134	-2.81	-2.456	-1.534	-2.53	-0.686	-2.1	-1.349	-3.121	-2.539	-3.549	0.689
TAL1	-0.042	-2.327	-2.243	-0.996	-2.428	-1.125	-1.631	-3.064	-3.276	-2.424	-3.537	-0.797
	0.069	-2.749	-2.257	-1.169	-2.401	-1.368	-1.617	-3.584	-3.332	-2.243	-3.565	-0.762
	-0.028	-2.472	-2.237	-1.363	-2.151	-1.257	-1.707	-3.577	-3.269	-2.354	-3.517	-0.839
LYZ	-0.002	-2.786	-3.604	-0.52	-2.486	-1.377	-1.744	-5.504	-3.292	-2.454	-5.249	-1.056
	0.136	-3.362	-3.549	-0.762	-2.749	-1.356	-1.751	-5.427	-3.265	-2.509	-5.236	-0.966
	-0.134	-3.057	-3.535	-0.485	-2.458	-1.349	-1.966	-5.365	-3.272	-2.495	-5.277	-0.98
C-MYB	0.89	-1.451	-2.068	-0.654	-0.527	-0.021	0.855	-1.287	-1.391	-1.213	-2.745	0.91
	-0.157	-1.167	-2.588	-0.259	-1.754	0.146	-0.018	-1.1	-1.883	-1.262	-2.391	0.924
	-0.732	-1	-1.472	-0.328	-1.719	-0.347	-0.317	-0.996	-1.661	-0.818	-2.398	0.869
BCL-xL	0.469	-1.899	-3.112	-1.816	-2.819	-2.084	-2.167	-1.61	-2.442	-3.175	-3.824	-0.91
	0.102	-1.906	-3.202	-1.878	-2.722	-2.098	-2.472	-1.271	-3.475	-3.805	-3.221	-0.952
	-0.571	-1.56	-2.572	-1.913	-2.507	-2.084	-2.798	-1.52	-3.052	-4.713	-4.115	-0.896
TUBB6	-0.402	-2.25	-2.015	-0.573	-2.803	-2.213	-2.213	-2.19	-3.72	-1.811	-2.664	-0.132
	0.028	-2.756	-1.696	-0.552	-2.99	-2.706	-2.2	-3.251	-3.477	-1.659	-3.385	-0.152
	0.374	-2.354	-3.061	-0.58	-2.622	-2.629	-2.213	-2.911	-3.609	-1.721	-4.133	-0.069
BCL11A	-0.053	-0.037	-2.983	-1.063	-2.516	-0.069	-1.148	-2.091	-3.6	-4.334	-3.23	-0.663
	-0.046	-0.404	-2.435	-0.751	-1.955	-0.007	-0.407	-2.084	-3.614	-2.893	-3.653	-0.927
	0.099	-0.654	-3.544	-1.215	-2.426	-0.236	-0.698	-1.64	-3.6	-3.225	-2.863	-0.829
Band3	-0.374	-0.497	-3.269	-1.71	-2.484	-1.042	-2.835	-0.624	-4.561	-3.731	-2.505	-2.176
	-0.291	-0.49	-2.105	-2.334	-2.754	-1.07	-1.906	-0.742	-4.367	-5.041	-2.31	-1.587
	0.665	-0.518	-1.71	-2.299	-2.553	-1.097	-1.712	-0.652	-2.974	-4.522	-2.491	-1.012
HSP70	0.575	-1.058	-1.412	-1.467	-2.234	-1.534	-2.128	-0.894	-2.669	-2.83	-1.43	-1.296

	0.506	-1.093	-0.975	-2.625	-1.853	-1.285	-1.768	-0.908	-2.149	-2.546	-1.451	-1.747
	-1.081	-1.245	-1.017	-1.433	-2.844	-2.304	-3.313	-0.88	-3.078	-1.992	-1.617	-2.003
UCHL1	0.344	-5.582	-4.512	-1.116	-2.798	-1.98	-1.134	-6.072	-3.362	-3.667	-5.439	-0.252
	-0.439	-5.714	-4.755	-1.178	-1.925	-1.439	-1.169	-5.358	-1.435	-3.32	-5.772	-0.293
	0.095	-5.631	-3.57	-1.352	-2.833	-0.947	-1.176	-5.954	-3.708	-3.327	-5.03	-0.349
GATA1	0.243	-2.049	-3.484	-1.176	-2.595	-1.576	-1.331	-1.601	-3.48	-2.622	-4.043	-1.518
	-0.097	-3.054	-3.325	-0.85	-3.052	-1.617	-1.67	-1.636	-3.168	-3.974	-4.113	-1.171
	-0.146	-1.994	-3.006	-1.072	-2.733	-1.534	-1.241	-1.684	-3.403	-2.137	-4.889	-1.934
Band4.1	0.155	0.483	-0.675	-0.772	-1.359	0.277	0.349	0.002	-1.557	-0.638	-1.081	-0.552
	0.072	0.462	-0.266	-1.25	-1.359	1.095	-1.197	-0.018	-1.051	-0.762	-1.449	0.723
	-0.226	0.462	-0.058	0.018	-1.081	0.825	-0.268	-0.088	-0.864	-0.561	-1.289	-1.335
Caspase-3	0.15	-0.589	-1.4	-0.832	-1.627	-0.552	-1.243	-0.806	-2.449	-1.53	-1.675	-1.077
	-0.03	-0.548	-1.074	-0.492	-1.585	-0.954	-1.326	-0.716	-2.248	-2.16	-1.717	-1.042
	-0.12	-0.444	-1.885	-0.887	-1.426	-1.495	-1.035	-0.619	-2.463	-1.315	-1.876	-0.494
SMAD1	0.192	-4.63	-4.103	-2.814	-2.84	-1.661	-1.079	-6.633	-3.385	-3.221	-6.76	-1.472
	0.025	-5.268	-4.277	-2.856	-3.165	-2.181	-1.121	-6.841	-4.341	-3.186	-6.948	0.303
	-0.217	-5.178	-4.318	-2.807	-3.068	-1.19	-1.086	-6.779	-3.995	-3.242	-6.67	-1.791
GATA2	-0.007	-1.086	-2.743	0.106	-1.957	-0.335	0.236	-2.814	-2.562	-2.671	-3.974	-0.118
	-0.055	-1.869	-2.756	-0.407	-1.673	-0.238	0.104	-2.793	-2.597	-2.664	-4.057	-1.56
	0.062	-1.668	-3.394	-0.116	-1.798	0.116	0.146	-2.62	-2.583	-0.973	-2.65	-1.317
Ank1	-0.139	-0.545	-1.433	-2.659	-1.818	-1.229	-2.793	2.218	1.067	-1.687	-2.109	0.866
	-0.277	-0.552	-1.356	-1.453	-1.534	-0.571	-2.384	2.308	1.123	-1.257	-2.2	0.693
	0.416	-0.483	-1.308	-0.622	-2.304	-0.585	-2.558	2.114	1.941	-0.106	-1.902	0.513
JAK2	0.201	-0.663	-0.219	0.03	-0.654	0.698	-1.338	-1.061	-0.894	-1.735	-1.423	-0.783
	-0.055	-0.663	0.023	0.848	-1.347	-0.515	-1.033	-0.749	-0.943	-1.52	-1.333	-1.74
	-0.146	-0.906	-0.476	-0.24	-1.541	0.344	-0.728	-1.199	-0.541	-0.127	-1.389	-1.74
TGFB3	0.287	-4.85	-2.844	-0.966	-2.371	-1.386	-1.878	-5.589	-3.295	-0.998	-4.087	-1.134
	-0.157	-4.968	-2.692	-0.98	-2.343	-1.289	-1.969	-5.575	-3.371	-0.797	-4.122	-1.218
	-0.129	-4.898	-3.08	-1.035	-2.447	-1.372	-1.927	-5.631	-3.357	-0.742	-4.101	-1.169
GYPA	-0.018	-0.779	-0.716	-0.39	-1.483	-0.049	-0.289	-0.587	0.945	1.705	-1.885	0.002
	-0.081	-0.924	-0.702	-0.307	-1.262	-0.042	-0.33	-0.642	0.973	1.657	-1.858	0.058
	0.099	-0.931	-0.689	-0.384	-1.199	-0.007	-0.275	-0.407	0.945	1.615	-1.871	0.079
SPTA1	-0.007	0.058	-0.531	-1.89	-1.811	-1.818	-2.294	0.021	-3.771	-3.399	-0.938	-2.329
	-0.021	0.176	-1.252	-1.003	-1.659	-1.804	-2.87	0.049	-2.523	-3.101	-1.07	-1.303
	0.028	0.106	-1.017	-0.518	-1.922	-2.463	-2.537	0.111	0.88	-2.934	-1.077	-1.962

FAS-L	-0.508	0.076	0.901	2.274	-2.479	0.28	-2.103	-3.953	0.012	1.257	-6.118	-0.952
	0.129	0.291	0.943	1.677	-4.787	0.411	-1.298	-2.207	-2.719	1.007	-5.792	-1.257
	0.379	0.284	0.776	1.164	-0.635	0.273	-0.508	-1.638	-1.389	1.167	-5.98	-1.361
STAT5	-0.462	-2.518	-2.019	1.516	-1.109	0.381	-0.291	-3.082	-2.001	-1.393	-4.36	0.002
	-0.31	-1.59	-1.846	1.391	-0.95	-0.18	1.476	-3.942	-2.5	-1.781	-3.507	0.529
	0.772	-0.737	-2.172	3.533	-1.248	0.374	-0.312	-2.521	-3.38	-1.532	-4.907	-0.171
Caspase-9	-0.058	-4.556	-4.464	-2.135	-2.451	-0.871	-1.724	-4.055	-4.055	-3.389	-4.868	-1.749
	0.039	-4.75	-4.388	-2.176	-2.424	-0.878	-2.105	-4.006	-4.034	-2.121	-4.903	-1.77
	0.018	-4.598	-4.45	-2.218	-2.382	-0.843	-1.737	-3.902	-3.958	-3.126	-4.875	-1.721
Cyclin-A	-0.065	-0.924	0.284	-0.173	-0.642	1.458	1.423	0.998	-1.449	-1.726	-0.065	-0.113
	0.06	-0.668	0.534	-0.728	-0.566	1.389	1.479	0.998	-1.282	-1.352	-0.335	0.76
	0.005	-0.626	0.326	-0.721	-0.628	0.765	1.042	0.998	-1.289	-1.282	-0.307	-0.675
SOX6	-1.245	0.016	-1.576	-0.723	-2.883	0.963	-0.887	-0.106	-2.518	-1.867	-2.084	-0.434
	0.356	0.016	-0.827	0.58	-1.913	0.991	-2.274	-0.12	-2.158	-0.689	-1.259	0.259
	0.89	0.335	-1.264	0.032	-0.963	0.714	-2.079	0.025	-3.378	-1.049	-2.015	-0.885
TNF	0.042	-4.797	-3.845	-1.162	-1.929	-1.236	-1.825	-5.584	-3.346	-2.611	-5.511	-1.303
	-0.028	-4.935	-3.789	-1.273	-1.895	-1.375	-1.798	-5.578	-3.366	-2.632	-5.427	-1.019
	-0.014	-5.018	-3.658	-1.682	-1.867	-1.298	-1.791	-5.619	-3.339	-2.604	-5.462	-1.074
NFE4	0.002	-3.158	-2.179	-1.59	-2.412	0.589	-0.18	-4.05	-1.562	-2.498	-3.304	0.813
	0.051	-3.921	-2.477	-1.402	-2.682	0.929	-0.513	-4.092	-1.999	-2.394	-3.408	0.245
	-0.053	-3.228	-1.853	-1.52	-2.495	0.721	-0.742	-4.009	-1.798	-2.449	-3.179	0.702
CDHBP	0.134	-1.675	-0.792	0.012	-0.638	1.587	1.061	-2.324	-1.084	0.095	-1.481	1.742
	-0.358	-1.543	-0.744	0.15	-0.423	0.014	-0.042	-2.172	-1.264	0.421	-1.627	1.694
	0.224	-1.412	-0.779	0.642	-0.97	0.804	0.513	-2.22	-1.895	0.24	-1.453	2.304
AKT	0.393	-2.671	-3.951	-0.36	-3.228	-1.8	-2.209	-2.073	-4.09	-1.511	-6.449	-0.543
	0.213	-3.018	-4.096	-0.187	-2.749	-1.848	-2.195	-2.974	-4.214	-2.87	-4.217	-0.356
	-0.605	-2.837	-4.076	-0.263	-2.847	-1.828	-2.223	-3.292	-4.152	-2.516	-4.196	-0.481
TFR1	0.113	0.931	-0.723	-0.217	-0.464	0.291	-0.007	0.698	-0.855	-0.903	-0.92	-0.018
	-0.039	0.973	-0.633	-0.3	-0.499	0.471	-0.423	0.649	-1.333	-0.952	-1.211	0.113
	-0.074	0.73	-1.056	-0.931	-0.811	-0.021	-0.478	0.518	-1.292	-1.756	-1.419	-0.622
NFE2	0.402	0.25	0.072	-0.829	-1.229	-0.626	-1.333	0.806	-1.218	-2.049	-0.658	-0.319
	-0.284	0.534	-0.039	-1.433	-0.536	-0.342	-0.827	0.661	-1.737	-1.599	-0.672	-1.248
	-0.118	0.312	-0.122	-0.774	-1.902	-0.869	-1.347	0.695	-2.576	-1.959	-0.541	-0.645
SMAD2	-0.028	0.007	-0.421	-0.649	-0.501	-1.222	0.462	-0.545	-1.939	-2.465	-1.144	-0.977
	0.007	-0.062	-0.809	-1.197	-1.409	-0.799	-0.515	-0.594	-3.103	-2.5	-1.13	-1.019

	0.021	-0.097	-0.58	-1.516	-0.98	-1.195	-0.37	-0.504	-2.153	-1.37	-0.998	-0.513
CDK2	-0.134	-4.681	-4.609	-3.126	-4.191	-1.987	-1.661	-2.89	-4.45	-3.022	-5.437	-0.453
	0.254	-5.035	-3.812	-1.296	-3.484	-1.994	-2.444	-2.717	-4.901	-2.641	-4.778	0.476
	-0.12	-6.643	-4.804	-3.216	-2.597	-2.722	-2.812	-2.953	-3.237	-2.176	-5.173	-1.791
GDF11	-0.176	-3.759	-1.726	-0.222	-2.486	-1.384	0.183	-5.178	-0.506	-0.208	-2.089	0.296
	0.095	-3.752	-0.548	-0.062	-2.59	-1.225	0.737	-5.621	-1.747	-1.386	-2.754	0.913
	0.081	-3.731	-2.572	1.095	-0.836	0.106	-0.483	-3.701	-1.185	-0.423	-3.433	1.599
LYAR	-0.439	-2.283	-1.345	-0.589	-1.959	-0.628	0.231	-1.463	-2.059	-1.67	-2.04	0.296
	-0.162	-1.943	-1.601	-0.818	-2.153	0.092	0.404	-1.456	-2.412	-0.977	-1.839	0.303
	0.601	-1.68	-0.658	-0.665	-1.779	-0.649	-0.296	-1.061	-1.733	-1.345	-2.151	-0.335
Caspase-8	-0.042	-3.362	-2.86	-0.538	-1.486	-0.28	-0.661	-3.997	-3.436	-0.074	-3.279	-0.187
	0.069	-2.391	-3.006	-2.354	-1.132	-0.085	-0.848	-3.373	-2.022	-1.509	-2.703	-0.478
	-0.028	-2.842	-4.392	-0.462	-0.917	0.393	-0.557	-4.053	-1.932	-1.952	-3.258	-1.469
GDF15	0.215	-4.984	-2.86	-0.587	-2.109	-1.298	-1.964	-4.17	-2.888	-2.528	-5.725	-1.061
	0.028	-5.039	-1.821	-0.573	-2.054	0.199	-2.165	-5.751	-3.165	-2.465	-5.289	-1.109
	-0.243	-4.942	-2.874	-0.684	-2.137	-1.597	-2.186	-5.335	-2.964	-2.625	-5.476	-1.074
CD79A	0.159	-3.57	-3.373	-1.502	-1.985	-1.215	-0.411	-5.349	-3.082	-2.542	-3.701	1.227
	0.076	-3.188	-3.45	-1.398	-2.019	-1.236	-1.167	-5.356	-3.089	-2.632	-3.688	1.282
	-0.236	-4.09	-3.422	0.633	-2.297	-1.222	-1.943	-5.453	-3.096	-2.916	-3.694	1.262
ZBTB7A	-0.046	-2.341	-3.725	-2.893	-1.927	-0.721	-0.721	-2.581	-3.371	-3.995	-2.909	-1.121
	0.009	-1.939	-3.073	-2.844	-2.377	-0.811	-0.776	-1.943	-3.08	-4.14	-2.521	-1.114
	0.037	-2.743	-2.754	-2.962	-1.781	-0.679	-0.818	-2.172	-2.678	-4.057	-3.124	-1.093
TRAIL	-0.208	-4.686	-3.269	-0.213	-2.193	-1.264	2.036	-4.226	-1.564	-2.59	-4.235	1.56
	0.125	-4.298	-3.262	-0.323	-2.255	-1.312	0.476	-4.261	-1.904	-2.444	-4.374	0.679
	0.083	-3.625	-3.45	-0.268	-2.172	-0.91	0.233	-4.17	-1.675	-2.417	-4.277	1.13
KLF3	-0.548	-0.139	-0.545	-0.414	0.996	0.067	0.545	-0.206	-2.153	0.827	0.596	-0.388
	-0.118	-0.076	0.356	2.248	0.33	-0.252	-0.411	0.002	-1.744	0.009	-0.7	2.073
	0.665	-0.055	-0.344	-0.878	0.608	2.479	0.885	-0.219	-0.677	0.896	-0.631	3.653
SMAD3	0.014	-1.213	-1.952	0.571	-1.347	-0.411	1.155	-3.658	-2.548	0.89	-2.682	-0.52
	-0.049	-2.211	-2.736	0.571	-0.716	0.795	1.107	-3.332	-1.446	0.127	-2.433	-0.513
	0.035	-1.927	-2.049	0.557	-1.139	0.393	1.134	-3.595	-1.911	-1.037	-2.156	-0.485

Supplemental Table 7: PCR primers used in this study

Primer	5'-sequence-3'	note
MluI-mCherrty+	TAGTCAATAATCAATGTCAAAAGGATCTGCGATCGCTCCG	amplification of mCherry
MluI-mCherrty-	TTCCTGCAACGCGTCTCCCCAGCATGCCTGCTATTG	
MluI-pegRNA+	GACGCGTTGCAGGAAGAGGGCCTATTTCCCATGATTCCTT	amplification of pegRNA
MluI-pegRNA-	CGCGATGTACGGGCCAGATATACGCGCCCAAGCTTAAAAA	
nick-MluI-F	TAGCAGGCATGCTGGGGAGAGATCCGACGCGCCATCTCTA	amplification of nick sgRNA
nick-MluI-R	GGCCCTCTTCCTGCAACGCGGACTAGTAAAAAAAGCACCG	
654/745-F	GACCAAATCAGGGTAATTTTGC	sanger sequencing for IVS-II-654/IVS-II-745 mutation
654/745-R	GGCCTAGCTTGGACTCAGAA	
745-RT-F	CTGAGGAGAAGTCTGCCGTTA	sanger sequencing for IVS-II-745 mutation (RT-PCR)
745-RT-R	GCCACCACTTTCTGATAGGC	
sampling-F	ACTCCTAAGCCAGTGCCAGA	sanger sequencing for beta-thalassemia mutation, except IVS-II-654/IVS-II-745
sampling-R	TCATTCGTCTGTTTCCCATTC	
beta-RT-all-F	CTGACACAACTGTGTTCACTAGCA	sanger sequencing for beta-thalassemia mutation, except IVS-II-745 (RT-PCR)
beta-RT-all-R	GATGCTCAAGGCCCTTCATA	
